# Lymphadenopathy and synovial hyperplasia are associated with sepsis risk in an experimental model of rheumatoid arthritis

**DOI:** 10.3389/fimmu.2025.1671137

**Published:** 2025-09-25

**Authors:** Johann Aleith, Wendy Bergmann-Ewert, Brigitte Müller-Hilke

**Affiliations:** Core Facility for Cell Sorting and Cell Analysis, Rostock University Medical Center, Rostock, Germany

**Keywords:** sepsis, infection, inflammation, autoimmunity, rheumatoid arthritis, hematopoiesis

## Abstract

Sepsis is a life-threatening condition arising from immune dysregulation, particularly in patients with underlying diseases like rheumatoid arthritis (RA). However, experimental data on this phenomenon are lacking. Using flow cytometry, we investigated immune responses in mice with or without collagen-induced arthritis (CIA) following *Streptococcus* infection. Mice without CIA effectively cleared the infection, maintained hematopoiesis, and mobilized lymphocytes. In contrast, CIA mice exhibited impaired bacterial clearance, leukopenia, and sepsis. Emergency hematopoiesis in CIA mice led to depletion of lineage-committed progenitor cells, correlating with an accumulation of immature neutrophils that exhibited diminished cytokinogenesis. Notably, immune dysregulation in CIA mice appeared before sepsis onset. We detected an increase in neutrophils and monocytes in draining lymph nodes and joints. Importantly, lymphadenopathy and hyperactivated synovial fibroblasts, along with articular immune cell infiltration, drove excessive cytokine production, increasing sepsis risk. Our findings emphasize the importance of rigorous medical management of RA to mitigate infection-related complications.

## Graphical Abstract

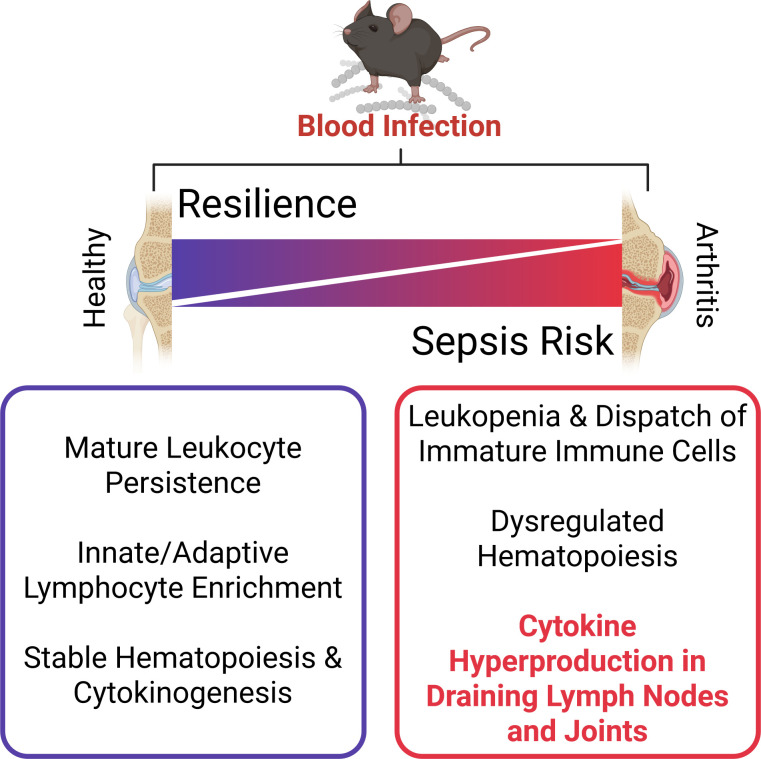

## 1 Introduction

Sepsis—defined as a dysregulated host response following infection that leads to organ dysfunction—is the leading cause of mortality in intensive care settings (1, 2). It is characterized by an inadequate release of pro-inflammatory cytokines upon activation of pattern recognition receptors (3). The hyperinflammatory phase transitions into systemic immunosuppression, driven by immune cell apoptosis, upregulation of anti-inflammatory effector functions, and maladaptive emergency hematopoiesis (4, 5). In sepsis, the maladaptive immune response fails to control bacterial dissemination and is often accompanied by immunosuppression, including leukocyte apoptosis and impaired recovery of immune cells due to exhausted emergency hematopoiesis (6, 7). Although the pathophysiology of sepsis has been extensively studied, mortality rates remain high, and progress in developing novel diagnostic and therapeutic tools has been limited (1, 8–10).

Sepsis predominantly occurs in patients with comorbidities and underlying conditions such as diabetes, malignancies, and rheumatoid arthritis (RA), which considerably increase the risk of fatal disease progression (11–13). RA is a prevalent autoimmune disorder that primarily affects the joints and has long been recognized to heighten infection risk and related complications (14–16). Notably, serious infections in RA patients carry an increased risk for sepsis, sepsis-related fatality, and long-term mortality following sepsis survival (17–20).

It has been debated whether this increased risk is linked to immunosuppressive therapy (21, 22). We previously demonstrated in a model of intravenous Streptococcus infection that immunocompetent mice with underlying collagen-induced arthritis (CIA) had a considerably increased risk of developing sepsis and septic arthritis, with a ninefold increase in mortality rate compared to infected mice without CIA (23). Conversely, mice with non-inflammatory joint disease resembling osteoarthritis were not more susceptible to sepsis (24). Collectively, our preliminary results suggest that the amplified sepsis risk in chronic inflammation extends beyond immunosuppression and joint damage alone and may instead be linked to the immunopathophysiology of the pre-existing disease itself (14).

Understanding the interaction between underlying inflammatory joint disease and infection might unlock new avenues for more individualized sepsis diagnostics and therapy in patients suffering from RA. This perspective underscores the need to further explore immune responses and hematopoietic changes in the context of autoinflammation-associated infection. Thus, we here set out to further explore sepsis immunobiology in RA. Specifically, we aimed to identify immune cell populations and cytokine production patterns that might correlate with increased sepsis risk For this, mice with and without CIA were infected and cell-type specific cytokine productions were investigated. Mice were sacrificed at two stages of infection, and cells from blood, liver, spleen, draining lymph nodes, joints, and bone marrow were analyzed by flow cytometry. This approach allowed us to identify potential immunological correlates that may contribute to sepsis susceptibility in RA.

## 2 Results

### 2.1 Sub-clinical sepsis in autoimmune arthritis was associated with impaired bacterial clearance and leukopenia

**Figure 1:**
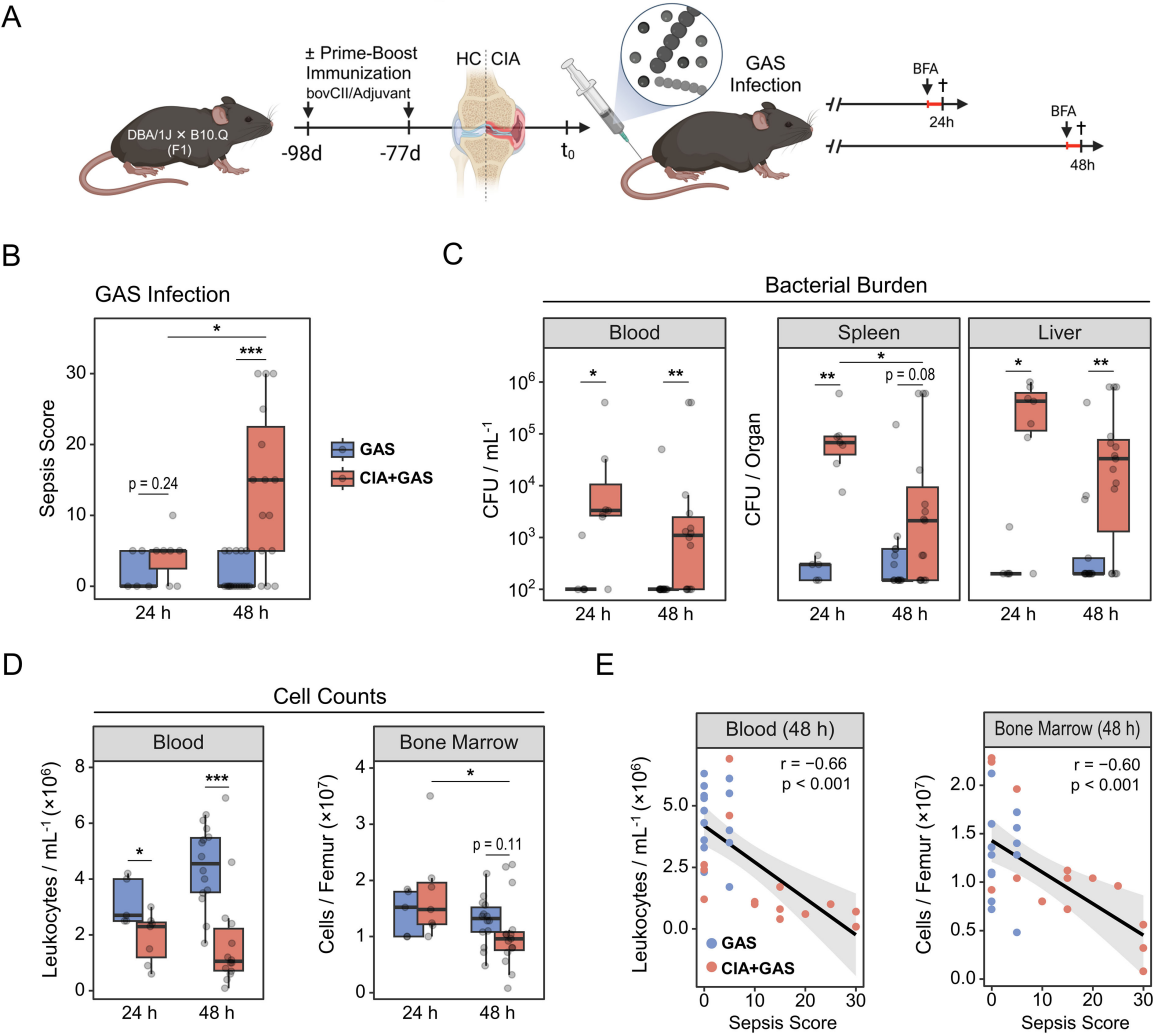
Group A Streptococcus (GAS) infection in an experimental model of rheumatoid arthritis. **(A)** Experimental study design scheme illustrating that first-generation (F1) offspring from DBA/1J × B10.Q crosses were subjected to prime-boost immunization using bovine collagen type 2 (bovCII) in adjuvant to elicit collagen-induced arthritis (CIA). Healthy control (HC) mice were not immunized. Eleven weeks after the booster immunization, animals were infected with GAS. Mice received a weight-adapted dose of Brefeldin A (BFA) either 20 or 44 hours post-infection and were sacrificed at 24 or 48 hours post-infection, respectively. **(B)** Box plot showing sepsis scores for mice without underlying conditions infected for 24 hours (GAS, n = 5) or 48 hours (GAS, n = 17), and for mice with pre-existing CIA infected for 24 hours (CIA+GAS, n = 7) or 48 hours (CIA+GAS, n = 15). **(C)** Box plots showing counts of β-hemolytic bacteria in peripheral blood, spleen and liver. **(D)** Box plots showing cell counts in peripheral blood and bone marrow, determined by trypan blue staining using a hemocytometer. Box plots indicate the median, with the lower and upper hinges corresponding to the 25^th^ and 75^th^ percentiles. Whiskers extend to the smallest and lowest values within 1.5 × interquartile range. **(E)** Scatter plots showing peripheral blood and bone marrow cell counts in relation to sepsis scores 48 hours post-infection. Each dot represents one animal. The black regression line follows a linear model with locally estimated scatter plot smoothing (LOESS), and the gray area represents the 95% confidence interval. r: Pearson correlation coefficient. p-values were obtained using Dunn’s test **(B–D)** or Pearson correlation analysis **(E)**. p < 0.05 (*), p < 0.01 (**), p < 0.001 (***).

To model rheumatoid arthritis, mice underwent prime-boost immunization with bovine type II collagen in adjuvant, while control mice were left untreated (**Figure 1A**). Collagen-induced arthritis (CIA) led to paw joint swelling and erythema, peaking at three weeks and declining after eight weeks post-boost. Eleven weeks post-immunization, CIA mice were randomized into groups for 24- or 48-hour intravenous Group A Streptococcus infections. The severity of CIA was comparable between mice assigned to the different infection durations (**Supplementary Figure S1A**). Notably, CIA mice exhibited significantly lower body weight than controls immediately before infection (t_0_), indicating compromised overall health, though weight-adjusted bacterial inoculums were comparable across all groups (**Supplementary Figure S1B**).

We next monitored the course of infection and observed that significant clinical manifestations of sepsis—measured by a scoring system incorporating weight loss, altered appearance, and abnormal behavior—emerged in CIA mice only at 48 hours post-infection (**Figure 1B**). Interestingly, bacterial burdens in peripheral blood, spleen, and liver were already increased in CIA mice at 24 hours post-infection (**Figure 1C**), indicating a maladaptive antibacterial immune response associated with the pre-existing disease. Similarly, arthritic mice tended to have a higher proportion of knee joint cavities invaded by bacteria (**Supplementary Table S1**).

While mice without underlying CIA exhibited a modest increase in blood cell counts at 48 hours post-infection, CIA mice developed cytopenia at both 24 and 48 hours. Similarly, bone marrow cellularity remained stable in control mice, whereas medullary cell counts decreased significantly over the course of infection in CIA mice (**Figure 1D**). Liver cell numbers were similar between groups, but an increase of spleen leukocytes over the course of infection was observed only in mice without CIA (**Supplementary Figure S1C**). Focusing on 48 hours post-infection, when sepsis became distinctive for CIA mice, we found significant negative correlations between the sepsis score and cell counts in blood, bone marrow, and spleen (**Figure 1E**; **Supplementary Figure S1D**).

Taken together, CIA mice exhibited early bacterial dissemination and failed to maintain cellular homeostasis following Streptococcus infection, which preceded the onset of sepsis.

### 2.2 Sepsis in arthritic mice was paralleled by an enrichment of non-classical myeloid cells in peripheral organs

**Figure 2:**
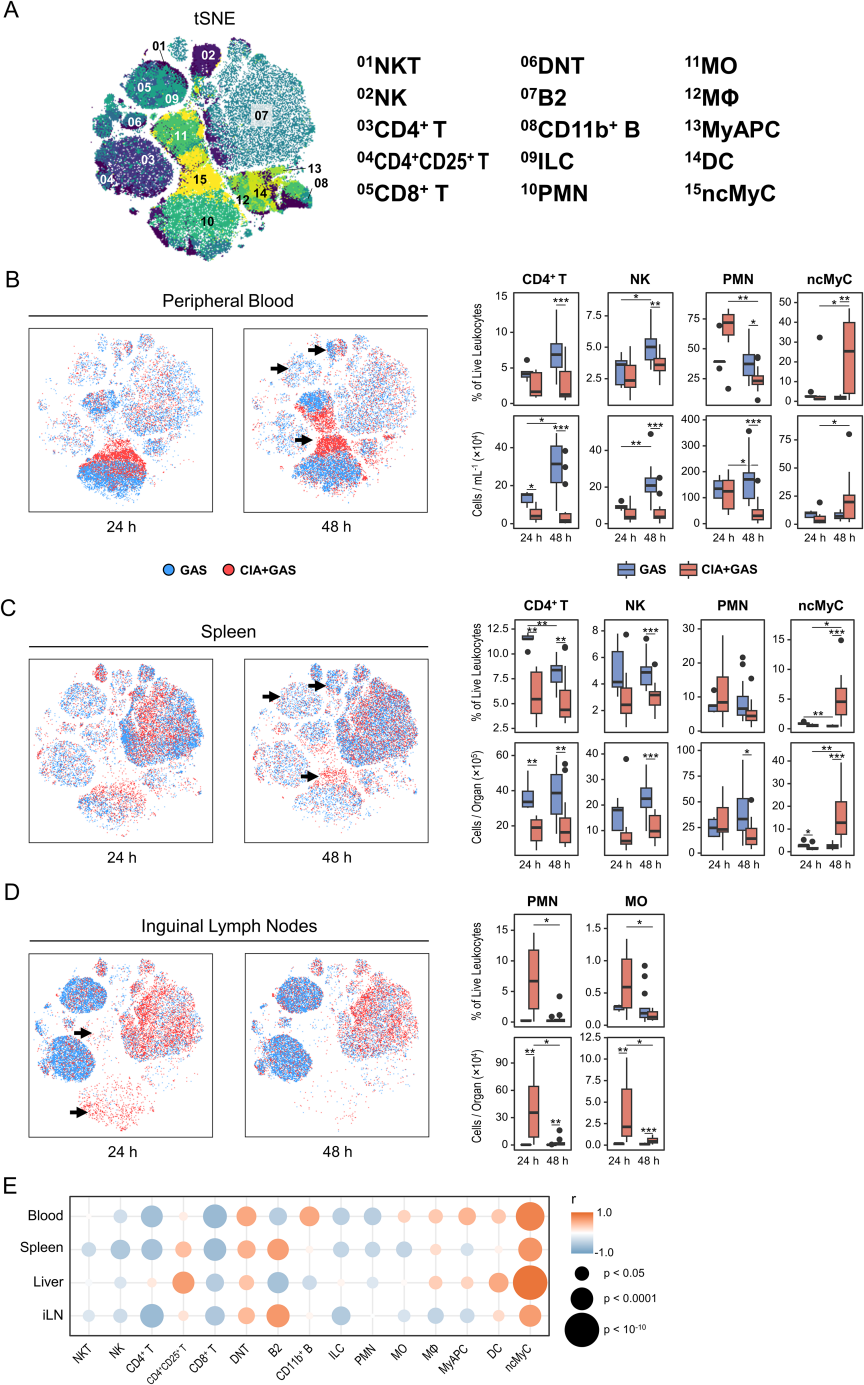
The enrichment of non-classical myeloid cells in various organs from arthritic mice correlated with the sepsis score. **(A)** Topological maps generated using t-distributed stochastic neighbor embedding (tSNE) illustrate immune cells identified by flow cytometry and hierarchical gating. Immune cell populations are indicated by color coding and numbered annotations. (**B−D**) Superimposed tSNE maps showing the immune landscape in peripheral blood **(B)**, spleen **(C)**, and iLN **(D)**, with black arrows indicating differences in immune cell compositions. Data include mice without underlying conditions infected for 24 hours (GAS, n = 5) or 48 hours (GAS, n = 14), and for mice with pre-existing CIA infected for 24 hours (CIA+GAS, n = 7) or 48 hours (CIA+GAS, n = 14). Box plots presenting frequencies and cell counts of CD4^+^ T lymphocytes, natural killer (NK) cells, polymorphonuclear neutrophils (PMN), and non-classical myeloid cells (ncMyC) for peripheral blood and spleen. For iLN, box plots show the frequencies and counts of PMN and monocytes (MO). Box plots indicate the median, with the lower and upper hinges corresponding to the 25^th^ and 75^th^ percentiles. Whiskers extend to the smallest and largest values within 1.5 × interquartile range. **(E)** Bubble plot demonstrating for peripheral blood (PB), spleen (Sp), liver (Lvr), and iLN the correlation between immune cell frequencies and sepsis scores at 48 hours post-infection. r: Pearson correlation coefficient. p-values were obtained using Dunn’s test **(B−D)**, or Pearson correlation analysis **(E)**. p < 0.05 (*), p < 0.01 (**), p < 0.001 (***).

We next sought to define sepsis-induced immunopathology in greater depth. To do this, we performed single-cell analyses on peripheral blood, spleen, liver, and inguinal lymph nodes (iLN) using a flow cytometry panel of antibody conjugates that covered a variety of markers for both innate and adaptive immune lineages. Through hierarchical gating (**Supplementary Figures S2A, B**), we identified 15 immune cell populations (**Figure 2A**). To gain an overview of our data, we compared their distribution across different peripheral organs. Regardless of the underlying condition or infection-associated pathology, blood samples were primarily composed of polymorphonuclear neutrophils (PMN) and monocytes (MO, **Supplementary Figure S2C**). The spleen was dominated by B cells, the liver contained a balanced mixture of lymphoid and myeloid cells, and iLN were enriched in B and T lymphocytes.

Stratifying the data by infection period and underlying condition, we identified distinct immune landscapes: one of them appeared indicative of a competent immune response leading to host resilience in mice without pre-existing CIA, and another one exhibited characteristic of immune dysregulation and sepsis in mice with CIA (**Supplementary Table S2**). Specifically, resilience was associated with an increase in lymphocytes—particularly CD4^+^ T cells, and natural killer (NK) cells—in blood, spleen, and liver (**Figures 2B, C**; **Supplementary Figure S2D**). In contrast, while PMN frequencies tended to increase in CIA mice 24 hours post-infection, they were depleted in both blood and spleen under sepsis conditions at 48 hours post-infection (**Figures 2B, C**). Notably, CD11b^+^Gr-1^lo/−^F4/80^−^CD11c^−^I-A/I-E^−^ non-classical myeloid cells (ncMyC, **Supplementary Figure S2B**) were significantly enriched in CIA mice 48 hours post-infection across all analyzed organs (**Figures 2B, C**; **Supplementary Figure S2D**, **Supplementary Table S2**).

Next, we focused on iLN, the secondary lymphoid compartment specifically draining the immunization site in CIA. Interestingly, the overall lymphocyte composition differed markedly between groups, potentially reflecting baseline differences between mice with and without CIA rather than infection alone (**Supplementary Figure S2E**). However, in CIA mice, PMN and MO frequencies and counts were higher at 24 hours post-infection (**Figure 2D**). We then conducted correlation analyses to examine whether alterations in the multiorgan immune landscape were associated with infection severity. Our data reveal throughout strong positive correlations between sepsis scores and the frequency of ncMyC in blood, spleen, liver, and iLN (**Figure 2E**).

In summary, an efficient immune response to bacterial infection was marked by lymphoid cell expansion, whereas sepsis in the context of arthritis was associated with the depletion of peripheral leukocytes that was paralled by an increase in non-classical myeloid cells across multiple organs.

### 2.3 Immature neutrophils exhibiting reduced cytokine expressions expanded during sepsis

**Figure 3:**
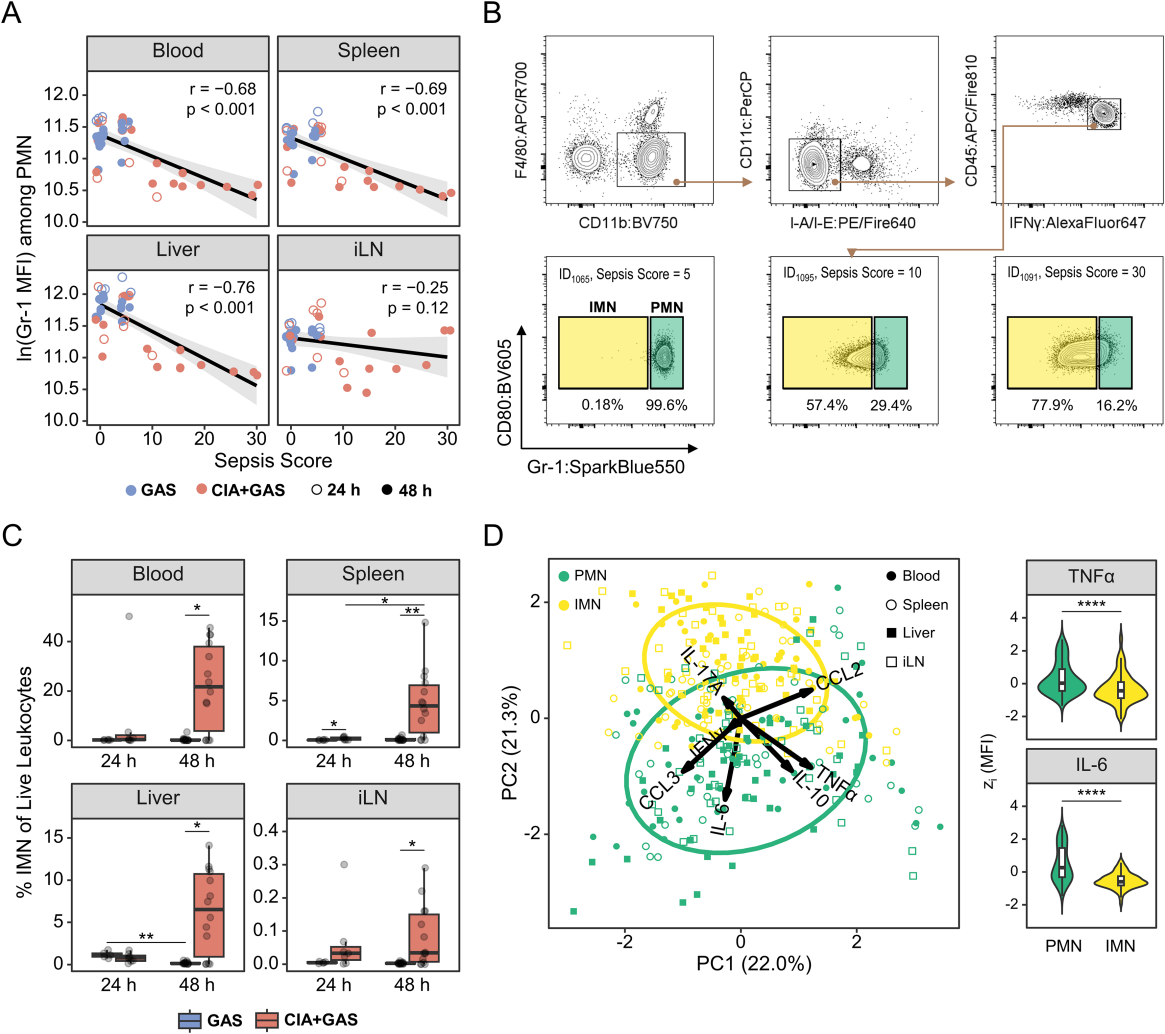
Sepsis in arthritic mice was paralleled by an enrichment of immature neutrophils featuring a decreased cytokine output. **(A)** Scatter plots depicting the relationship between Gr-1 expressions, based on the median fluorescence intensity (MFI) and sepsis scores in peripheral blood (PB), Spleen, Liver, and inguinal lymph nodes (iLN). Data include mice without underlying conditions infected for 24 hours (GAS, n = 5) or 48 hours (GAS, n = 14), and for mice with pre-existing CIA infected for 24 hours (CIA+GAS, n = 7) or 48 hours (CIA+GAS, n = 14). Each dot represents one animal. The black regression line follows a linear model with locally estimated scatterplot smoothing (LOESS), and the gray area represents the 95% confidence interval. **(B)** Top: Gating strategy for flow cytometry analyses of polymorphonuclear neutrophils (PMN) and immature neutrophils (IMN) across different organs. Bottom: Representative contour plots showing IMN frequencies for mice exhibiting different sepsis scores. **(C)** Box plots showing IMN frequencies in peripheral organs. **(D)** Principal component (PC) analysis illustrating the variance between PMN (green) and IMN (yellow), based on the median fluorescence intensities of intracellularly labeled tumor necrosis factor (TNF)α, interferon (IFN)γ, interleukin (IL-)6, IL - 17A, IL - 10, C-C motif chemokine ligand (CCL)2, and CCL3 – 24 and 48 hours post-infection. Violin and superimposed box plots depicting for PMN and IMN the expression of TNFα and IL - 6 across different organs, based on z(MFI). Box plots indicate the median, with the lower and upper hinges corresponding to the 25^th^ and 75^th^ percentiles. Whiskers extend to the smallest and largest values within 1.5 × interquartile range (IQR). r: Pearson correlation coefficient. p-values were obtained using Pearson correlation analysis **(A)**, Dunn’s test **(C)** or the Mann-Whitney U test **(D)**. p < 0.05 (*), p < 0.01 (**), p < 0.001 (***), p < 0.0001 (****).

Given the reported association between Gr-1 expression and neutrophil development (25), and the marked expansion of Gr-1^lo/−^ ncMyC in CIA-associated sepsis in our study, we suspected that a significant portion of this population comprised immature neutrophils (IMN). Supporting this, Gr-1 expression among PMN was lower in septic mice (**Figure 3A**). Since this reduction limited our ability to identify neutrophils by Gr-1 expression alone, we recently developed a gating strategy based on the observation that nearly all neutrophils, regardless of developmental stage, exhibit high intracellular interferon (IFN)γ expression (26). This approach allowed us to distinguish Gr-1^hi^ PMN from Gr-1^lo/−^ IMN via flow cytometry (**Figure 3B**; **Supplementary Figure S3A**). Using this method, we confirmed an increase in IMN frequencies across peripheral organs in CIA mice 48 hours post-infection (**Figures 3B, C**), with levels positively correlating with the sepsis score (**Supplementary Figure S3B**).

To assess the effector functions of PMN and IMN, we performed intracellular cytokine staining and found distinct expression patterns between these two cell populations (**Figure 3D**). Notably, IMN produced significantly lower levels of tumor necrosis factor (TNF)α, IFNγ, interleukin (IL)-6, and IL - 10 in blood, spleen, liver, and iLN, while CCL2 was elevated in spleen and liver (**Supplementary Figure S3C**; **Supplementary Table S3**). IL - 17A labeling was specifically increased in liver IMN (**Supplementary Figure S3D**). Importantly, while IMN were much less frequent in resilient mice (i.e., those without pre-existing CIA), their impaired effector function was independent of infection time and infection-associated immunopathology (**Supplementary Figure S3E**).

Taken together, our findings indicate that IMN expanded during sepsis in CIA mice and exhibited diminished cytokine production compared to their mature counterparts, suggesting impaired antibacterial effector functions.

### 2.4 Emergency hematopoiesis in sepsis stalled at the stage of myeloid- and lymphoid-biased progenitor cells

**Figure 4:**
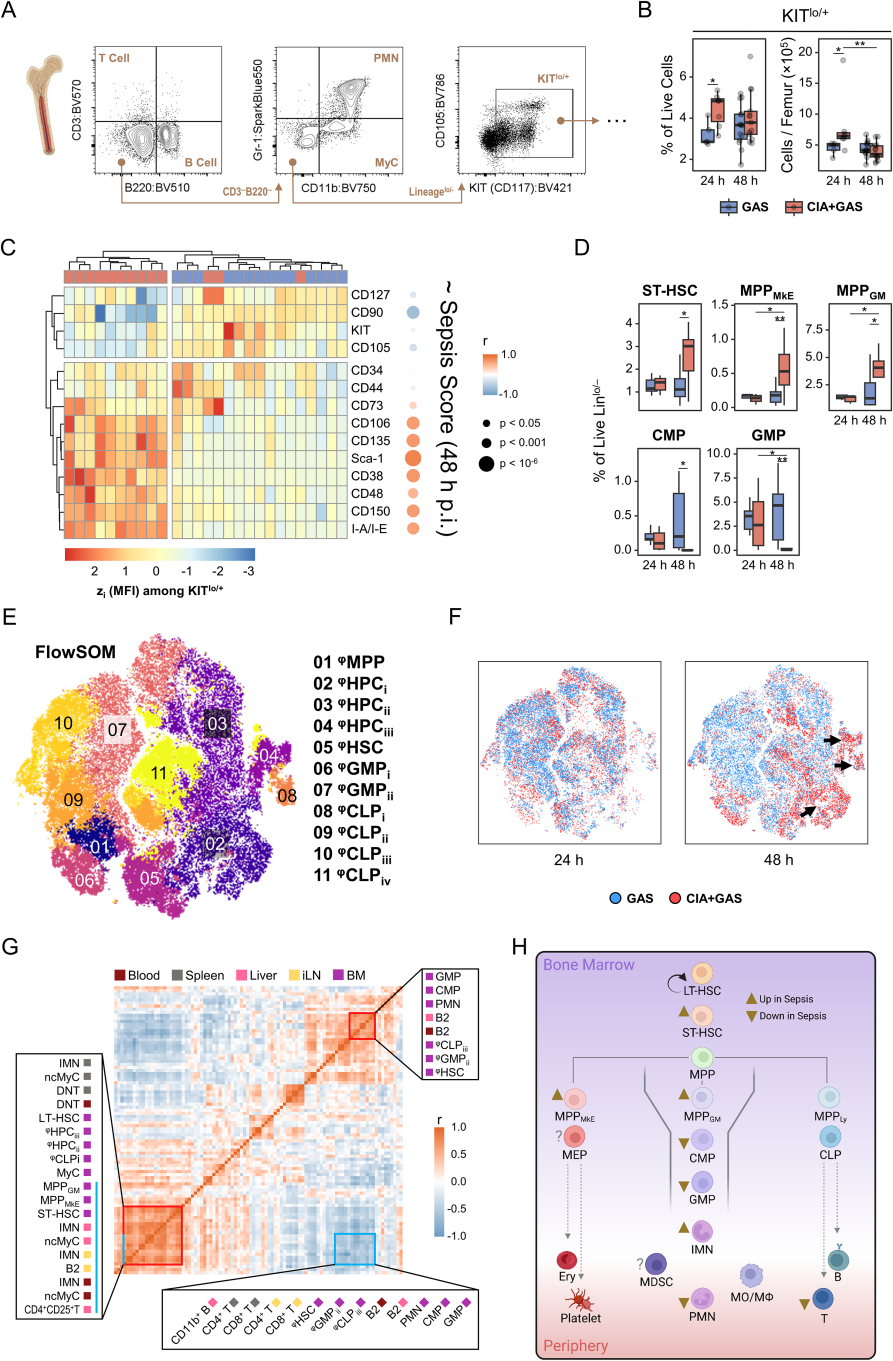
Characterizing the sepsis-specific medullary stem/progenitor cell compartment and its relationship with peripheral immune landscape restructuring. **(A)** Gating strategy for flow cytometry analyses of hematopoietic stem and progenitor cells in the bone marrow. **(B)** Box plots showing frequencies and cell counts of KITlo/hi cells in mice without underlying conditions infected for 24 hours (GAS, n = 5) or 48 hours (GAS, n = 14), and in mice with pre-existing CIA infected for 24 hours (CIA+GAS, n = 7) or 48 hours (CIA+GAS, n = 13). **(C)** Heatmap illustrating standardized (z) expression values of cell surface markers, based on median fluorescence intensities (MFI) among medullary KITlo/hi cells, and their correlation with sepsis scores at 48 hours post-infection. **(D)** Box plots presenting frequencies of manually-gated short-term hematopoietic stem cells (ST-HSC), multipotent progenitor (MPP) cells biased towards the megakaryocyte/erythrocyte (MkE) or granulocyte/monocyte (GM) lineage, common myeloid progenitor (CMP) cells, and granulocyte/monocyte progenitor (GMP) cells in the bone marrow. Box plots indicate the median, with the lower and upper hinges corresponding to the 25th and 75th percentiles. Whiskers extend to the smallest and largest values within 1.5 × interquartile range (IQR). **(E)** Topological map generated using t-distributed stochastic neighbor embedding (tSNE) illustrate KITlo/hi hematopoietic stem and progenitor cells identified by flow cytometry and flow self-organizing map (FlowSOM Cell populations are color-coded labeled with numbered annotations. **(F)** Superimposed tSNE maps showing the hematopoietic landscape stratified by infection time and pre-existing condition, with black arrows indicating differences in composition. **(G)** Heatmap representing the correlation matrix between frequencies of hematopoietic stem/progenitor cells in the bone marrow (purple) and peripheral immune cells in blood (dark red), spleen (gray), liver (pink), and inguinal lymph nodes (iLN, yellow). Red boxes highlight clusters of positively correlated cell populations, while the blue box marks populations negatively correlated with those labeled by the blue bar. **(H)** Flow chart illustrating the sepsis-induced alterations in medullary hematopoietic stem and progenitor cell frequencies. Arrowheads illustrate relative changes in frequencies among cell populations in septic CIA mice compare to infected control mice without CIA. MDSC, Myeloid-derived suppressor cells. r: Pearson correlation coefficient. p-values were obtained using Dunn’s test **(B, D)**, or Pearson correlation analysis **(C, G)**. p < 0.05 (*), p < 0.01 (**).

To link sepsis-associated alterations in the peripheral immune landscape to hematopoiesis, we performed single-cell analyses on medullary stem and progenitor cells. First, we identified immune lineage cells and observed a decrease in PMN frequencies alongside an increase in myeloid cells (MyC) in the bone marrow of CIA mice 48 hours post-infection (**Supplementary Figure S4A**). This shift coincided with a reduction in Gr-1 expression among PMN, which correlated with higher sepsis scores (**Supplementary Figure S4B**). We then analyzed the lineage^-^KIT^lo/+^ population (**Figure 4A**), which contains the majority of hematopoietic stem and progenitor cells (HSPCs) (27). Interestingly, this population transiently expanded in CIA mice at 24 hours post-infection but returned to levels that were comparable to resilient control mice by 48 hours (**Figure 4B**).

Conversely, the most striking alteration in hematopoiesis occurred in CIA mice at 48 hours post-infection, identified by the marked overexpression of stem cell markers such as Sca-1 and CD38, which correlated significantly with sepsis scores (**Figure 4C**). To determine whether these expression shifts translated into changes in specific HSPC subpopulations, we performed manual gating as previously described (7, 28, 29) (**Supplementary Figure S4C**). Notably, short-term hematopoietic stem cells (ST-HSCs) and multipotent progenitor cells biased toward megakaryocytes/erythrocytes (MPP_MkE_) and granulocytes/monocytes (MPP_GM_), respectively, were enriched in sepsis (**Figure 4D**). In contrast, common myeloid progenitors (CMP) and granulocyte-monocyte progenitors (GMP) were severely depleted in CIA mice at 48 hours post-infection.

Recognizing that manual gating only accounted for 30% of HSPC events (data not shown), we employed FlowSOM clustering on lineage^-^KIT^lo/+^ cells to gain a more comprehensive view of sepsis-induced emergency hematopoiesis (**Figure 4E**). FlowSOM-generated cellular subsets (^φ^HSPC) were phenotyped and annotated through hierarchical clustering. This allowed for the comparison of stem cell marker expression patterns among ^φ^HSPC with manually gated cells (**Supplementary Figure S4D**). Notably, sepsis led to an increase in ^φ^HPC subpopulations resembling MPP cells. While only CD127^hi φ^CLP_i_ cells were significantly increased, other lymphoid- and myeloid-biased progenitors were markedly depleted in CIA mice 48 hours post-infection (**Figures 4E, F**; **Supplementary Figure S4D, E**, **Supplementary Table S4**).

To integrate single-cell data from peripheral organs and bone marrow, we performed correlation analyses. This revealed that sepsis-induced alterations in the HSPC compartment were strongly linked to diminished lymphocyte frequencies and the increased mobilization of immature leukocytes (**Figure 4G**). Particularly, the depletion of myeloid- and lymphoid-biased progenitor cells in the bone marrow correlated significantly with increased frequencies of ncMyC/IMN across peripheral organs.

Collectively, our findings indicate that sepsis-induced hematopoietic dysfunction resulted in the depletion of immune cell progenitors, leading to a failure to replenish the peripheral leukocyte pool. The apparent bottleneck at the stage of myeloid- and lymphoid-biased progenitor cells suggests that emergency hematopoiesis in sepsis became progressively impaired, ultimately contributing to immune paralysis (**Figure 4H**).

### 2.5 Lymphadenopathy-driven cytokine overproduction was associated with sepsis risk in autoimmune arthritis

Systemic inflammation is a hallmark of sepsis, and we therefore investigated whether sepsis risk in autoimmune arthritis is related to aberrant cytokine production. To address this, we first identified cytokine-positive events among non-neutrophils using manual gating for TNFα, IFNγ, IL-6, IL-17A, IL-10, CCL2, or CCL3 and applied a Boolean *OR* operator to combine all cytokine-producing cells for subsequent analyses (**Supplementary Figures S5A, B**). Comparing cytokine expression across peripheral organs, we found that non-neutrophil leukocytes from the liver highly expressed various cytokines, including IL-6, IL-10, and CCL3 (**Supplementary Figure S5C**). In contrast, splenic cytokine production was less pronounced. Blood leukocytes primarily produced TNFα, IFNγ, and CCL2, while IL-17A was predominantly synthesized in the iLN (**Supplementary Figure S5C**).

**Figure 5:**
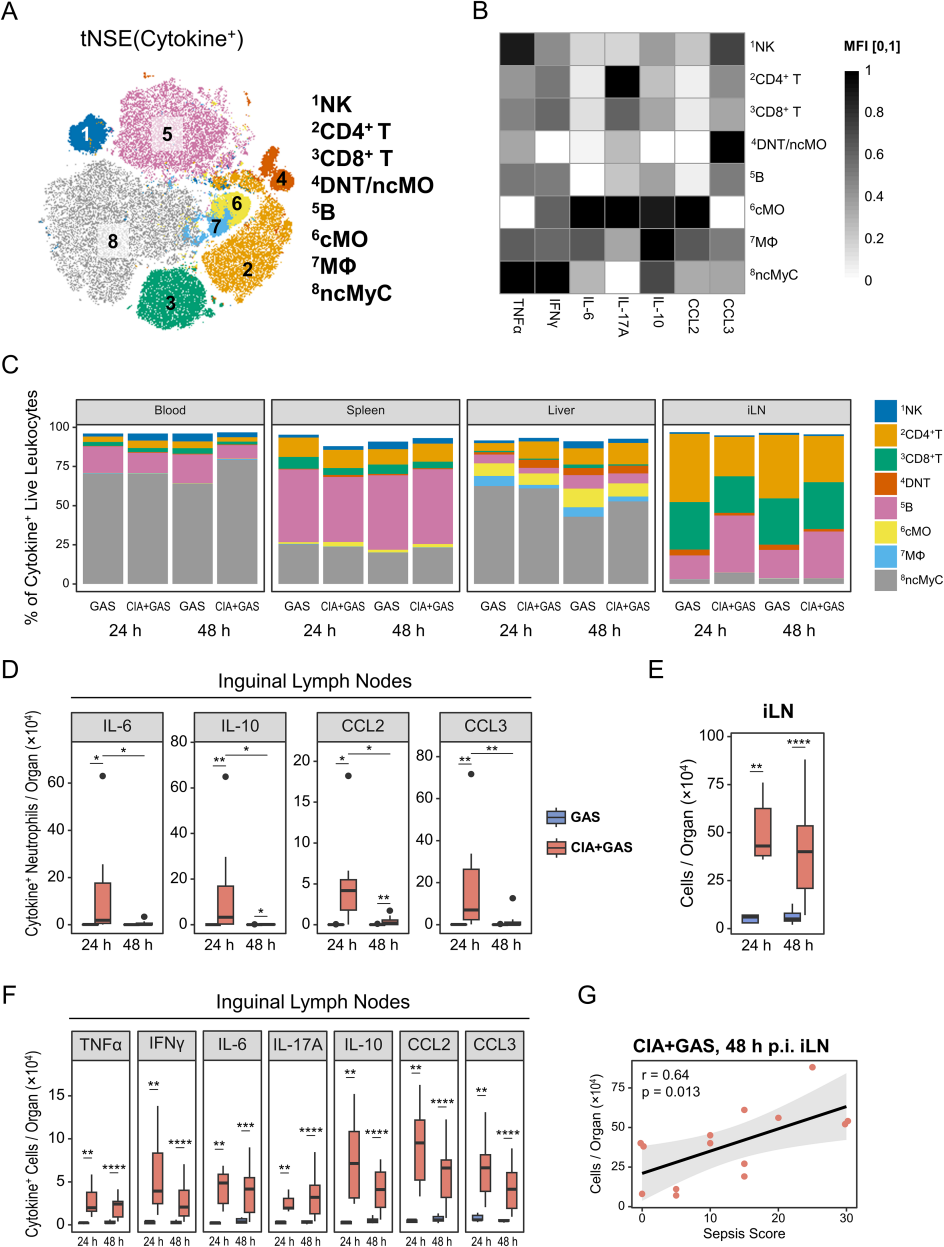
Associating sepsis risk in autoimmune arthritis with inguinal lymph node (iLN) cytokinogenesis. **(A)** Topological map generated using t-distributed stochastic neighbor embedding (tSNE) illustrate cytokine-producing immune cells identified by flow cytometry and flow self-organizing map (FlowSOM). Cell populations expressing cytokines are indicated by color coding and numbered annotations. **(B)** Grayscale heatmap showing intracellular expressions of (TNF)α, interferon (IFN)γ, interleukin (IL-)6, IL-17A, IL-10, C-C motif chemokine ligand (CCL)2, or CCL3 in FlowSOM-generated immune cell populations, based on median fluorescence intensities (MFI) normalized to a 0 – 1 range. **(C)** Stacked bar plots showing the composition of cytokine-producing cell types in different organs from mice without underlying conditions infected for 24 hours (GAS, n = 5) or 48 hours (GAS, n = 14), and for mice with pre-existing CIA infected for 24 hours (CIA+GAS, n = 7) or 48 hours (CIA+GAS, n = 14). **(D)** Box plots depicting cell counts of cytokine-producing neutrophils in iLN. **(E)** Box plot showing absolute cell numbers in iLN. **(F)** Box plots presenting counts of cytokine-expressing cells in iLN. Box plots indicate the median, with the lower and upper hinges corresponding to the 25^th^ and 75^th^ percentiles. Whiskers extend to the smallest and largest values within 1.5 × interquartile range (IQR). **(G)** Scatter plot highlighting the relationship between iLN cell counts and sepsis scores in CIA mice 48 hours post-infection. r: Pearson correlation coefficient. p-values were obtained using Dunn’s test **(C−E)** or Pearson correlation analysis **(F)**. p < 0.05 (*), p < 0.01 (**), p < 0.001 (***), p < 0.0001 (****).

To further characterize cytokine-producing immune subsets, we utilized FlowSOM clustering and annotated populations based on surface marker expression patterns (**Supplementary Figure S5D**). This analysis identified eight populations responsible for 94% of cytokine production across peripheral organs: NK cells, CD4^+^ and CD8^+^ T lymphocytes, double-negative T cells (DNT)/non-classical monocytes (ncMO), B lymphocytes, classical monocytes (cMO), macrophages (MΦ), and ncMyC (**Figure 5A**). Notably, ncMyC, which highly expressed TNFα, IFNγ, and IL-10, constituted the largest proportion of cytokine producers in the blood and liver. B lymphocytes were the primary source of TNFα, IFNγ, and CCL3 in the spleen, whereas IL-17A, TNFα, and IFNγ-producing CD4^+^ and CD8^+^ T lymphocytes were predominantly found in the iLN (**Figure 5B**; **Supplementary Figure S5E**).

Next, we compared cytokine-producing leukocytes between infected mice with and without CIA. While the compositions in blood, liver, and spleen were largely comparable across groups (**Figure 5C**; **Supplementary Table S5**), CIA mice exhibited significantly increased frequencies of TNFα, IL-6, IL-17A, IL-10, and CCL2-producing cells in the blood 48 hours post-infection (**Supplementary Figure S5F**). Changes in the spleen and liver were less pronounced, primarily involving differential TNFα and IL-10 productions (**Supplementary Figure S5G, H**). Given the above-described increase in PMN frequencies in the iLN 24 hours post-infection, we suspected these cells might contribute to nodal cytokine production. Indeed, we found for early infection in CIA mice an enrichment of cytokine-positive PMN in the iLN (**Figure 5D**).

Since adjuvant-chaperoned immunization and subsequent CIA led to an accumulation of cells in the draining lymph nodes (i.e., iLN, **Figure 5E**), we investigated whether this resulted in enhanced cytokine production in this organ. We observed a marked enrichment of cytokine-producing cells in the iLN as early as 24 hours post-infection, persisting until clinical sepsis development (**Figure 5F**). Furthermore, sepsis scores strongly correlated with iLN cell counts in septic mice (**Figure 5G**), suggesting that CIA-associated lymphadenopathy and excessive cytokine production contributed to immune dysregulation.

Overall, while cytokine-producing immune cells in the blood were only enriched at the clinical stage of sepsis, iLN leukocytes in CIA mice exhibited sustained cytokine overproduction before sepsis onset.

### 2.6 CIA-associated joint immune cell infiltration and synovial fibroblast activation accompanied articular cytokine overexpression in sepsis

**Figure 6:**
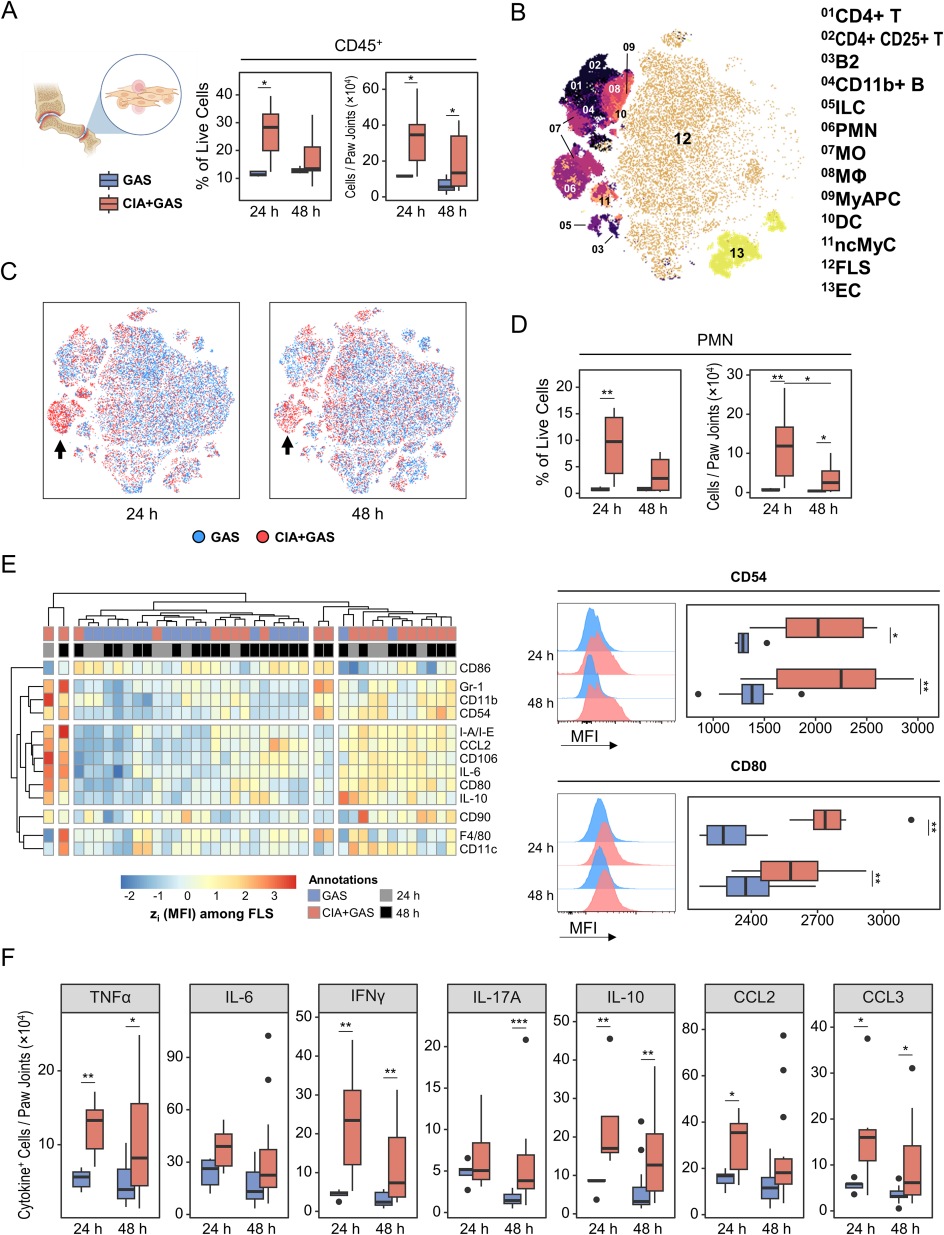
Linking immune cell infiltration in the arthritic joint to fibroblast-like synoviocyte (FLS) activation and cytokine production in sepsis susceptibility. **(A)** Box plots showing frequencies and counts of leukocytes in paw joints from mice without underlying conditions infected for 24 hours (GAS, n = 5) or 48 hours (GAS, n = 14), and for mice with pre-existing CIA infected for 24 hours (CIA+GAS, n = 7) or 48 hours (CIA+GAS, n = 14). **(B)** Topological map generated using t-distributed stochastic neighbor embedding (tSNE) illustrate cell populations in paw joints identified by flow cytometry and hierarchical gating. Cell populations are indicated by color coding and numbered annotations. **(C)** Superimposed tSNE maps showing the cellular composition in paw joints stratified by infection time and pre-existing condition, with black arrows indicating differences between mice with and without CIA. **(D)** Box plots depicting frequencies and counts of polymorphonuclear neutrophils (PMN) in paw joints. **(E)** Heatmap (left) illustrating standardized (z) expression values of surface protein markers, based on median fluorescence intensities (MFI) among FLS. Representative histograms and box plots (right) show the expression of CD54 (top) and CD80 (bottom), based on MFI among FLS. **(F)** Box plots presenting the counts of paw joint cells producing (TNF)α, interferon (IFN)γ, interleukin (IL-)6, IL-17A, IL-10, C-C motif chemokine ligand (CCL)2, or CCL3. Box plots indicate the median, with the lower and upper hinges corresponding to the 25^th^ and 75^th^ percentiles. Whiskers extend to the smallest and largest values within 1.5 × interquartile range (IQR). p-values were obtained using Dunn’s test. p < 0.05 (*), p < 0.01 (**), p < 0.001 (***).

Synovial hyperplasia and immune cell infiltration are common features of joint pathology in autoimmune arthritis. To determine whether joint cells contribute to immune dysregulation before or during sepsis, we enzymatically digested paw tissue from infected mice, analyzed the extracted articular cells by flow cytometry, and identified immune and stromal cell populations via manual gating (**Supplementary Figure S6A**). Although the time interval between collagen immunization and GAS infection allowed joint swelling to subside (**Supplementary Figure S1**), leukocyte infiltration remained pronounced in mice with underlying CIA (**Figure 6A**; **Supplementary Table S6**). In contrast, fibroblast-like synoviocyte (FLS) counts were comparable between groups (**Supplementary Figure S6B**).

Next, we examined joint cell composition and found a notable enrichment of PMNs in CIA mice 24 hours post-infection, which persisted—albeit at a lower level—until 48 hours post-infection (**Figure 6B–D**). Additionally, CIA joints exhibited a transient increase in monocytes at 24 hours post-infection, whereas CD4^+^ T cell enrichment was independent of infection time (**Supplementary Figure S6C**). Given that FLS constituted the majority of joint cells, we investigated their role in immune dysregulation. Indeed, FLS from CIA mice were hyperactivated, as indicated by increased expressions of cytokines, adhesion molecules such as CD54 (ICAM-1), and the co-stimulatory molecule CD80 (**Figure 6E**).

To further characterize cytokine-producing joint cells, we performed FlowSOM clustering on populations identified through Boolean gating. Lymphocytes, PMNs, antigen-presenting cells (APC), myeloid APCs (MyAPC), FLS, and endothelial cells were the primary sources of cytokine expression in the joint (**Supplementary Figure S6D**). When comparing the composition of cytokine-producing populations, we observed an increased frequency of IFNγ- and IL-10-producing PMNs, particularly in CIA mice at 24 hours post-infection (**Supplementary Figures S6E, F**). Notably, marked cytokine overexpression among all cell populations in CIA mice was evident for TNFα, IFNγ, IL-10, CCL2, and CCL3 as early as 24 hours post-infection (**Figure 6F**).

In summary, GAS infection in CIA mice was associated with hyperactivated FLS and persistent immune cell infiltration, contributing to cytokine overexpression and potentially increasing sepsis risk.

## 3 Discussion

Serious infections are a major cause of excess mortality in patients with RA (30, 31). While several observational studies have reported that immunosuppression caused by biological disease-modifying antirheumatic drugs (bDMARDs) significantly increases infection risk (21, 32, 33), data from the pre-bDMARD era suggest that RA itself may be inherently linked to an impaired immune response to pathogens (14). Moreover, retrospective single- and multi-center studies indicated that RA patients face an increased risk of developing sepsis and septic shock (17, 18), highlighting that immune responses in RA are not only insufficient for pathogen clearance but also dysregulated.

In this study, we investigated the immunological intricacies underlying sepsis in RA by employing the murine CIA model as an *in vivo* representation of the human disease. We demonstrated that mice with pre-existing chronic inflammatory joint disorder were unable to clear invading *Streptococci*. Conversely, mice without underlying autoimmunity rapidly eradicated the infection and exhibited an expansion of various lymphocyte subsets. During the early stages of infection (i.e., 24 hours post-infection), CIA mice developed peripheral lymphopenia caused by T cell depletion, while neutrophil counts remained stable. Lymphopenia is a common feature of sepsis arising from apoptosis, to which T cells are particularly susceptible, significantly contributing to immunosuppression (34–36). Consequently, lymphopenia is independently associated with worse outcomes and early mortality in sepsis patients (37, 38).

In the later stages of infection (i.e., 48 hours post-infection)—when sepsis developed exclusively in CIA mice—we observed a decline in peripheral blood neutrophils coinciding with an enrichment of CD11b^+^Gr-1^lo^ non-classical myeloid cells in various organs. Consistent with previous studies, we classified these cells as immature neutrophils (39, 40). For a more rigorous characterization of neutrophil development during sepsis, future studies should incorporate CXCR2 and CD101 as suggested by Evrard et al. (41). In our analyses, immature neutrophils produced fewer cytokines than their mature counterparts, suggesting reduced functionality. Neutrophils are essential for early pathogen containment due to their rapid migration to infected tissue and potent antibacterial activity (42). However, in sepsis, it has been reported that neutrophils can contribute to tissue damage through excessive extracellular trap formation and aberrant reactive oxygen species production (43, 44). In this context, immature neutrophils demonstrate increased NADPH oxidase expression, promoting oxidative bursts as discussed by previous studies (45, 46). Furthermore, according to studies on sepsis patients, immature neutrophils exhibit enhanced adhesion properties that may accelerate vascular integrity decline, while key functions such as phagocytosis and transmigration are diminished, particularly during septic shock (47, 48). These immature cells also appear more resistant to apoptosis, and their persistence may contribute to organ damage (49, 50). Accordingly, increased frequencies of immature neutrophils have been associated with a higher risk of sepsis-related death (51).

To meet the increased demand for circulating innate immune cells during infection, the stem cell compartment shifts into a state known as emergency granulopoiesis (42, 52). In our study, we linked the sepsis-induced release of immature myeloid cells, which occurred at the cost of mature neutrophil development, to an enrichment of hematopoietic stem cells and multipotent progenitors skewed towards myeloid differentiation. Conversely, the pool of common myeloid and granulocyte-monocyte progenitor (CMP/GMP) cells was markedly depleted. A previous study investigating lethal *P. aeruginosa* infection reported similar findings, demonstrating neutrophil depletion accompanied by expansion of hematopoietic stem cell subsets and exhaustion of myeloid-biased progenitor cells (53). While transiently reduced frequencies of CMP/GMP were also observed in the early stages of sepsis following cecal ligation and puncture, these populations expanded as the infection progressed (54). Another study using an analogous model demonstrated that sustained hematopoietic exhaustion in polymicrobial peritonitis was restricted to common lymphoid progenitors, whereas myeloid progenitors expanded (7). However, unlike our model, in which a sepsis-related lethal outcome typically occurs shortly after 48 hours post-infection (23), polymicrobial infection in the above-mentioned studies was associated with delayed mortality. This suggests that not only the mode of infection (i.e., monobacterial vs. polymicrobial) but also the trajectory of disease progression may shape hematopoietic dynamics, with persistent depletion of myeloid progenitors potentially contributing to the accelerated onset of septic shock.

While lymphopenia, the release of immature neutrophils (i.e., left shift), and distorted hematopoiesis are well-established features of sepsis (37, 45, 52), these phenomena are rarely investigated in combination and have not been studied extensively in models that reflect pre-existing autoimmune pathology. Our work addresses this gap by demonstrating that these hallmark features of sepsis emerge in a coordinated fashion in the context of chronic inflammatory disease.

Thus, our model of serious infection in the setting of underlying autoimmune arthritis recapitulates classical immunological features of sepsis while revealing additional immunopathological processes shaped by the pre-existing inflammatory disease. Notably, we demonstrated that CIA-associated lymphadenopathy and persistent synovitis correlated with an increased susceptibility to sepsis, an association that has remained underappreciated due to the lack of appropriate disease models. In particular, swollen lymph nodes, activated synovial fibroblasts, and joint-infiltrating immune cells, which are characteristic of RA (55–57), harbored considerable numbers of cytokine-producing cells and may contribute to the cytokine storm observed in CIA mice upon infection (23).

Our study primarily generated correlative data, and mechanistic insights remain limited, necessitating further investigation in future studies. For instance, it remains to be investigated whether lymphadenectomy in models of arthritis or other autoimmune disorders can reduce the risk of sepsis. For instance, systemic lupus erythematosus—a systemic autoinflammatory condition—is also associated with an increased risk for serious infections (58). Therefore, it remains unclear whether the increased sepsis risk in our model arises primarily from joint and nodal pathology, or from the broader systemic effects associated with adjuvant-chaperoned immunization and CIA. Similarly, other factors not investigated in our study may confound the relationship between lymphadenopathy, synovitis, and infection-related immune dysfunction. In particular, RA-associated comorbidities—such as cardiovascular and renal disease—have been reported to significantly increase the risk of sepsis (59, 60), and similar pathologies have been observed in CIA models, where chronic inflammation is associated with endothelial and cardiac dysfunction, as well as nephropathy (61–63).

Furthermore, it has been demonstrated that heat-killed *M. tuberculosis*, which was used as an adjuvant component for immunization in our study, can induce trained immunity in monocytes and macrophages (64). Trained immunity involves epigenetic and metabolic changes in innate immune cells following microbial exposure and activation of pattern-recognition receptors, leading to an amplified inflammatory response upon a subsequent challenge (65). While no specific data exist on enhanced immune responses against Group A Streptococcus following *M. tuberculosis* exposure, both pathogens share immunostimulatory motifs, particularly ligands that bind to Toll-like receptor (TLR)2 and Nucleotide-binding oligomerization domain (NOD)2 (66–68). Thus, future studies should explore the impact of CIA-associated comorbidities and trained immunity on immune dysregulation subsequent to invasive infection.

While our study focused on the infection-induced immune landscape in autoimmune arthritis, we did not include additional control mice to characterize the immune status of CIA animals prior to infection (i.e., at *t* = 0 h), which represents a limitation. Previous studies have shown that during acute CIA, lymphocyte populations accumulate in peripheral blood, spleen, and joint-draining lymph nodes (69–72). Although we cannot fully exclude the possibility that CIA-associated alterations in immune cell composition across different organs obscured the effect of infection, CIA was in remission in our model, and we suspect that long-term changes in immune cell composition were largely confined to the joints and joint-draining lymph nodes. Assessing baseline differences between CIA and healthy control mice remains the subject of ongoing research on immunopathophysiology in a broader context, which lies beyond the scope of the present study.

Furthermore, the translatability of our results is limited, as no observations on human subjects were included. While CIA recapitulates several key aspects of RA pathophysiology, it differs from the human disease in terms of etiology, the circulating autoantibody repertoire, disease chronicity, and heterogeneity (73). How these differences influence sepsis susceptibility remains unclear. Finally, we only studied male mice, as they are more prone to both CIA induction and GAS infection (74–76). Given that RA is more prevalent in women, and that male RA patients exhibit higher sepsis-related mortality (60), future studies should address sexual dimorphism as a potential determinant of sepsis risk in chronic joint inflammation. Taken together, our study indicates that lymphadenopathy and persistent synovitis are associated with an increased risk of sepsis in autoimmune arthritis. Notably, in RA, lymphadenopathy is most pronounced in joint-draining lymph nodes (56). While DMARD therapy primarily aims to reduce synovitis, it has been reported that swollen lymph nodes also respond to anti-inflammatory treatment (77, 78). Therefore, effective disease control in RA may contribute to a lower risk of sepsis. Supporting this, retrospective cohort studies have shown that the absence of swollen joints and reduced systemic inflammation are associated with a lower risk of serious infections (79, 80). Although bDMARDs generally increase infection risk (81), TNF inhibitors, in particular, have been linked to a lower prevalence of sepsis and reduced mortality after serious infections in RA patients, according to a prospective cohort study (60).

In conclusion, achieving complete and sustained remission in patients with RA is not only the primary goal of pharmacological treatment but may also significantly reduce infection-related complications and the risk of sepsis.

## 4 Methods

### 4.1 Animal model

DBA/1J and B10.D1-H2^q^/SgJ (B10.Q) mice were obtained from The Jackson Laboratory (Bar Harbor, US-ME) and continuously bred at the Rostock University Medical Center Animal Care Facility. Animals were housed in individually ventilated cages under specific pathogen-free conditions, maintained on a 12-hour light/dark cycle at 21 ± 2°C with 60 ± 10% humidity. Food and water were provided ad libitum. The F1 generation from a cross between female DBA/1J and male B10.Q mice was used for experiments. Male F1 mice were randomized using a dice-based random number generator into one of four groups: a control group (no immunization) or a collagen-induced arthritis (CIA) group scheduled for either 24-hour or 48-hour infections. From our own experience, F1 mice develop CIA with high incidence and with a severity and disease course comparable to that observed in DBA/1J mice (23, 82–84). While DBA/1J mice are the most commonly used strain for the CIA model, in our hands the incidence was consistently lower, which would have required substantially higher animal numbers. To adhere to the 3R principles and reduce animal use, we therefore chose the F1 model.

One week prior to immunization, animals at a minimum age of five weeks were transferred to conventional housing, including control mice. CIA group mice were anesthetized with isoflurane under oxygen flow. A total of 140 µg of bovine type II collagen (Chondrex, Redmond, US-WA), dissolved in 0.1 M acetic acid and emulsified in Freund’s adjuvant containing 240 µg *Mycobacterium tuberculosis* (Becton Dickinson, Franklin Lakes, US-NJ), was subcutaneously injected at the base of the tail, as previously described (23, 84). From this point, mice received 1 mg/mL tramadol (Ratiopharm, Ulm, Germany) in drinking water for analgesia until the experiment’s conclusion. Three weeks later, immunization was repeated with collagen in adjuvant without *Mycobacterium*. Subsequently, mice were monitored and scored for signs of arthritis, such as paw swelling and erythema, as previously reported (82).

Ten weeks after the second immunization, CIA and control mice were transferred to a facility for infection studies at the Rostock University Medical Center Microbiology Department. After another week, mice were infected with the *emm1* Group A Streptococcus (GAS) strain AP1 under isoflurane anesthesia, as previously described (23, 85). In brief, bacteria were cultured in Todd-Hewitt Broth (BD) until exponential growth was achieved, washed in PBS, and approximately 2 × 10^6^ colony-forming units (CFU) in 100 µL PBS were injected into the lateral tail vein.

Mice were subsequently monitored for signs of health impairment using an adapted murine sepsis score (23, 86). The summed score from four categories was used:

- Weight loss:
  ≥5% (5 points), ≥10% (10 points), ≥20% (30 points, humane endpoint).
- General appearance changes:
  Piloerection (5 points), hunched back or filthy orifices (10 points), cold to the touch (30 points, humane endpoint).
- Impaired consciousness or behavior:
  Lameness (5 points), self-isolation and lethargy (10 points), apathy and no reaction to stimuli (30 points, humane endpoint).
- Signs of inflammation or reduced respiration:
  Localized edema (5 points), disseminated edema or labored breathing (10 points), open wounds, or gasping (30 points, humane endpoint).

After 20 or 44 hours of infection, 10 mg/kg body weight Brefeldin A (Sigma-Aldrich, St. Louis, US-MO) was administered intraperitoneally. At 24 or 48 hours post-infection, mice were scored and received an intraperitoneally administered overdose of ketamine (150 mg/kg body weight, Livisto, Senden-Bösensell, Germany) and xylazine (10 mg/kg body weight, Bayer, Leverkusen, Germany). Blood samples were collected via cardiac puncture and mixed with EDTA. Mice were euthanized by cervical dislocation.

A medial arthrotomy was performed on both knee joints, followed by swabbing the synovium and plating the fluid on 5% sheep blood agar (BD). Inguinal lymph nodes (iLN) were removed and placed in a storage/staining buffer (SB; PBS with 0.5% BSA, Miltenyi Biotec, Bergisch Gladbach, Germany) containing 5 µg/mL Brefeldin A (Cayman Chemical, Ann Arbor, US-MI). The spleen, liver, fore- and hind-paws were excised, and the femur was separated from the tibia. All organs were stored in SB on ice. The spleen was passed through a 70 µm strainer (pluriSelect, Leipzig, Germany). The liver was weighed, and 0.4 g was minced using a scalpel and stored for later analysis. The remaining liver was passed through a cell strainer. Blood, spleen, and liver suspensions were plated on blood agar. Agar plates were incubated at 37°C overnight and counted.

### 4.2 Single-cell analysis

Most of the centrifugation steps were performed at 400 × *g* for 5 min. Two hundred microliters of anticoagulated blood was mixed with 5 mL of an in-house erythrocyte lysis buffer (0.15 M NH_4_Cl, 10 mM NaHCO_3_, 1 mM EDTA) and incubated on a rotary mixer at room temperature (RT) for 10 min. After centrifugation, blood cells were suspended in SB. Spleen samples were processed using 1X RBC Lysis buffer according to the manufacturer’s instructions (BioLegend, San Diego, US-CA). Minced liver samples were digested in 2 mL of pre-warmed RPMI - 1640 containing 10% FCS (PAN-Biotech, Aidenbach, Germany) and 1 mg/mL Collagenase/Dispase (Roche, Basel, Switzerland) at 37°C and 350 rpm for 30 min. The resulting suspension was filtered through a 100 µm strainer, centrifuged, washed, and suspended in SB. Red blood cell lysis was then performed as described for spleen samples. The iLN were passed through a 70 µm strainer and suspended in SB.

Joint cells were isolated as previously described (23). In brief, the claws of fore- and hind-paws were cut, and the skin was removed. The tendons were transected, followed by the removal of any remaining soft tissue. The paws were then separated from the brachium and tibia, and digested in 4 mL of DMEM containing 10% FCS (PAN-Biotech) and 240 U/mL Collagenase Type IV (STEMCELL Technologies, Vancouver, Canada) at 37°C and 400 rpm for 90 min on a magnetic stirrer. Tissue debris was removed by passing the suspension through a 100 µm strainer. The flow-through was centrifuged, and joint cells were suspended in SB.

Bone marrow was isolated as previously described (87). Briefly, the epiphyses of femora were removed, and bones were placed in a perforated tube. Cells were obtained by centrifugation at 10,000 × *g* for 15 s. The pellet was resuspended in 1X RBC lysis buffer and incubated as described for spleen and liver samples. Cell counts from all samples were determined using a hemocytometer, and 100,000 – 500,000 cells were used for subsequent steps.

Cells from all organs were washed in PBS, suspended in 1:2000 ZombieNIR (BioLegend), and incubated for 20 minutes at RT. Subsequently, cells were washed and suspended in SB. To block unspecific antibody-conjugate binding sites, cells were incubated with 10% FCS, 5% TruStain Monocyte Blocker, and 0.25 µg TruStain FcX PLUS (Hu IgG4 anti-mouse CD16/32, BioLegend) for 10 minutes on ice.

For blood, spleen, liver, and iLN samples (Panel 1), the following antibody-conjugates were added:

- 0.25 μg of CD3:BV480 (clone 17A2) and F4/80:APC/R700 (T45 - 2342, BD).
- 0.03 μg of CD4:BV650 (GK1.5), CD11b:BV750 (M1/70), I-A/I-E:PE/Fire640 (M5/114.15.2), CD49b:PerCP/Cy5.5 (HMα2), and CD45:APC/Fire810 (30-F11).
- 0.063 μg of Gr-1:SparkBlue550 (RB6 - 8C5).
- 0.125 μg of CD80:BV605 (16 - 10A1) and CD11c:PerCP (N418).
- 0.25 μg of B220:BV510 (RA3 - 6B2).
- 0.5 μg of CD25:PE/Dazzle594 (3C7, BioLegend).
- 0.15 μg of CD8α:PerCP/Vio700 (REA601) and CD86:APC/Vio770 (PO3.3, Miltenyi).
- 0.5 μg of CD127:eFluor450 (A7R34, Thermo Fisher Scientific, Waltham, US-MA).

For paw joint cells (Panel 2), the following antibody-conjugates were used:

- 0.25 μg of CD106:BV480 (429, BD) and F4/80:APC/R700 (as above).
- 0.015 μg of CD90.2:BV711 (30-H12).
- 0.03 μg of Gr-1:BV650 (RB6 - 8C5).
- 0.063 μg of CD4:SparkBlue550 (GK1.5) and CD54:PerCP/Cy5.5 (YN1/1.7.4).
- 0.25 μg of CD3:BV570 (17A2).
- B220:BV510, CD80:BV605, CD11b:BV750, CD25:PE/Dazzle594, I-A/I-E:PE/Fire640, CD11c:PerCP, CD45:APC/Fire810, CD8α:PerCP/Vio700, and CD86:APC/Vio770 (as above, BioLegend).
- 0.06 μg of CD31:SuperBright436 (390) and CD127:eFluor450 (as above, Thermo).

For bone marrow cells (Panel 3), the following antibody-conjugates were used:

- 0.13 μg of CD105:BV786 (MJ7/18).
- 0.25 μg of CD44:APC/R700 (IM7, BD).
- 0.06 μg of CD73:APC (Ty/11.8) and CD48:APC/Fire750 (HM48-1).
- 0.1 μg of CD127:PE/Cy5 (A7R37).
- 0.125 μg of KIT: BV421 (QA17A09), CD90.2:BV605 (30-H12), and CD106:PerCP/Cy5.5 (429).
- 0.25 μg of Sca-1:PE/Dazzle594 (D7).
- 0.5 μg of CD135:PE (A2F10), CD150:PE/Cy7 (TC15 - 12F12.2), and CD34:AlexaFluor647 (SA378A4).
- B220:BV510, CD3:BV570, CD11b:BV750, Gr-1:SparkBlue550, and I-A/I-E:PE/Fire640 (as above, BioLegend).
- 0.38 μg of CD38:VioBlue (REA616, Miltenyi).

Cells were incubated for 20 minutes on ice, followed by washing in SB. Bone marrow cells were centrifuged, suspended in autoMACS Running Buffer (RB, Miltenyi), and analyzed on the Aurora V16-B14-R8 cytometer running the SpectroFlo software v3.1.0 or v3.2.1 (Cytek Biosciences, Fremont, US-CA). Other cell samples were further processed using the Fixation Buffer and the 1X Intracellular Staining Permeabilization Wash Buffer per the manufacturer’s instructions (BioLegend). Unspecific antibody-conjugate binding sites were blocked as described above.

For intracellular cytokine staining, the following antibody-conjugates were added:

- 0.125 μg of TNFα:BV421 (MP6-XT22).
- 0.25 μg of IL-17A:BV785 (TC11-18H10.1), CCL2:PE (2H5), and IL-10:PE/Cy7 (JSE5-16E3, BioLegend).
- 0.075 μg of CCL3:APC (REA355).
- 0.15 μg of IL-6:FITC (REA1034, Miltenyi).

Cells were labeled for 20 minutes at RT. After washing with permeabilization buffer, cells were suspended in RB and analyzed on the Aurora cytometer as described above. Surface antigen staining using Panels 1 and 2, including intracellular labeling, was established and validated as recently described (88).

### 4.3 Statistics

Flow cytometry data were analyzed using FlowJo v10.10.0. Data analysis and visualization were performed in R v4.4.1 running on RStudio v2024.09.0. Contingency tables were analyzed using Fisher’s exact test. Normality was assessed using the Shapiro-Wilk test, with data considered normally distributed if p > 0.05. Normally distributed data were compared using ANOVA followed by the TukeyHSD test. For non-normally distributed data, the Dunn’s test was used. Statistical significance was defined as p < 0.05.

## Data Availability

The datasets presented in this study can be found in online repositories. The names of the repository/repositories and accession number(s) can be found in the article/**Supplementary Material**, https://sch197.med.uni-rostock.de/dl/DFG-SEPRA-IMMUN-AP1/.

## References

[1] Hodgin K Moss M. The epidemiology of sepsis. Curr Pharm Des. (2008) 14:1833–9. doi: 10.2174/138161208784980590, PMID: 18691094

[2] Singer M Deutschman CS Seymour C Shankar-Hari M Annane D Bauer M. The third international consensus definitions for sepsis and septic shock (sepsis-3). JAMA - J Am Med Assoc. (2016) 315:801–10. doi: 10.1001/jama.2016.0287, PMID: 26903338 PMC4968574

[3] Cavaillon J Adib-conquy M Fitting C Adrie C Payen D. Cytokine cascade in sepsis. Scand J Infect Dis. (2003) 35:535–44. doi: 10.1080/00365540310015935, PMID: 14620132

[4] Liu D Huang S-Y Sun J-H Zhang H-C Cai Q-L Gao C. Sepsis-induced immunosuppression: mechanisms, diagnosis and current treatment options. Mil Med Res. (2022) 9:56. doi: 10.1186/s40779-022-00422-y, PMID: 36209190 PMC9547753

[5] Venet F Monneret G. Advances in the understanding and treatment of sepsis-induced immunosuppression. Nat Rev Nephrol. (2018) 14:121–37. doi: 10.1038/nrneph.2017.165, PMID: 29225343

[6] Kwok AJ Allcock A Ferreira RC Cano-Gamez E Smee M Burnham KL. Neutrophils and emergency granulopoiesis drive immune suppression and an extreme response endotype during sepsis. Nat Immunol. (2023) 24:767–79. doi: 10.1038/s41590-023-01490-5, PMID: 37095375

[7] Terashima A Okamoto K Nakashima T Akira S Ikuta K Takayanagi H. Sepsis-induced osteoblast ablation causes immunodeficiency. Immunity. (2016) 44:1434–43. doi: 10.1016/j.immuni.2016.05.012, PMID: 27317262

[8] Cavaillon J Singer M Skirecki T. Sepsis therapies: learning from 30 years of failure of translational research to propose new leads. EMBO Mol Med. (2020) 12:e10128. doi: 10.15252/emmm.201810128, PMID: 32176432 PMC7136965

[9] Evans L Rhodes A Alhazzani W Antonelli M Coopersmith CM French C. Surviving sepsis campaign: international guidelines for management of sepsis and septic shock 2021. Intensive Care Med. (2021) 47:1181–247. doi: 10.1007/s00134-021-06506-y, PMID: 34599691 PMC8486643

[10] Schuurman AR Sloot PMA Wiersinga WJ van der Poll T. Embracing complexity in sepsis. Crit Care. (2023) 27:102. doi: 10.1186/s13054-023-04374-0, PMID: 36906606 PMC10007743

[11] Gotts JE Matthay MA. Sepsis: pathophysiology and clinical management. BMJ. (2016) 353:i1585. doi: 10.1136/bmj.i1585, PMID: 27217054

[12] Rhee C Jones TM Hamad Y Pande A Varon J O’Brien C. Prevalence, underlying causes, and preventability of sepsis-associated mortality in US acute care hospitals. JAMA Netw Open. (2019) 2:e187571. doi: 10.1001/jamanetworkopen.2018.7571, PMID: 30768188 PMC6484603

[13] Ibarz M Haas LEM Ceccato A Artigas A. The critically ill older patient with sepsis: a narrative review. Ann Intensive Care. (2024) 14:6. doi: 10.1186/s13613-023-01233-7, PMID: 38200360 PMC10781658

[14] Doran MF Crowson CS Pond GR O’Fallon WM Gabriel SE. Frequency of infection in patients with rheumatoid arthritis compared with controls: A population-based study. Arthritis Rheum. (2002) 46:2287–93. doi: 10.1002/art.10524, PMID: 12355475

[15] Goldenberg DL. Infectious arthritis complicating rheumatoid arthritis and other chronic rheumatic disorders. Arthritis Rheum. (1989) 32:496–502. doi: 10.1002/anr.1780320422, PMID: 2650687

[16] Mehta B Pedro S Ozen G Kalil A Wolfe F Mikuls T. Serious infection risk in rheumatoid arthritis compared with non-inflammatory rheumatic and musculoskeletal diseases: a US national cohort study. RMD Open. (2019) 5:e000935. doi: 10.1136/rmdopen-2019-000935, PMID: 31245055 PMC6560658

[17] Barrett O Abramovich E Dreiher J Novack V Abu-Shakra M. Short- and long-term mortality due to sepsis in patients with rheumatoid arthritis. Rheumatol Int. (2017) 37:1021–6. doi: 10.1007/s00296-017-3694-5, PMID: 28286904

[18] Krasselt M Baerwald C Petros S Seifert O. Mortality of sepsis in patients with rheumatoid arthritis: A single-center retrospective analysis and comparison with a control group. J Intensive Care Med. (2021) 36:766–74. doi: 10.1177/0885066620917588, PMID: 32249644 PMC8165740

[19] Sihvonen S Korpela M Laippala P Mustonen J Pasternack A. Death rates and causes of death in patients with rheumatoid arthritis: A population-based study. Scand J Rheumatol. (2004) 33:221–7. doi: 10.1080/03009740410005845 15370716

[20] Thomas E Symmons DPM Brewster DH Black RJ Macfarlane GJ. National study of cause-specific mortality in rheumatoid arthritis, juvenile chronic arthritis, and other rheumatic conditions: a 20 year followup study. J Rheumatol. (2003) 30:958–65., PMID: 12734889

[21] Bellan M Scotti L Ferrante D Calzaducca E Manfredi GF Sainaghi PP. Risk of severe infection among rheumatoid arthritis patients on biological DMARDs: A population-based cohort study. J Clin Med. (2022) 11:2955. doi: 10.3390/jcm11112955, PMID: 35683344 PMC9181346

[22] Cronstein BN Sitkovsky M. Adenosine and adenosine receptors in the pathogenesis and treatment of rheumatic diseases. Nat Rev Rheumatol. (2017) 13:41–51. doi: 10.1038/nrrheum.2016.178, PMID: 27829671 PMC5173391

[23] Volzke J Schultz D Kordt M Müller M Bergmann W Methling K. Inflammatory joint disease is a risk factor for streptococcal sepsis and septic arthritis in mice. Front Immunol. (2020) 11:579475. doi: 10.3389/fimmu.2020.579475, PMID: 33117382 PMC7576673

[24] Volzke J Müller-Hilke B. Degenerative joint damage is not a risk factor for streptococcal sepsis and septic arthritis in mice. Life. (2021) 11:794. doi: 10.3390/life11080794, PMID: 34440538 PMC8400161

[25] Aroca-Crevillén A Vicanolo T Ovadia S Hidalgo A. Neutrophils in physiology and pathology. Annu Rev Pathol: Mech Dis. (2024) 19:227–59. doi: 10.1146/annurev-pathmechdis-051222-015009, PMID: 38265879 PMC11060889

[26] Brunsch V Bergmann-Ewert W Müller-Hilke B Aleith J. Interleukin-6 overexpression and elevated granulocyte-to-lymphocyte ratio indicate hepatic stress in experimental group a Streptococcus sepsis. Med Microbiol Immunol. (2025) 214:17. doi: 10.1007/s00430-025-00826-2, PMID: 40178612 PMC11968515

[27] Morales-Mantilla DE Kain B Le D Flores AR Paust S King KY. Hematopoietic stem and progenitor cells improve survival from sepsis by boosting immunomodulatory cells. Elife. (2022) 11:1–20. doi: 10.7554/eLife.74561, PMID: 35166205 PMC8846591

[28] Challen GA Pietras EM Wallscheid NC Signer RAJ. Simplified murine multipotent progenitor isolation scheme: Establishing a consensus approach for multipotent progenitor identification. Exp Hematol. (2021) 104:55–63. doi: 10.1016/j.exphem.2021.09.007, PMID: 34648848

[29] Olender L Thapa R Gazit R. Isolation of murine myeloid progenitor populations by CD34/CD150 surface markers. Cells. (2022) 11:350. doi: 10.3390/cells11030350, PMID: 35159159 PMC8834359

[30] van den Hoek J Boshuizen HC Roorda LD Tijhuis GJ Nurmohamed MT van den Bos GAM. Mortality in patients with rheumatoid arthritis: a 15-year prospective cohort study. Rheumatol Int. (2017) 37:487–93. doi: 10.1007/s00296-016-3638-5, PMID: 28032180 PMC5357293

[31] Wang D Yeo AL Dendle C Morton S Morand E Leech M. Severe infections remain common in a real-world rheumatoid arthritis cohort: A simple clinical model to predict infection risk. Eur J Rheumatol. (2021) 8:133–8. doi: 10.5152/eurjrheum.2020.20172, PMID: 33372891 PMC9770411

[32] Singh JA Cameron C Noorbaloochi S Cullis T Tucker M Christensen R. Risk of serious infection in biological treatment of patients with rheumatoid arthritis: A systematic review and meta-analysis. Lancet. (2015) 386:258–65. doi: 10.1016/S0140-6736(14)61704-9, PMID: 25975452 PMC4580232

[33] Strangfeld A Eveslage M Schneider M Bergerhausen HJ Klopsch T Zink A. Treatment benefit or survival of the fittest: what drives the time-dependent decrease in serious infection rates under TNF inhibition and what does this imply for the individual patient? Ann Rheum Dis. (2011) 70:1914–20. doi: 10.1136/ard.2011.151043, PMID: 21791449 PMC3184240

[34] Hotchkiss RS Swanson PE Cobb JP Jacobson A Buchman TG Karl IE. Apoptosis in lymphoid and parenchymal cells during sepsis. Crit Care Med. (1997) 25:1298–307. doi: 10.1097/00003246-199708000-00015, PMID: 9267941

[35] Jensen IJ Sjaastad FV Griffith TS Badovinac VP. Sepsis-induced T cell immunoparalysis: the ins and outs of impaired T cell immunity. J Immunol. (2018) 200:1543–53. doi: 10.4049/jimmunol.1701618, PMID: 29463691 PMC5826615

[36] Zhang X Zhang Y Yuan S Zhang J. The potential immunological mechanisms of sepsis. Front Immunol. (2024) 15:1434688. doi: 10.3389/fimmu.2024.1434688, PMID: 39040114 PMC11260823

[37] Adigbli D Liu R Meyer J Cohen J Di Tanna GL Gianacas C. EARLY PERSISTENT LYMPHOPENIA AND RISK OF DEATH IN CRITICALLY ILL PATIENTS WITH AND WITHOUT SEPSIS. Shock. (2024) 61:197–203. doi: 10.1097/SHK.0000000000002284, PMID: 38151771

[38] Sheikh Motahar Vahedi H Bagheri A Jahanshir A Seyedhosseini J Vahidi E. Association of lymphopenia with short term outcomes of sepsis patients; a brief report. Arch Acad Emerg Med. (2019) 7:e14., PMID: 30847449 PMC6377227

[39] Panopoulos AD Zhang L Snow JW Jones DM Smith AM El Kasmi KC. STAT3 governs distinct pathways in emergency granulopoiesis and mature neutrophils. Blood. (2006) 108:3682–90. doi: 10.1182/blood-2006-02-003012, PMID: 16888100 PMC1895456

[40] Kim M-H Yang D Kim M Kim S-Y Kim D Kang S-J. A late-lineage murine neutrophil precursor population exhibits dynamic changes during demand-adapted granulopoiesis. Sci Rep. (2017) 7:39804. doi: 10.1038/srep39804, PMID: 28059162 PMC5216372

[41] Evrard M Kwok IWH Chong SZ Teng KWW Becht E Chen J. Developmental analysis of bone marrow neutrophils reveals populations specialized in expansion, trafficking, and effector functions. Immunity. (2018) 48:364–379.e8. doi: 10.1016/j.immuni.2018.02.002, PMID: 29466759

[42] Venet F Demaret J Gossez M Monneret G. Myeloid cells in sepsis-acquired immunodeficiency. Ann N Y Acad Sci. (2021) 1499:3–17. doi: 10.1111/nyas.14333, PMID: 32202669

[43] Denning N-L Aziz M Gurien SD Wang P. DAMPs and NETs in sepsis. Front Immunol. (2019) 10:2536. doi: 10.3389/fimmu.2019.02536, PMID: 31736963 PMC6831555

[44] Sônego F Castanheira FV e S Ferreira RG Kanashiro A Leite CAVG Nascimento DC. Paradoxical roles of the neutrophil in sepsis: protective and deleterious. Front Immunol. (2016) 7:155. doi: 10.3389/fimmu.2016.00155, PMID: 27199981 PMC4844928

[45] Demaret J Venet F Friggeri A Cazalis M-A Plassais J Jallades L. Marked alterations of neutrophil functions during sepsis-induced immunosuppression. J Leukoc Biol. (2015) 98:1081–90. doi: 10.1189/jlb.4A0415-168RR, PMID: 26224052

[46] Xie X Shi Q Wu P Zhang X Kambara H Su J. Single-cell transcriptome profiling reveals neutrophil heterogeneity in homeostasis and infection. Nat Immunol. (2020) 21:1119–33. doi: 10.1038/s41590-020-0736-z, PMID: 32719519 PMC7442692

[47] Drifte G Dunn-Siegrist I Tissières P Pugin J. Innate immune functions of immature neutrophils in patients with sepsis and severe systemic inflammatory response syndrome*. Crit Care Med. (2013) 41:820–32. doi: 10.1097/CCM.0b013e318274647d, PMID: 23348516

[48] Endo A Okamura M Yoshikawa S Otomo Y Morio T. Multilateral functional alterations of human neutrophils in sepsis: from the point of diagnosis to the seventh day. Shock. (2017) 48:629–37. doi: 10.1097/SHK.0000000000000883, PMID: 28430717

[49] Margraf A Lowell CA Zarbock A. Neutrophils in acute inflammation: current concepts and translational implications. Blood. (2022) 139:2130–44. doi: 10.1182/blood.2021012295, PMID: 34624098 PMC9728535

[50] Meghraoui-Kheddar A Chousterman BG Guillou N Barone SM Granjeaud S Vallet H. Two new neutrophil subsets define a discriminating sepsis signature. Am J Respir Crit Care Med. (2022) 205:46–59. doi: 10.1164/rccm.202104-1027OC, PMID: 34731593 PMC12042866

[51] Guérin E Orabona M Raquil M-A Giraudeau B Bellier R Gibot S. Circulating immature granulocytes with T-cell killing functions predict sepsis deterioration*. Crit Care Med. (2014) 42:2007–18. doi: 10.1097/CCM.0000000000000344, PMID: 24942511

[52] Paudel S Ghimire L Jin L Jeansonne D Jeyaseelan S. Regulation of emergency granulopoiesis during infection. Front Immunol. (2022) 13:961601. doi: 10.3389/fimmu.2022.961601, PMID: 36148240 PMC9485265

[53] Rodriguez S Chora A Goumnerov B Mumaw C Goebel WS Fernandez L. Dysfunctional expansion of hematopoietic stem cells and block of myeloid differentiation in lethal sepsis. Blood. (2009) 114:4064–76. doi: 10.1182/blood-2009-04-214916, PMID: 19696201 PMC2774548

[54] Fanti AK Busch K Greco A Wang X Cirovic B Shang F. Flt3- and Tie2-Cre tracing identifies regeneration in sepsis from multipotent progenitors but not hematopoietic stem cells. Cell Stem Cell. (2023) 30:207–218.e7. doi: 10.1016/j.stem.2022.12.014, PMID: 36652946

[55] Hasegawa M Sakai F Konda N Okabayashi A Katsura H Seto Y. CT assessment of axillary lymphadenopathy in patients with rheumatoid arthritis: association with disease activity and severity. Rheumatol Int. (2018) 38:1017–22. doi: 10.1007/s00296-018-3992-6, PMID: 29435630

[56] Rodolfi S Della-Torre E Bongiovanni L Mehta P Fajgenbaum DC Selmi C. Lymphadenopathy in the rheumatology practice: a pragmatic approach. Rheumatology. (2024) 63:1484–93. doi: 10.1093/rheumatology/kead644, PMID: 38109670 PMC11147542

[57] van Baarsen LGM de Hair MJH Ramwadhdoebe TH Zijlstra IAJ Maas M Gerlag DM. The cellular composition of lymph nodes in the earliest phase of inflammatory arthritis. Ann Rheum Dis. (2013) 72:1420–4. doi: 10.1136/annrheumdis-2012-202990, PMID: 23661491 PMC3711496

[58] Barrett O Abramovich E Dreiher J Novack V Abu-Shakra M. Mortality due to sepsis in patients with systemic lupus erythematosus and rheumatoid arthritis. Isr Med Assoc J. (2014) 16:634–5., PMID: 25438453

[59] Kimbrough BA Crowson CS Lennon RJ Davis JM Strangfeld A Myasoedova E. Multiple morbidities are associated with serious infections in patients with rheumatoid arthritis. Semin Arthritis Rheum. (2024) 65:152386. doi: 10.1016/j.semarthrit.2024.152386, PMID: 38244447 PMC10954402

[60] Richter A Listing J Schneider M Klopsch T Kapelle A Kaufmann J. Impact of treatment with biologic DMARDs on the risk of sepsis or mortality after serious infection in patients with rheumatoid arthritis. Ann Rheum Dis. (2016) 75:1667–73. doi: 10.1136/annrheumdis-2015-207838, PMID: 26567181 PMC5013078

[61] Denys A Clavel G Lemeiter D Schischmanoff O Boissier MC Semerano L. Aortic VCAM - 1: An early marker of vascular inflammation in collagen-induced arthritis. J Cell Mol Med. (2016) 20:855–63. doi: 10.1111/jcmm.12790, PMID: 26859834 PMC4831368

[62] Wang C Wei X Wu Y Tang H Wang B Wang Y. CP - 25 improves nephropathy in collagen-induced arthritis rats by inhibiting the renal inflammatory response. Int Immunopharmacol. (2020) 88:106997. doi: 10.1016/j.intimp.2020.106997, PMID: 33182042

[63] Zhou Z Miao Z Luo A Zhu D Lu Y Li P. Identifying a marked inflammation mediated cardiac dysfunction during the development of arthritis in collagen-induced arthritis mice. Clin Exp Rheumatol. (2020) 38:203–11. doi: 10.55563/clinexprheumatol/6kxs1o, PMID: 31140393

[64] Minute L Bergón-Gutiérrez M Mata-Martínez P Fernández-Pascual J Terrón V Bravo-Robles L. Heat-killed Mycobacterium tuberculosis induces trained immunity *in vitro* and *in vivo* administered systemically or intranasally. iScience. (2024) 27:108869. doi: 10.1016/j.isci.2024.108869, PMID: 38318361 PMC10838711

[65] Netea MG Domínguez-Andrés J Barreiro LB Chavakis T Divangahi M Fuchs E. Defining trained immunity and its role in health and disease. Nat Rev Immunol. (2020) 20:375–88. doi: 10.1038/s41577-020-0285-6, PMID: 32132681 PMC7186935

[66] Ferwerda G Girardin SE Kullberg B-J Le Bourhis L de Jong DJ Langenberg DML. NOD2 and toll-like receptors are nonredundant recognition systems of mycobacterium tuberculosis. PloS Pathog. (2005) 1:e34. doi: 10.1371/journal.ppat.0010034, PMID: 16322770 PMC1291354

[67] Heinhuis B Koenders MI van de Loo FA van Lent PLEM Kim S-H Dinarello CA. IL - 32γ and streptococcus pyogenes cell wall fragments synergize for IL - 1-dependent destructive arthritis via upregulation of TLR - 2 and NOD2. Ann Rheum Dis. (2010) 69:A47–8. doi: 10.1136/ard.2010.129635k, PMID: 20472585

[68] Wang X Fan X Bi S Li N Wang B. Toll-like receptor 2-and 4-mediated reciprocal th17 and antibody responses to group A streptococcus infection. J Infect Dis. (2016) 215:jiw598. doi: 10.1093/infdis/jiw598, PMID: 28011917

[69] Hu X Li F Zeng J Zhou Z Wang Z Chen J. Noninvasive low-frequency pulsed focused ultrasound therapy for rheumatoid arthritis in mice. Research. (2022) 2022:0013. doi: 10.34133/research.0013, PMID: 39290964 PMC11407525

[70] Teixeira JH Silva AM Almeida MI Bessa-Gonçalves M Cunha C Barbosa MA. The systemic immune response to collagen-induced arthritis and the impact of bone injury in inflammatory conditions. Int J Mol Sci. (2019) 20:5436. doi: 10.3390/ijms20215436, PMID: 31683648 PMC6862543

[71] Dahdah A Habir K Nandakumar KS Saxena A Xu B Holmdahl R. Germinal center B cells are essential for collagen-induced arthritis. Arthritis Rheumatol. (2018) 70:193–203. doi: 10.1002/art.40354, PMID: 29045049

[72] Wang F Tan W Guo D He S. Reduction of CD4 positive T cells and improvement of pathological changes of collagen-induced arthritis by FTY720. Eur J Pharmacol. (2007) 573:230–40. doi: 10.1016/j.ejphar.2007.07.029, PMID: 17716652

[73] Luan J Hu Z Cheng J Zhang R Yang P Guo H. Applicability and implementation of the collagen-induced arthritis mouse model, including protocols (Review). Exp Ther Med. (2021) 22:939. doi: 10.3892/etm.2021.10371, PMID: 34335888 PMC8290431

[74] Holmdahl R Jansson L Andersson M. Female sex hormones suppress development of collagen-induced arthritis in mice. Arthritis Rheum. (1986) 29:1501–9. doi: 10.1002/art.1780291212, PMID: 3801072

[75] Chella Krishnan K Mukundan S Alagarsamy J Laturnus D Kotb M. Host genetic variations and sex differences potentiate predisposition, severity, and outcomes of group A streptococcus-mediated necrotizing soft tissue infections. Infect Immun. (2016) 84:416–24. doi: 10.1128/IAI.01191-15, PMID: 26573737 PMC4730561

[76] Medina E Goldmann O Rohde M Lengeling A Chhatwals GS. Genetic control of susceptibility to group A streptococcal infection in mice. J Infect Dis. (2001) 184:846–52. doi: 10.1086/323292, PMID: 11550125

[77] Çalgüneri M Öztürk M Özbalkan Z Akdogan A Üreten K Kiraz S. Frequency of lymphadenopathy in rheumatoid arthritis and systemic lupus erythematosus. J Int Med Res. (2003) 31:345–9. doi: 10.1177/147323000303100415, PMID: 12964513

[78] Manzo A Benaglio F Vitolo B Bortolotto C Zibera F Todoerti M. Power Doppler ultrasonographic assessment of the joint-draining lymph node complex in rheumatoid arthritis: A prospective, proof-of-concept study on treatment with tumor necrosis factor inhibitors. Arthritis Res Ther. (2016) 18:242. doi: 10.1186/s13075-016-1142-7, PMID: 27770827 PMC5075165

[79] Accortt NA Lesperance T Liu M Rebello S Trivedi M Li Y. Impact of sustained remission on the risk of serious infection in patients with rheumatoid arthritis. Arthritis Care Res (Hoboken). (2018) 70:679–84. doi: 10.1002/acr.23426, PMID: 28960869 PMC5947836

[80] van Dartel SAA Fransen J Kievit W Flendrie M den Broeder AA Visser H. Difference in the risk of serious infections in patients with rheumatoid arthritis treated with adalimumab, infliximab and etanercept: results from the Dutch Rheumatoid Arthritis Monitoring (DREAM) registry. Ann Rheum Dis. (2013) 72:895–900. doi: 10.1136/annrheumdis-2012-201338, PMID: 22887849

[81] Listing J Strangfeld A Kary S Rau R von Hinueber U Stoyanova-Scholz M. Infections in patients with rheumatoid arthritis treated with biologic agents. Arthritis Rheum. (2005) 52:3403–12. doi: 10.1002/art.21386, PMID: 16255017

[82] Ebbers M Lübcke PM Volzke J Kriebel K Hieke C Engelmann R. Interplay between P. gingivalis, F. nucleatum and A. actinomycetemcomitans in murine alveolar bone loss, arthritis onset and progression. Sci Rep. (2018) 8:15129. doi: 10.1038/s41598-018-33129-z, PMID: 30310087 PMC6181973

[83] Lübcke PM Ebbers MNB Volzke J Bull J Kneitz S Engelmann R. Periodontal treatment prevents arthritis in mice and methotrexate ameliorates periodontal bone loss. Sci Rep. (2019) 9:8128. doi: 10.1038/s41598-019-44512-9, PMID: 31148565 PMC6544621

[84] Schmidt CJ Wenndorf K Ebbers M Volzke J Müller M Strübing J. Infection with clostridioides difficile attenuated collagen-induced arthritis in mice and involved mesenteric treg and th2 polarization. Front Immunol. (2020) 11:571049. doi: 10.3389/fimmu.2020.571049, PMID: 33193352 PMC7662472

[85] Aleith J Brendel M Weipert E Müller M Schultz D Müller-Hilke B. Influenza A virus exacerbates group A streptococcus infection and thwarts anti-bacterial inflammatory responses in murine macrophages. Pathogens. (2022) 11:1320. doi: 10.3390/pathogens11111320, PMID: 36365071 PMC9699311

[86] Shrum B Anantha RV Xu SX Donnelly M Haeryfar S McCormick JK. A robust scoring system to evaluate sepsis severity in an animal model. BMC Res Notes. (2014) 7:233. doi: 10.1186/1756-0500-7-233, PMID: 24725742 PMC4022086

[87] Amend SR Valkenburg KC Pienta KJ. Murine hind limb long bone dissection and bone marrow isolation. J Visualized Experiments. (2016) 110:53936. doi: 10.3791/53936, PMID: 27168390 PMC4941920

[88] Aleith J Bergmann-Ewert W Müller-Hilke B. Maximizing insights, minimizing animal testing: A framework for validating multiparametric single-cell cytokine analysis panels. Eur J Immunol. (2025) 55:e202451193. doi: 10.1002/eji.202451193, PMID: 40071676 PMC11898573

