# Supplementary material for: Lymphadenopathy and synovial hyperplasia are associated with sepsis risk in an experimental model of rheumatoid arthritis
Source: Front Immunol. 2025 Sep 25;16:1671137. doi: 10.3389/fimmu.2025.1671137 (PMC12507644; doi:10.3389/fimmu.2025.1671137)
Supplement: Supplementary file 1 [file DataSheet1.docx]

Supplementary Material

# Supplementary Figures


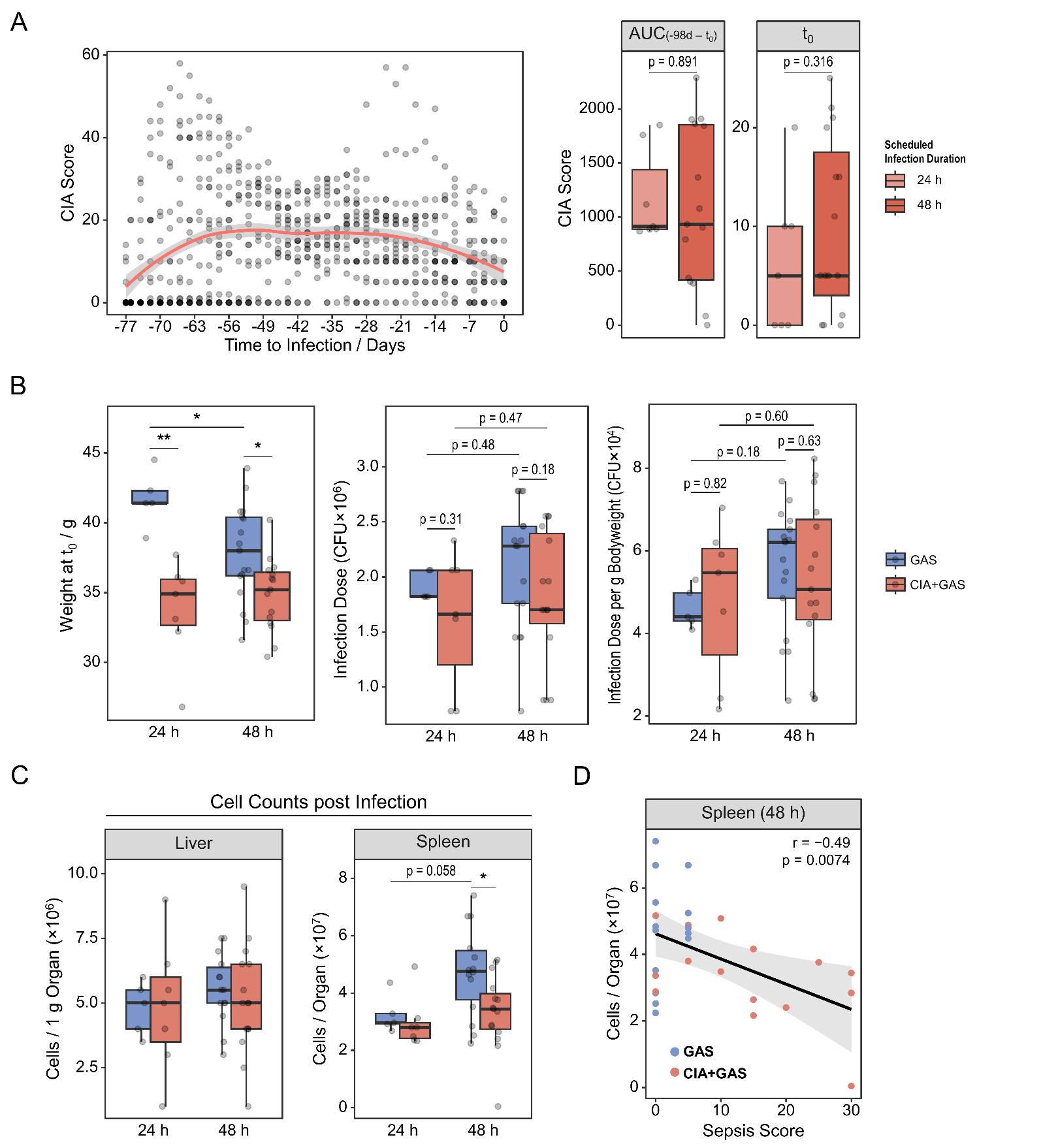


**Supplementary Figure S1** (on previous page)**.** Clinical features of collagen-induced arthritis and *Streptococcus* infection. **(A)** Scatter plot (left) illustrating the progression of collagen-induced arthritis (CIA, n = 22). Each dot represents the CIA score of one animal at a given time point. The red line shows mean CIA scores over time using locally estimated scatterplot smoothing (LOESS). Box plots (right) summarizing cumulative CIA scores (area under the curve, AUC) and CIA scores on the day of infection (t₀) for animals assigned to different infection schedules (n_24h_ = 7, n_48h_ = 15). **(B)** Boxplots showing body weight (left), received infection doses (middle), and weight-normalized infection doses (right) at t₀. Groups consisted of mice without underlying conditions infected for 24 hours (GAS, n = 5) or 48 hours (GAS, n = 17), and mice with pre-existing CIA infected for 24 hours (CIA+GAS, n = 7) or 48 hours (CIA+GAS, n = 15). **(C)** Boxplots displaying cell counts in the liver (left) and spleen (right) measured after infection. Boxplots indicate the median, with the lower and upper hinges corresponding to the 25^th^ and 75^th^ percentiles. Whiskers extend to the largest and smallest values within 1.5 × IQR (interquartile range). **(D)** Scatter plot showing spleen cell counts in relation to sepsis scores 48 hours post-infection. Each dot represents one animal. The black regression line follows a linear model with LOESS, and the gray area represents the 95% confidence interval. r: Pearson correlation coefficient. p-values were obtained using Tukey’s HSD test (A–C) or Pearson correlation analysis (D). p < 0.05 (*), p < 0.01 (**).


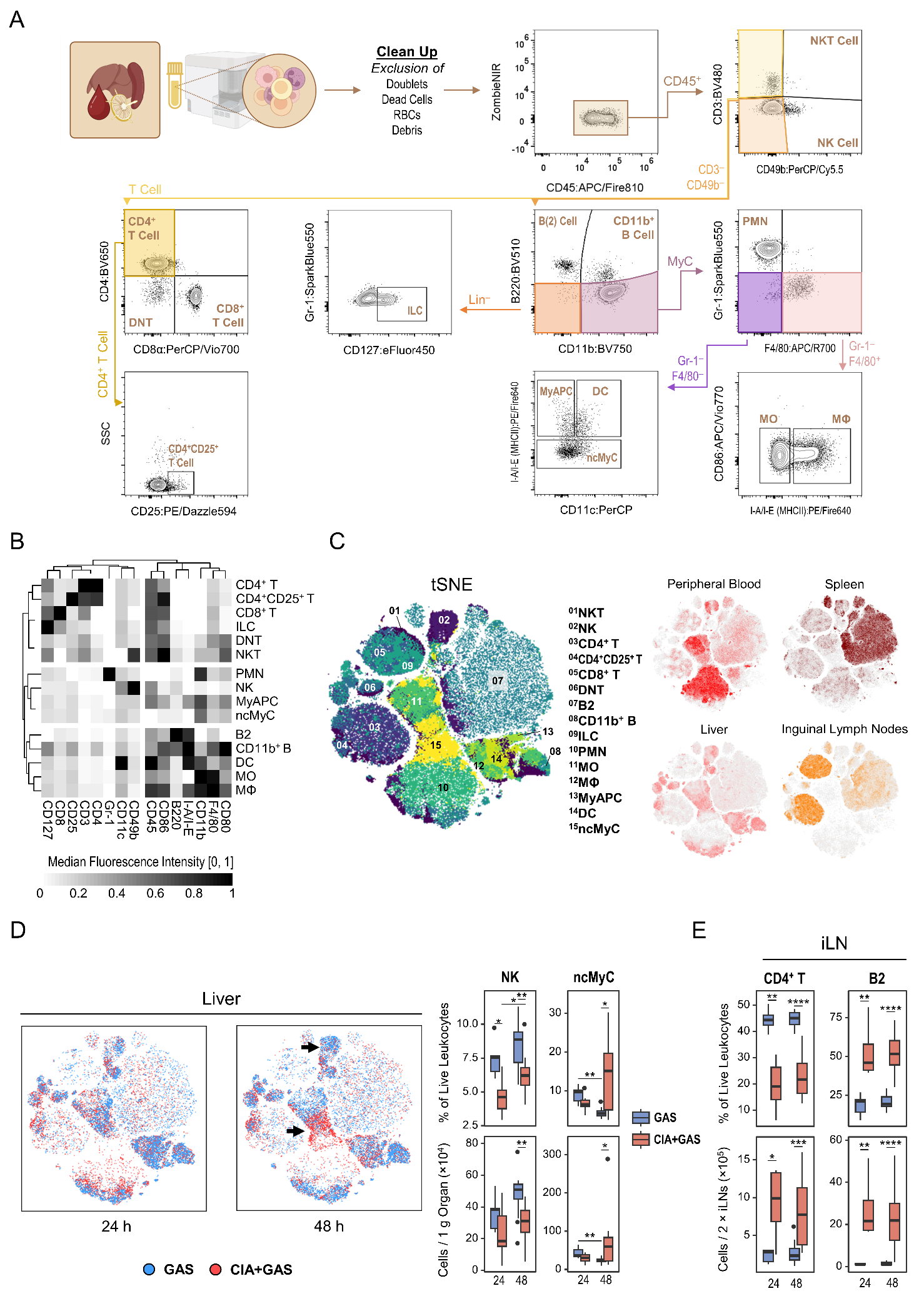


Supplementary Figure S2 (on previous page). Sepsis-induced alterations to the peripheral immune landscape. (A) Gating strategy for flow cytometry analyses of peripheral blood, spleen, liver, and inguinal lymph nodes (iLN). (B) Grayscale heatmap depicting the expression of surface antigens in manually gated immune cell populations, based on median fluorescence intensities normalized to a 0–1 range. (C) Topological maps generated using t-distributed stochastic neighbor embedding (tSNE) illustrate immune cells identified by flow cytometry and hierarchical gating. Immune cell populations are indicated by color coding and numbered annotations. Immune cell topology maps of peripheral blood (red), spleen (brown), liver (pink), and inguinal lymph nodes (iLN, orange) are shown. (D) Superimposed topological tSNE maps illustrating liver immune cell distributions in mice without underlying conditions or CIA infected for 24 hours (left) and 48 hours (right), respectively. Box plots showing frequencies and cell counts of natural killer (NK) cells and non-classical myeloid cells (ncMyC) in the liver from mice without underlying conditions infected for 24 hours (GAS, n = 5) or 48 hours (GAS, n = 14), and mice with pre-existing collagen-induced arthritis infected for 24 hours (CIA+GAS, n = 7) or 48 hours (CIA+GAS, n = 14). (E) Box plots displaying frequencies and cell counts of CD4^+^ T cells, and B2 cells. Box plots indicate the median, with the lower and upper hinges corresponding to the 25^th^ and 75^th^ percentiles. Whiskers extend to the smallest and largest values within 1.5 × interquartile range IQR). p-values were obtained using Dunn’s test (D). p < 0.05 (*), p < 0.01 (**), p < 0.001 (***), p < 0.0001 (****).


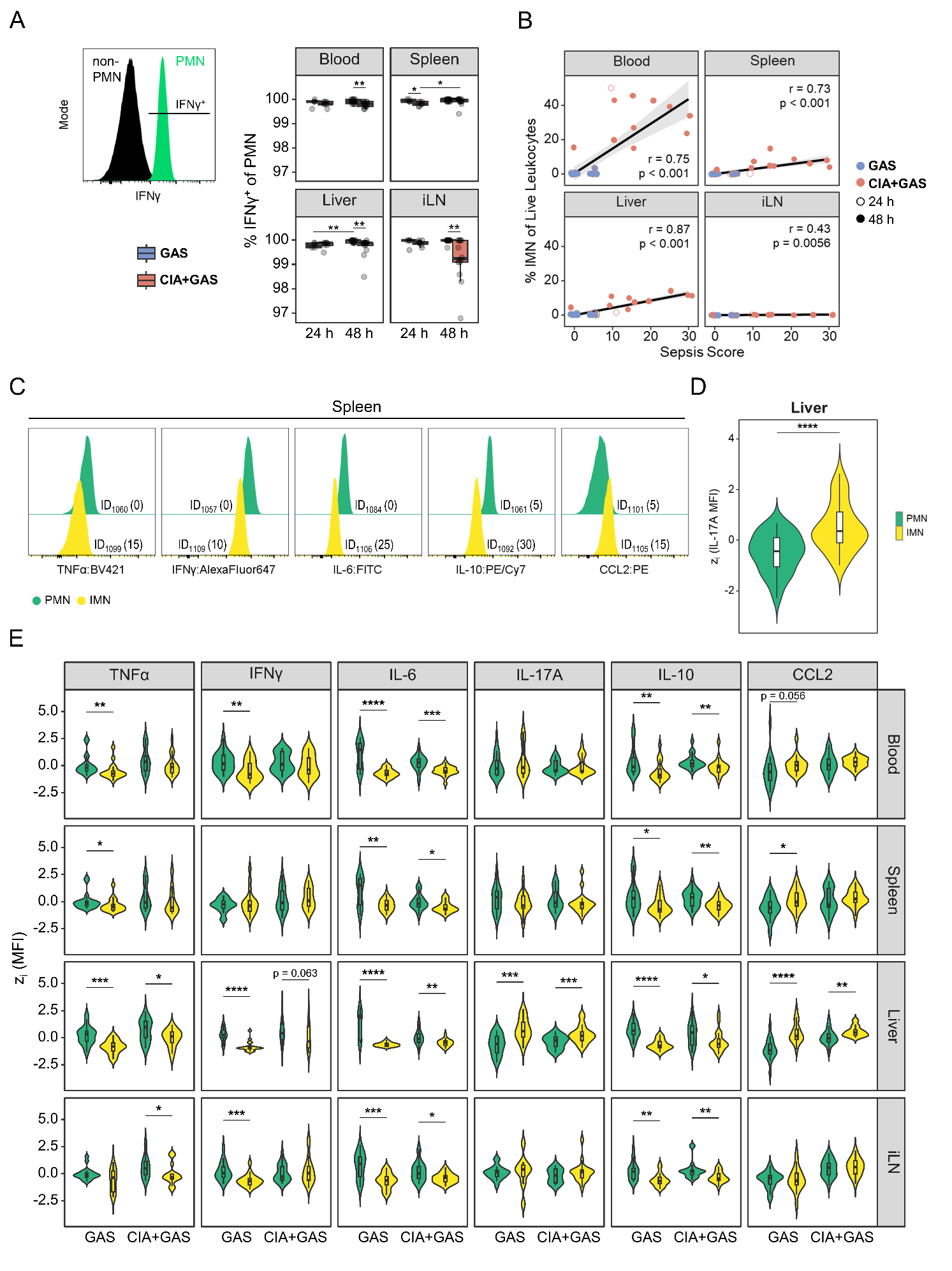


Supplementary Figure S3 (on previous page). **Effector functions of polymorphonuclear neutrophils (PMN) compared to immature neutrophils (IMN).** (A) Histogram (left) showing interferon (IFN)γ expression among PMN and non-PMN. Box plots (right) display the frequency of IFNγ-producing PMN in peripheral blood (PB), spleen, liver, and inguinal lymph nodes (iLN). Data were generated from mice without underlying conditions infected for 24 hours (GAS, n = 5) or 48 hours (GAS, n = 14), and mice with pre-existing CIA infected for 24 hours (CIA+GAS, n = 7) or 48 hours (CIA+GAS, n = 14). (B) Scatter plots depicting the relationship between IMN frequencies and sepsis scores across different organs. Each dot represents one animal. The black regression line follows a linear model with locally estimated scatterplot smoothing (LOESS), and the gray area represents the 95% confidence interval. (C) Example histograms showing cytokine expressions in spleen PMNs and IMNs. Numbers in parentheses indicate the sepsis score of the respective mouse. (D) Violin and superimposed box plots illustrating interleukin (IL-)17A expression in PMN (n = 40) compared to IMN (n = 40) in the liver, based on standardized (z) median fluorescence intensity (MFI) values. (E) Violin and superimposed box plots depicting for PMN and IMN expression levels of tumor necrosis factor (TNF)α, IFNγ, IL-6, IL-17A, IL-10, and C-C motif chemokine ligand (CCL)2 in different organs. Box plots indicate the median, with the lower and upper hinges corresponding to the 25^th^ and 75^th^ percentiles. Whiskers extend to the smallest and largest values within 1.5 × interquartile range (IQR). For panels (D) and (E), data were acquired from cells isolated from mice infected for 24 or 48 hours.
r: Pearson correlation coefficient. p-values were obtained using Dunn’s test (A, D), Pearson correlation analysis (B), or the Mann-Whitney U test (C). p < 0.05 (*), p < 0.01 (**), p < 0.001 (***), p < 0.0001 (****).


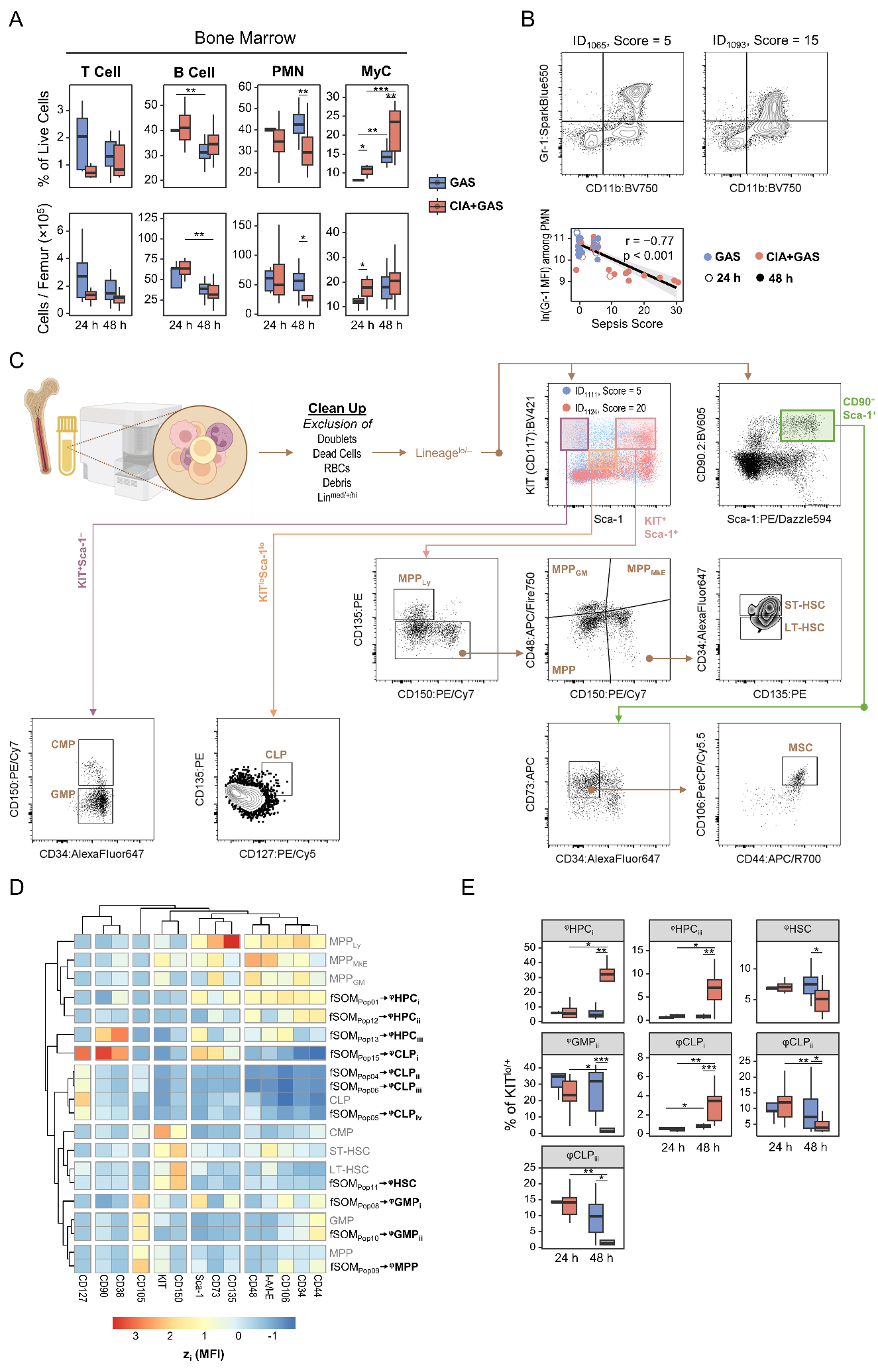


Supplementary Figure S4 (on previous page). **Alterations in the medullary immune, progenitor, and stem cell compartments following sepsis.** (A) Box plots showing the frequencies and cell counts of T and B lymphocytes, polymorphonuclear neutrophils (PMN), and myeloid cells (MyC) in mice without underlying conditions infected for 24 hours (GAS, n = 5) or 48 hours (GAS, n = 17), and mice with pre-existing collagen-induced arthritis infected for 24 hours (CIA+GAS, n = 7) or 48 hours (CIA+GAS, n = 15). (B) Representative contour plots (top) illustrating Gr-1 expression among medullary CD3⁻B220⁻ cells in animals with different sepsis scores. Scatter plot (bottom) depicting Gr-1 levels in relation to sepsis scores. Each dot represents one animal. The black regression line follows a linear model with locally estimated scatterplot smoothing (LOESS), and the gray area represents the 95% confidence interval. (C) Gating strategy for flow cytometry analyses of medullary stem and progenitor cells. MPP: Multipotent progenitor. ST-/LT-HSC: Short-term/long-term hematopoietic stem cell. CMP: Common myeloid progenitor. GMP: Granulocyte-monocyte progenitor. CLP: Common lymphoid progenitor. MSC: Mesenchymal progenitor cell. (D) Heatmap of standardized (z) expression values of stem cell markers, based on median fluorescence intensities (MFI) and hierarchical clustering of bone marrow stem and progenitor cells. Cell populations were identified either by manual gating (as shown in C) or by flow Self-Organizing Map (fSOM). HPC: Hematopoietic progenitor cell. (E) Box plots showing frequencies of FlowSOM-generated stem and progenitor cells in the bone marrow. Box plots indicate the median, with the lower and upper hinges corresponding to the 25^th^ and 75^th^ percentiles. Whiskers extend to the smallest and largest values within 1.5 × interquartile range (IQR). r: Pearson correlation coefficient. p-values were obtained using Dunn’s test (A, E), or Pearson correlation analysis (B). p < 0.05 (*), p < 0.01 (**), p < 0.001 (***).


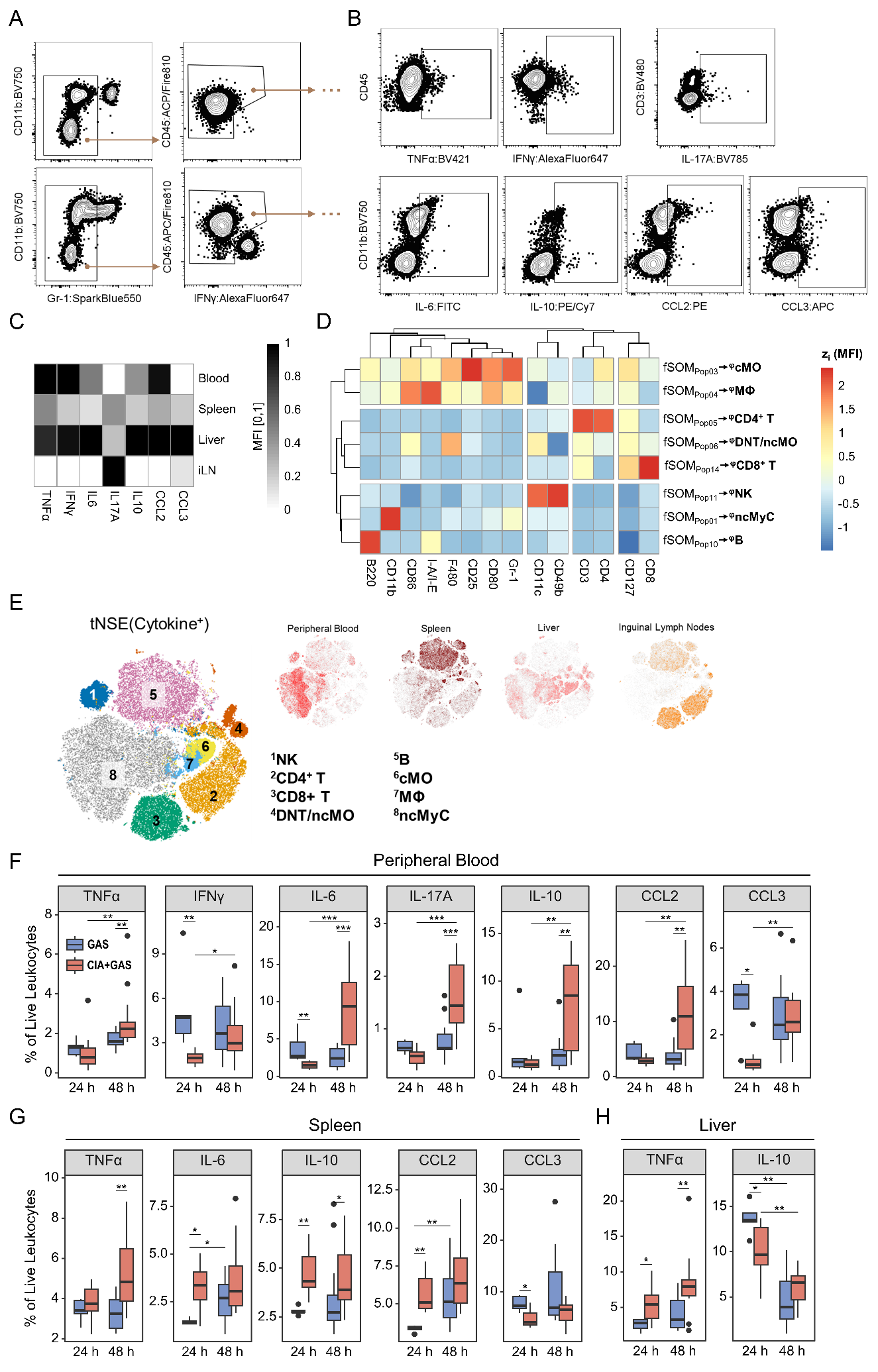


Supplementary Figure S5 (on previous page). **Cellular cytokine production in peripheral organs during sepsis.** (A) Gating strategy for the exclusion of polymorphonuclear and immature neutrophils in samples from animals with low (top, representative contour plot) and high sepsis scores (bottom). (B) Gating strategy for identifying cells that produce tumor necrosis factor (TNF)α, interferon (IFN)γ, interleukin (IL-)6, IL-17A, IL-10, C-C motif chemokine ligand (CCL)2, or CCL3. (C) Grayscale heatmap showing intracellular cytokine expression in peripheral blood, spleen, liver, and inguinal lymph nodes (iLN), based on median fluorescence intensities (MFI) normalized to a 0–1 range. (D) Heatmap of standardized (z) expression values of immune cell lineage markers, based on median fluorescence intensities (MFI) for cytokine-producing populations clustered by flow Self-Organizing Map (fSOM). (E) Topological maps generated using t-distributed stochastic neighbor embedding (tSNE) illustrate cytokine-producing immune cells identified by flow cytometry and flow self-organizing map (FlowSOM). Cell populations expressing cytokines are indicated by color coding and numbered annotations. Immune cell topology maps of peripheral blood (red), spleen (brown), liver (pink), and inguinal lymph nodes (iLN, orange) are shown. (F–H) Box plots illustrating the frequencies of cytokine-expressing cells in peripheral blood (F), spleen (G), and liver (H) obtained from mice without underlying conditions infected for 24 hours (GAS, n = 5) or 48 hours (GAS, n = 17), and mice with pre-existing collagen-induced arthritis infected for 24 hours (CIA+GAS, n = 7) or 48 hours (CIA+GAS, n = 15). Box plots indicate the median, with the lower and upper hinges corresponding to the 25^th^ and 75^th^ percentiles. Whiskers extend to the smallest and largest values within 1.5 × interquartile range (IQR). p-values were obtained using Dunn’s test. p < 0.05 (*), p < 0.01 (**), p < 0.001 (***).


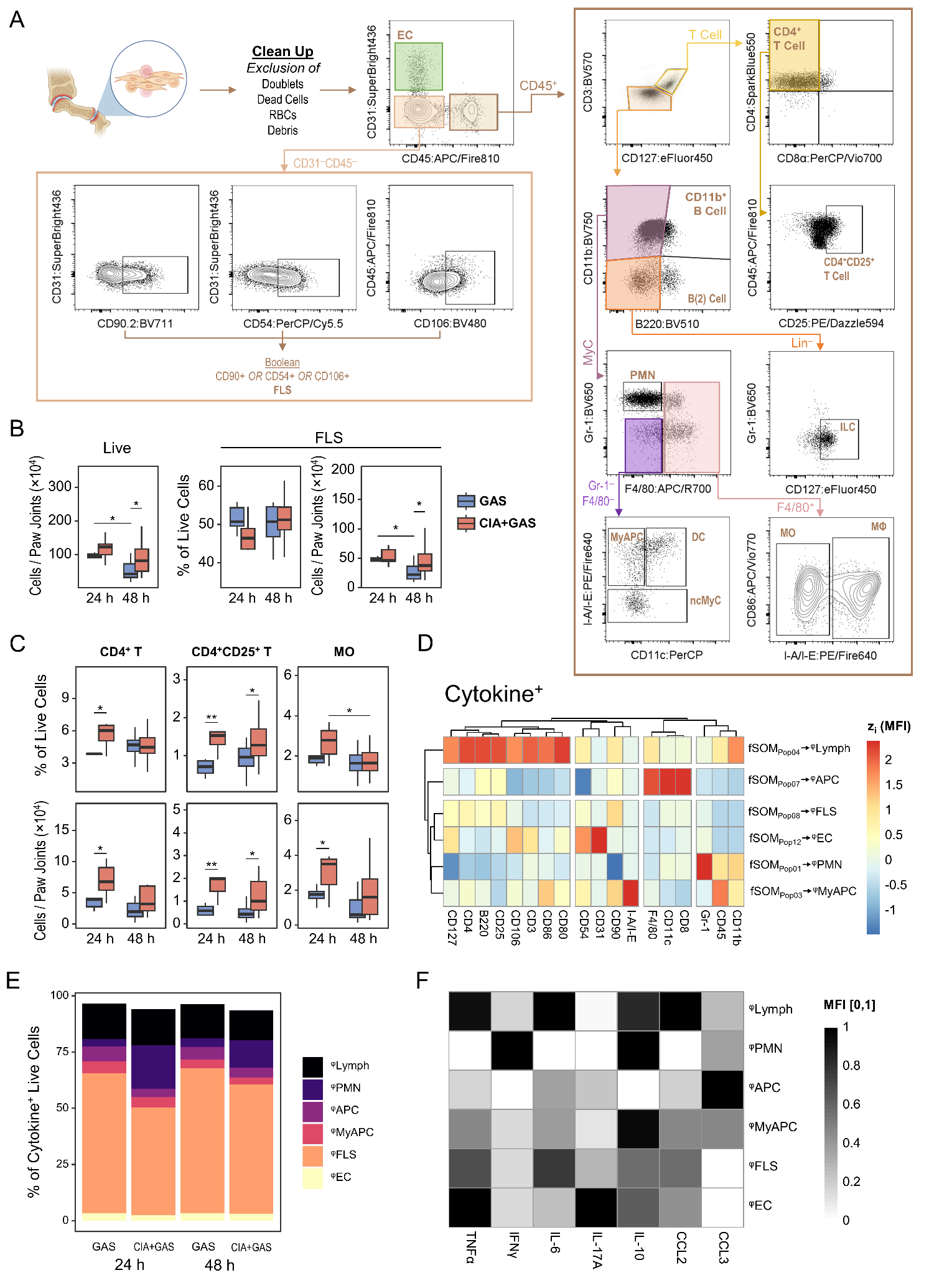


Supplementary Figure S6 (on previous page). Cellular composition and cytokine production in paw joints. (A) Gating strategy for flow cytometry analyses of paw joint extracts. (B) Box plots depicting absolute cell counts (left) and the frequencies as well as cell counts of fibroblast-like synoviocytes (FLS, right) in mice without underlying conditions infected for 24 hours (GAS, n = 5) or 48 hours (GAS, n = 17), and mice with pre-existing collagen-induced arthritis infected for 24 hours (CIA+GAS, n = 7) or 48 hours (CIA+GAS, n = 15). (C) Box plots presenting the frequencies and cell counts of CD4^+^ T lymphocytes, CD4^+^CD25^+^ T lymphocytes, and monocytes (MO). Box plots indicate the median, with the lower and upper hinges corresponding to the 25^th^ and 75^th^ percentiles. Whiskers extend to the smallest and largest values within 1.5 × interquartile range (IQR).
(D) Heatmap of standardized (z) expression values of surface markers, based on median fluorescence intensities (MFI) for cytokine-producing populations clustered by flow Self-Organizing Map (fSOM). (E) Stacked bar plot showing the composition of cytokine-producing cells in paw joints. (F) Grayscale heatmap illustrating intracellular expression of tumor necrosis factor (TNF)α, interferon (IFN)γ, interleukin (IL-)6, IL-17A, IL-10, CC-motif chemokine ligand (CCL)2, or CCL3 in fSOM-generated populations, based on median fluorescence intensities (MFI) normalized to a 0–1 range. p-values were obtained using Dunn’s test. p < 0.05 (*), p < 0.01 (**).

# Supplementary Tables

Supplementary Table S1. Isolation of β-hemolytic bacteria from tibiofemoral joint cavities.

| Positive for β-Hemolytic Bacteria | 24 h | | p | 48 h | | p |
| --- | --- | --- | --- | --- | --- | --- |
|  | GAS | CIA+GAS |  | GAS | CIA+GAS |  |
|  |  |  |  |  |  |  |
| Left Knee | 0/5 | 0/7 | 1 | 1/13 | 2/15 | 0.43 |
| Right Knee | 0/5 | 3/7 |  | 0/13 | 4/15 |  |

Supplementary Table S2. Summary and statistics on frequency and cell count data from manually gated immune cell populations in peripheral blood, spleen, liver and inguinal lymph nodes (iLN).

| *Population* | 24 h |  |  |  |  |  |  |  | 48 h |  |  |  |  |  |  |  | p_Dunn_ |  |  |  |
| --- | --- | --- | --- | --- | --- | --- | --- | --- | --- | --- | --- | --- | --- | --- | --- | --- | --- | --- | --- | --- |
| **Blood** | GAS |  |  |  | CIA+GAS |  |  |  | GAS |  |  |  | CIA+GAS |  |  |  | Group |  | Time |  |
| % | Min | **Median** | Max | MAD | Min | **Median** | Max | MAD | Min | **Median** | Max | MAD | Min | **Median** | Max | MAD | 24 h | 48 h | GAS | CIA+GAS |
| NKT | 0.05 | **0.15** | 0.18 | 0.04 | 0.04 | **0.07** | 0.20 | 0.04 | 0.05 | **0.11** | 0.54 | 0.05 | 0.06 | **0.13** | 0.24 | 0.10 | 0.416 | 0.818 | 0.816 | 0.155 |
| NK | 1.77 | **3.61** | 4.61 | 1.48 | 0.78 | **2.35** | 5.09 | 1.19 | 3.21 | **5.02** | 8.03 | 1.36 | 1.98 | **3.60** | 5.23 | 0.89 | 0.685 | 0.008 | 0.042 | 0.101 |
| CD4^+^ T | 3.09 | **4.16** | 6.11 | 0.64 | 0.82 | **1.65** | 4.78 | 1.23 | 2.68 | **6.86** | 13.20 | 2.56 | 0.46 | **1.25** | 7.99 | 1.01 | 0.223 | 0.001 | 0.064 | 0.881 |
| CD4^+^CD25^+^ T | 0.17 | **0.26** | 0.29 | 0.04 | 0.06 | **0.11** | 0.24 | 0.06 | 0.09 | **0.20** | 0.40 | 0.07 | 0.04 | **0.15** | 0.88 | 0.12 | 0.019 | 0.421 | 0.150 | 0.411 |
| CD8^+^ T | 1.40 | **2.39** | 3.26 | 0.27 | 0.19 | **0.87** | 2.47 | 1.01 | 1.59 | **3.65** | 7.45 | 1.83 | 0.06 | **0.39** | 4.45 | 0.41 | 0.122 | 0.001 | 0.064 | 0.576 |
| DNT | 0.15 | **0.23** | 0.41 | 0.10 | 0.18 | **0.27** | 0.56 | 0.13 | 0.21 | **0.33** | 1.55 | 0.07 | 0.29 | **1.49** | 9.18 | 0.99 | 0.372 | 0.001 | 0.178 | 0.006 |
| B2 | 6.84 | **30.10** | 34.30 | 4.74 | 3.51 | **10.70** | 55.70 | 8.42 | 6.59 | **21.80** | 41.40 | 14.08 | 1.06 | **14.55** | 45.90 | 18.30 | 0.123 | 0.154 | 0.610 | 0.765 |
| CD11b^+^ B | 0.23 | **0.30** | 0.60 | 0.10 | 0.07 | **0.23** | 0.51 | 0.07 | 0.09 | **0.27** | 0.75 | 0.16 | 0.19 | **0.75** | 5.30 | 0.52 | 0.122 | 0.005 | 0.459 | 0.005 |
| ILC | 0.46 | **0.60** | 0.84 | 0.12 | 0.08 | **0.23** | 0.38 | 0.10 | 0.30 | **1.11** | 2.83 | 0.59 | 0.07 | **0.26** | 1.46 | 0.23 | 0.004 | 0.001 | 0.037 | 0.349 |
| PMN | 33.50 | **39.30** | 69.40 | 0.59 | 16.50 | **71.90** | 83.60 | 16.46 | 18.80 | **37.30** | 66.80 | 14.16 | 6.87 | **23.05** | 43.10 | 6.23 | 0.088 | 0.022 | 0.578 | 0.006 |
| MO | 6.93 | **9.75** | 11.00 | 1.85 | 1.94 | **4.14** | 8.16 | 2.03 | 3.52 | **13.70** | 23.80 | 6.69 | 5.31 | **13.75** | 28.60 | 4.91 | 0.007 | 0.520 | 0.138 | <0.001 |
| MΦ | 1.84 | **2.66** | 4.68 | 0.82 | 0.26 | **0.60** | 3.12 | 0.49 | 0.87 | **1.35** | 6.41 | 0.24 | 1.21 | **3.12** | 6.06 | 0.76 | 0.028 | 0.059 | 0.126 | 0.002 |
| MyAPC | 0.43 | **0.66** | 1.23 | 0.34 | 0.11 | **0.20** | 2.42 | 0.04 | 0.14 | **0.34** | 1.99 | 0.23 | 0.14 | **0.81** | 4.46 | 0.76 | 0.042 | 0.103 | 0.033 | 0.126 |
| DC | 0.28 | **0.36** | 0.62 | 0.12 | 0.02 | **0.04** | 0.61 | 0.03 | 0.07 | **0.12** | 0.96 | 0.07 | 0.07 | **0.29** | 1.24 | 0.21 | 0.028 | 0.023 | 0.018 | 0.011 |
| ncMyC | 2.29 | **2.40** | 4.83 | 0.16 | 0.57 | **1.19** | 32.30 | 0.92 | 0.76 | **1.69** | 3.53 | 1.12 | 0.73 | **25.30** | 47.10 | 25.83 | 0.167 | 0.005 | 0.115 | 0.044 |
| Count × 10^4^ |  |  |  |  |  |  |  |  |  |  |  |  |  |  |  |  |  |  |  |  |
| NKT | 21 | **38** | 45 | 11 | 5 | **11** | 48 | 8 | 16 | **46** | 194 | 35 | 1 | **14** | 41 | 13 | 0.088 | <0.001 | 0.267 | 0.602 |
| NK | 708 | **903** | 1245 | 115 | 70 | **357** | 1527 | 285 | 729 | **2082** | 4898 | 601 | 22 | **370** | 2505 | 289 | 0.167 | <0.001 | 0.005 | 0.823 |
| CD4^+^ T | 834 | **1528** | 1664 | 202 | 52 | **391** | 1147 | 397 | 668 | **3137** | 4765 | 1491 | 17 | **156** | 3823 | 173 | 0.019 | <0.001 | 0.016 | 0.296 |
| CD4^+^CD25^+^ T | 46 | **73** | 112 | 39 | 5 | **19** | 58 | 21 | 15 | **87** | 159 | 41 | 1 | **18** | 214 | 23 | 0.012 | 0.026 | 0.517 | 0.941 |
| CD8^+^ T | 378 | **815** | 956 | 209 | 13 | **222** | 557 | 287 | 316 | **1543** | 3182 | 1052 | 4 | **30** | 2781 | 33 | 0.012 | 0.001 | 0.016 | 0.332 |
| DNT | 55 | **63** | 164 | 12 | 34 | **43** | 130 | 11 | 51 | **169** | 558 | 84 | 13 | **142** | 1561 | 75 | 0.104 | 0.872 | 0.052 | 0.006 |
| B2 | 1847 | **7525** | 13720 | 8418 | 211 | **1368** | 16710 | 1620 | 1120 | **8895** | 24381 | 6274 | 64 | **803** | 30222 | 978 | 0.088 | 0.012 | 0.643 | 0.456 |
| CD11b^+^ B | 58 | **96** | 252 | 22 | 6 | **43** | 72 | 31 | 33 | **128** | 260 | 51 | 21 | **108** | 336 | 75 | 0.015 | 0.462 | 0.405 | 0.021 |
| ILC | 140 | **170** | 353 | 30 | 5 | **40** | 111 | 29 | 108 | **596** | 1147 | 350 | 1 | **27** | 672 | 35 | 0.004 | <0.001 | 0.042 | 0.881 |
| PMN | 9725 | **13400** | 18738 | 5300 | 3330 | **12450** | 20900 | 8864 | 6762 | **17033** | 35640 | 7541 | 129 | **2982** | 16560 | 2641 | 0.570 | <0.001 | 0.579 | 0.011 |
| MO | 1871 | **2438** | 4620 | 840 | 116 | **791** | 2448 | 789 | 1162 | **6618** | 8479 | 2649 | 88 | **1964** | 9522 | 1291 | 0.019 | 0.002 | 0.064 | 0.073 |
| MΦ | 497 | **743** | 1872 | 213 | 16 | **110** | 936 | 128 | 278 | **668** | 2098 | 396 | 29 | **306** | 1408 | 244 | 0.028 | 0.017 | 0.711 | 0.052 |
| MyAPC | 158 | **181** | 436 | 34 | 10 | **41** | 726 | 14 | 49 | **114** | 856 | 63 | 6 | **74** | 291 | 55 | 0.042 | 0.057 | 0.126 | 0.205 |
| DC | 76 | **134** | 208 | 66 | 1 | **10** | 183 | 11 | 23 | **47** | 413 | 32 | 4 | **50** | 149 | 44 | 0.028 | 0.280 | 0.116 | 0.101 |
| ncMyC | 600 | **1004** | 1208 | 302 | 88 | **286** | 1938 | 293 | 266 | **712** | 1320 | 356 | 190 | **1973** | 8007 | 2071 | 0.088 | 0.148 | 0.405 | 0.021 |

Supplementary Table S2 (continued).

| **Spleen** | 24 h |  |  |  |  |  |  |  | 48 h |  |  |  |  |  |  |  | p_Dunn_ |  |  |  |
| --- | --- | --- | --- | --- | --- | --- | --- | --- | --- | --- | --- | --- | --- | --- | --- | --- | --- | --- | --- | --- |
|  | GAS |  |  |  | CIA+GAS |  |  |  | GAS |  |  |  | CIA+GAS |  |  |  | Group |  | Time |  |
| % | Min | **Median** | Max | MAD | Min | Median | Max | MAD | Min | **Median** | Max | MAD | Min | **Median** | Max | MAD | 24 h | 48 h | GAS | CIA+GAS |
| NKT | 0.16 | **0.25** | 0.35 | 0.06 | 0.04 | 0.06 | 0.27 | 0.04 | 0.05 | **0.12** | 0.20 | 0.04 | 0.02 | **0.09** | 0.11 | 0.03 | 0.028 | 0.012 | 0.002 | 0.851 |
| NK | 3.05 | **4.14** | 7.80 | 1.62 | 0.74 | 2.43 | 7.72 | 1.11 | 3.46 | **4.87** | 7.41 | 1.31 | 1.36 | **3.17** | 5.48 | 0.68 | 0.088 | <0.001 | 0.926 | 0.502 |
| CD4^+^ T | 10.20 | **11.50** | 12.10 | 0.44 | 2.62 | 5.44 | 8.74 | 3.84 | 5.61 | **8.36** | 10.20 | 0.90 | 3.02 | **4.37** | 10.70 | 1.71 | 0.004 | 0.009 | 0.001 | 0.765 |
| CD4^+^CD25^+^ T | 0.43 | **0.48** | 0.63 | 0.07 | 0.35 | 0.57 | 0.76 | 0.21 | 0.40 | **0.46** | 0.82 | 0.07 | 0.34 | **0.71** | 0.84 | 0.09 | 0.684 | 0.005 | 0.780 | 0.108 |
| CD8^+^ T | 7.02 | **8.27** | 8.77 | 0.74 | 0.76 | 3.04 | 6.05 | 2.83 | 4.70 | **6.21** | 7.76 | 0.90 | 0.38 | **1.90** | 8.19 | 1.70 | 0.004 | 0.004 | 0.002 | 0.823 |
| DNT | 0.53 | **0.80** | 0.94 | 0.12 | 0.79 | 0.92 | 1.51 | 0.18 | 0.56 | **0.74** | 1.00 | 0.07 | 0.63 | **1.32** | 4.37 | 0.44 | 0.142 | <0.001 | 0.610 | 0.145 |
| B2 | 51.40 | **60.10** | 62.30 | 2.52 | 52.40 | 67.60 | 82.80 | 7.41 | 54.30 | **60.70** | 66.70 | 4.97 | 56.30 | **69.15** | 79.10 | 7.19 | 0.123 | 0.004 | 0.354 | 0.681 |
| CD11b^+^ B | 0.26 | **0.29** | 0.38 | 0.04 | 0.31 | 0.36 | 0.87 | 0.03 | 0.23 | **0.47** | 0.76 | 0.15 | 0.31 | **0.54** | 1.14 | 0.23 | 0.050 | 0.346 | 0.041 | 0.135 |
| ILC | 0.26 | **0.40** | 0.61 | 0.21 | 0.40 | 0.77 | 0.86 | 0.10 | 0.63 | **1.16** | 3.27 | 0.65 | 0.34 | **0.71** | 1.49 | 0.26 | 0.041 | 0.005 | 0.001 | 0.881 |
| PMN | 5.49 | **7.46** | 12.00 | 2.46 | 1.11 | 8.38 | 28.10 | 9.52 | 2.86 | **6.56** | 21.60 | 4.01 | 1.14 | **4.39** | 15.40 | 2.31 | 0.570 | 0.060 | 0.643 | 0.086 |
| MO | 2.15 | **2.52** | 2.71 | 0.21 | 0.78 | 2.09 | 4.02 | 1.04 | 1.76 | **3.88** | 6.74 | 1.12 | 0.53 | **2.60** | 6.31 | 1.75 | 0.291 | 0.035 | 0.079 | 0.502 |
| MΦ | 0.98 | **1.76** | 2.14 | 0.42 | 0.89 | 1.94 | 2.52 | 0.74 | 0.76 | **1.31** | 3.82 | 0.77 | 0.64 | **1.63** | 3.65 | 0.57 | 0.808 | 0.613 | 0.711 | 0.765 |
| MyAPC | 0.28 | **0.47** | 0.52 | 0.07 | 0.07 | 0.32 | 0.99 | 0.31 | 0.07 | **0.16** | 0.50 | 0.10 | 0.09 | **0.16** | 0.76 | 0.07 | 0.122 | 0.645 | 0.012 | 0.765 |
| DC | 0.16 | **0.17** | 0.21 | 0.01 | 0.03 | 0.11 | 0.22 | 0.04 | 0.05 | **0.10** | 0.29 | 0.04 | 0.07 | **0.15** | 0.37 | 0.06 | 0.042 | 0.062 | 0.046 | 0.091 |
| ncMyC | 0.75 | **0.85** | 1.20 | 0.15 | 0 | 1 | 1 | 0 | 0.27 | **0.46** | 0.76 | 0.19 | 0.36 | **4.55** | 14.90 | 3.60 | 0.061 | 0.001 | 0.002 | 0.014 |
| Count × 10^4^ |  |  |  |  |  |  |  |  |  |  |  |  |  |  |  |  |  |  |  |  |
| NKT | 525 | **730** | 1526 | 248 | 107 | 179 | 1328 | 107 | 234 | **572** | 944 | 182 | 43 | **262** | 537 | 168 | 0.028 | 0.002 | 0.165 | 0.502 |
| NK | 10004 | **18050** | 22776 | 7006 | 2309 | 5916 | 37982 | 2594 | 9853 | **22476** | 35864 | 6055 | 3917 | **9803** | 18413 | 6755 | 0.088 | <0.001 | 0.079 | 0.179 |
| CD4^+^ T | 30192 | **33580** | 51448 | 5023 | 6078 | 18951 | 25896 | 8186 | 15456 | **38586** | 60384 | 17459 | 7973 | **16226** | 55212 | 9816 | 0.004 | 0.006 | 1.000 | 0.941 |
| CD4^+^CD25^+^ T | 1286 | **1480** | 1875 | 287 | 812 | 1885 | 2017 | 196 | 986 | **2296** | 3808 | 925 | 1142 | **2527** | 3922 | 963 | 0.465 | 0.854 | 0.042 | 0.062 |
| CD8^+^ T | 21786 | **23026** | 38237 | 1838 | 1763 | 14278 | 16156 | 2784 | 10640 | **30259** | 46250 | 11612 | 1003 | **7159** | 42260 | 6949 | 0.004 | 0.002 | 0.643 | 0.881 |
| DNT | 1420 | **2336** | 3619 | 1108 | 1856 | 2870 | 4231 | 1296 | 1411 | **3612** | 5624 | 1561 | 2394 | **4279** | 11537 | 1679 | 0.372 | 0.168 | 0.116 | 0.015 |
| B2 | 150088 | **178192** | 254624 | 28075 | 121568 | 189280 | 341448 | 52496 | 133056 | **278848** | 461760 | 128278 | 146232 | **239124** | 360680 | 83037 | 0.935 | 0.232 | 0.042 | 0.156 |
| CD11b^+^ B | 697 | **993** | 1264 | 208 | 732 | 1092 | 2018 | 534 | 580 | **1990** | 5624 | 1179 | 893 | **2116** | 3967 | 829 | 0.291 | 0.854 | 0.016 | 0.033 |
| ILC | 759 | **1744** | 1837 | 138 | 944 | 2083 | 2683 | 141 | 1411 | **5279** | 21844 | 3213 | 966 | **2560** | 4291 | 1020 | 0.028 | 0.001 | 0.003 | 0.101 |
| PMN | 15544 | **24469** | 35040 | 12462 | 2753 | 23075 | 65192 | 27228 | 7207 | **33131** | 90944 | 21426 | 3922 | **14050** | 51744 | 10123 | 0.570 | 0.012 | 0.267 | 0.179 |
| MO | 6278 | **7074** | 11598 | 1181 | 1934 | 5852 | 13727 | 3218 | 3942 | **18283** | 34114 | 12524 | 1823 | **8882** | 21202 | 7102 | 0.223 | 0.008 | 0.016 | 0.233 |
| MΦ | 3966 | **5139** | 6334 | 1377 | 2406 | 4802 | 9545 | 3003 | 2369 | **6532** | 18336 | 3217 | 3302 | **5404** | 9636 | 2709 | 0.808 | 0.312 | 0.267 | 0.412 |
| MyAPC | 992 | **1421** | 1542 | 179 | 203 | 742 | 4871 | 644 | 234 | **737** | 2360 | 382 | 259 | **563** | 2554 | 300 | 0.042 | 0.383 | 0.096 | 0.765 |
| DC | 429 | **555** | 741 | 99 | 81 | 260 | 1082 | 72 | 159 | **505** | 1369 | 345 | 290 | **474** | 1243 | 192 | 0.042 | 0.748 | 0.643 | 0.009 |
| ncMyC | 2171 | **2788** | 5232 | 887 | 614 | 1290 | 4428 | 210 | 605 | **2175** | 5077 | 1334 | 1824 | **12744** | 39336 | 13660 | 0.028 | 0.001 | 0.229 | 0.001 |

Supplementary Table S2 (continued).

| **Liver** | 24 h |  |  |  |  |  |  |  | 48 h |  |  |  |  |  |  |  | p_Dunn_ |  |  |  |
| --- | --- | --- | --- | --- | --- | --- | --- | --- | --- | --- | --- | --- | --- | --- | --- | --- | --- | --- | --- | --- |
|  | GAS |  |  |  | CIA+GAS |  |  |  | GAS |  |  |  | CIA+GAS |  |  |  | Group |  | Time |  |
| % | Min | **Median** | Max | MAD | Min | **Median** | Max | MAD | Min | **Median** | Max | MAD | Min | **Median** | Max | MAD | 24 h | 48 h | GAS | CIA+GAS |
| NKT | 0.17 | **0.40** | 0.57 | 0.13 | 0.23 | **0.40** | 0.64 | 0.16 | 0.26 | **0.48** | 2.13 | 0.28 | 0.12 | **0.52** | 1.31 | 0.38 | 0.744 | 0.713 | 0.379 | 0.279 |
| NK | 5.98 | **7.54** | 9.68 | 1.53 | 2.93 | **4.60** | 6.87 | 1.26 | 5.53 | **8.86** | 11.30 | 1.41 | 4.07 | **6.21** | 9.94 | 1.04 | 0.012 | 0.002 | 0.229 | 0.044 |
| CD4^+^ T | 3.79 | **4.12** | 8.22 | 0.49 | 2.72 | **5.39** | 13.20 | 2.42 | 4.37 | **5.84** | 15.80 | 1.45 | 5.28 | **8.91** | 12.70 | 3.16 | 0.167 | 0.035 | 0.042 | 0.179 |
| CD4^+^CD25^+^ T | 0.35 | **0.49** | 0.84 | 0.16 | 0.35 | **0.98** | 3.95 | 0.22 | 0.20 | **0.63** | 1.45 | 0.47 | 0.15 | **1.75** | 3.13 | 1.00 | 0.051 | 0.008 | 0.308 | 0.296 |
| CD8^+^ T | 1.39 | **2.08** | 3.47 | 1.02 | 0.67 | **1.58** | 3.67 | 0.28 | 1.09 | **2.65** | 8.55 | 0.52 | 0.74 | **2.24** | 4.25 | 1.10 | 0.167 | 0.098 | 0.459 | 0.156 |
| DNT | 2.70 | **2.93** | 3.99 | 0.34 | 3.41 | **4.04** | 16.00 | 0.71 | 1.34 | **4.14** | 6.17 | 1.31 | 1.23 | **8.30** | 19.90 | 0.93 | 0.042 | 0.001 | 0.195 | 0.044 |
| B2 | 11.50 | **20.10** | 21.30 | 1.78 | 6.15 | **7.90** | 34.20 | 2.43 | 6.52 | **20.55** | 33.40 | 9.56 | 5.43 | **9.40** | 31.60 | 5.54 | 0.042 | 0.037 | 0.643 | 0.794 |
| CD11b^+^ B | 3.38 | **4.15** | 6.15 | 1.14 | 1.42 | **3.83** | 4.86 | 1.53 | 2.22 | **4.49** | 7.69 | 1.53 | 0.83 | **2.17** | 9.72 | 1.02 | 0.223 | 0.013 | 0.711 | 0.551 |
| ILC | 0.65 | **0.94** | 1.26 | 0.31 | 1.11 | **1.49** | 2.89 | 0.36 | 0.75 | **1.49** | 2.18 | 0.47 | 0.60 | **1.84** | 3.38 | 0.94 | 0.018 | 0.168 | 0.009 | 0.601 |
| PMN | 5.68 | **6.42** | 24.20 | 1.10 | 4.56 | **19.30** | 26.00 | 7.86 | 5.47 | **11.40** | 30.40 | 5.21 | 3.51 | **8.27** | 17.40 | 3.11 | 0.223 | 0.098 | 0.195 | 0.030 |
| MO | 3.97 | **5.65** | 7.42 | 0.93 | 4.69 | **8.43** | 13.30 | 5.00 | 3.80 | **7.70** | 19.60 | 2.63 | 2.27 | **6.67** | 11.90 | 2.29 | 0.062 | 0.118 | 0.042 | 0.117 |
| MΦ | 7.62 | **10.00** | 11.40 | 1.33 | 5.27 | **9.37** | 12.40 | 3.60 | 4.17 | **6.80** | 12.40 | 2.13 | 2.84 | **6.66** | 10.50 | 3.31 | 0.685 | 0.520 | 0.139 | 0.126 |
| MyAPC | 6.55 | **7.22** | 7.93 | 0.31 | 2.85 | **5.51** | 7.72 | 2.28 | 0.77 | **1.80** | 5.95 | 1.16 | 1.31 | **3.02** | 6.10 | 1.48 | 0.088 | 0.129 | 0.001 | 0.015 |
| DC | 3.72 | **4.33** | 4.63 | 0.42 | 2.20 | **2.76** | 3.04 | 0.34 | 1.16 | **1.55** | 3.41 | 0.51 | 1.39 | **2.86** | 3.94 | 1.05 | 0.004 | 0.009 | 0.001 | 0.823 |
| ncMyC | 6.03 | **9.70** | 11.80 | 3.11 | 3.71 | **6.48** | 10.50 | 0.95 | 2.79 | **4.23** | 7.04 | 0.96 | 2.53 | **15.10** | 30.20 | 11.13 | 0.167 | 0.019 | 0.002 | 0.136 |
| Count × 10^4^ |  |  |  |  |  |  |  |  |  |  |  |  |  |  |  |  |  |  |  |  |
| NKT | 68 | **182** | 342 | 94 | 23 | **204** | 360 | 172 | 91 | **248** | 1065 | 155 | 36 | **312** | 833 | 222 | 0.935 | 0.927 | 0.229 | 0.279 |
| NK | 2392 | **3825** | 5324 | 1758 | 293 | **1840** | 4905 | 838 | 1659 | **5089** | 7418 | 787 | 550 | **3103** | 4669 | 1215 | 0.167 | 0.002 | 0.116 | 0.412 |
| CD4^+^ T | 1516 | **2266** | 4110 | 978 | 272 | **2808** | 9810 | 1766 | 1512 | **3703** | 7900 | 946 | 528 | **4920** | 7735 | 1973 | 0.291 | 0.215 | 0.095 | 0.371 |
| CD4^+^CD25^+^ T | 193 | **210** | 420 | 26 | 35 | **579** | 1975 | 365 | 90 | **390** | 756 | 322 | 75 | **900** | 1671 | 620 | 0.088 | 0.066 | 0.267 | 0.551 |
| CD8^+^ T | 556 | **1144** | 2082 | 689 | 177 | **652** | 1835 | 482 | 654 | **1385** | 4275 | 334 | 134 | **988** | 2849 | 643 | 0.223 | 0.073 | 0.308 | 0.296 |
| DNT | 1172 | **1485** | 1995 | 298 | 578 | **1876** | 8000 | 1197 | 603 | **2289** | 3233 | 659 | 615 | **3338** | 11180 | 1617 | 0.465 | 0.012 | 0.052 | 0.117 |
| B2 | 4600 | **10300** | 11715 | 2098 | 725 | **4285** | 30780 | 426 | 3912 | **10650** | 20540 | 4325 | 552 | **4912** | 22120 | 3668 | 0.042 | 0.031 | 0.517 | 0.502 |
| CD11b^+^ B | 1690 | **2153** | 2490 | 159 | 413 | **1458** | 2860 | 1109 | 1332 | **2050** | 5453 | 933 | 148 | **924** | 5634 | 735 | 0.088 | 0.019 | 0.853 | 0.941 |
| ILC | 329 | **376** | 693 | 21 | 158 | **688** | 1445 | 417 | 315 | **731** | 1526 | 257 | 120 | **1054** | 1599 | 595 | 0.123 | 0.408 | 0.026 | 0.412 |
| PMN | 2247 | **3696** | 9680 | 1051 | 2600 | **5430** | 15990 | 3395 | 2735 | **6908** | 18240 | 4270 | 1220 | **4265** | 11310 | 2406 | 0.223 | 0.073 | 0.229 | 0.205 |
| MO | 1757 | **2260** | 4081 | 746 | 1240 | **3540** | 7315 | 312 | 2280 | **4463** | 11760 | 2116 | 887 | **2723** | 7809 | 896 | 0.223 | 0.035 | 0.026 | 0.073 |
| MΦ | 3500 | **4560** | 6540 | 1112 | 527 | **4280** | 6490 | 1164 | 1692 | **3618** | 8475 | 2191 | 1010 | **3012** | 6091 | 1246 | 0.570 | 0.154 | 0.459 | 0.146 |
| MyAPC | 2776 | **3585** | 4332 | 909 | 285 | **2755** | 5018 | 1213 | 385 | **1093** | 4463 | 476 | 407 | **1237** | 3965 | 632 | 0.123 | 0.408 | 0.010 | 0.117 |
| DC | 1425 | **2315** | 2598 | 420 | 220 | **1200** | 2484 | 471 | 396 | **879** | 2273 | 249 | 317 | **1025** | 3183 | 432 | 0.088 | 0.215 | 0.007 | 0.765 |
| ncMyC | 2952 | **3618** | 6490 | 987 | 1050 | **3020** | 4422 | 1351 | 966 | **2482** | 3623 | 648 | 1265 | **5912** | 28690 | 5211 | 0.123 | 0.031 | 0.007 | 0.117 |

Supplementary Table S2 (continued).

| **iLN** | 24 h |  |  |  |  |  |  |  | 48 h |  |  |  |  |  |  |  | p_Dunn_ |  |  |  |
| --- | --- | --- | --- | --- | --- | --- | --- | --- | --- | --- | --- | --- | --- | --- | --- | --- | --- | --- | --- | --- |
|  | GAS |  |  |  | CIA+GAS |  |  |  | GAS |  |  |  | CIA+GAS |  |  |  | Group |  | Time |  |
| % | Min | **Median** | Max | MAD | Min | **Median** | Max | MAD | Min | **Median** | Max | MAD | Min | **Median** | Max | MAD | 24 h | 48 h | GAS | CIA+GAS |
| NKT | 0.33 | **0.48** | 0.66 | 0.21 | 0.04 | **0.17** | 0.52 | 0.09 | 0.16 | **0.36** | 0.65 | 0.08 | 0.07 | **0.20** | 0.43 | 0.16 | 0.042 | 0.003 | 0.105 | 0.911 |
| NK | 1.07 | **1.26** | 1.63 | 0.28 | 0.40 | **0.98** | 6.25 | 0.65 | 0.73 | **1.13** | 2.27 | 0.30 | 0.24 | **0.95** | 1.44 | 0.26 | 0.569 | 0.056 | 0.308 | 0.478 |
| CD4^+^ T | 38.70 | **44.30** | 50.40 | 2.82 | 6.17 | **18.90** | 31.10 | 12.75 | 39.00 | **45.00** | 48.50 | 3.48 | 12.60 | **21.65** | 38.40 | 7.93 | 0.004 | <0.001 | 0.781 | 0.551 |
| CD4^+^CD25^+^ T | 1.57 | **1.78** | 2.44 | 0.31 | 0.66 | **1.74** | 2.25 | 0.76 | 1.68 | **2.37** | 3.71 | 0.57 | 1.44 | **2.20** | 4.04 | 0.43 | 0.372 | 0.505 | 0.052 | 0.021 |
| CD8^+^ T | 29.00 | **29.30** | 35.70 | 0.44 | 4.13 | **13.80** | 22.50 | 11.58 | 24.50 | **28.50** | 33.20 | 2.08 | 10.10 | **20.40** | 27.40 | 2.97 | 0.004 | <0.001 | 0.064 | 0.179 |
| DNT | 1.02 | **1.14** | 1.54 | 0.18 | 0.75 | **1.17** | 2.06 | 0.56 | 1.01 | **1.26** | 1.38 | 0.13 | 0.93 | **1.27** | 1.86 | 0.47 | 0.808 | 0.730 | 0.926 | 0.296 |
| B2 | 9.11 | **21.20** | 27.00 | 8.60 | 39.60 | **45.90** | 81.60 | 7.86 | 13.70 | **19.40** | 29.40 | 4.45 | 30.30 | **51.50** | 73.40 | 12.75 | 0.004 | <0.001 | 0.746 | 0.823 |
| CD11b^+^ B | 0.04 | **0.07** | 0.11 | 0.03 | 0.01 | **0.10** | 0.19 | 0.03 | 0.02 | **0.08** | 0.16 | 0.01 | 0.06 | **0.13** | 0.26 | 0.05 | 0.371 | 0.092 | 0.486 | 0.246 |
| ILC | 0.25 | **0.41** | 0.47 | 0.07 | 0.42 | **0.46** | 2.52 | 0.06 | 0.35 | **0.95** | 1.43 | 0.44 | 0.18 | **0.50** | 1.35 | 0.21 | 0.104 | 0.027 | 0.011 | 0.502 |
| PMN | 0.13 | **0.21** | 0.35 | 0.12 | 0.07 | **6.68** | 14.60 | 9.76 | 0.07 | **0.20** | 0.87 | 0.15 | 0.03 | **0.22** | 4.19 | 0.23 | 0.223 | 0.872 | 0.642 | 0.030 |
| MO | 0.21 | **0.29** | 0.32 | 0.04 | 0.08 | **0.59** | 1.34 | 0.73 | 0.05 | **0.19** | 0.92 | 0.12 | 0.07 | **0.13** | 0.27 | 0.05 | 0.222 | 0.160 | 0.138 | 0.044 |
| MΦ | 0.15 | **0.26** | 0.31 | 0.06 | 0.08 | **0.31** | 0.92 | 0.21 | 0.07 | **0.26** | 1.17 | 0.20 | 0.08 | **0.18** | 0.57 | 0.10 | 0.514 | 0.190 | 0.610 | 0.431 |
| MyAPC | 0.06 | **0.08** | 0.08 | 0.01 | 0.01 | **0.04** | 0.15 | 0.03 | 0.02 | **0.05** | 0.09 | 0.02 | 0.01 | **0.03** | 0.08 | 0.01 | 0.051 | 0.008 | 0.064 | 0.681 |
| DC | 0.01 | **0.02** | 0.02 | 0.00 | 0.00 | **0.01** | 0.04 | 0.01 | 0.00 | **0.02** | 0.06 | 0.01 | 0.01 | **0.02** | 0.05 | 0.01 | 0.291 | 0.679 | 1.000 | 0.192 |
| ncMyC | 0.04 | **0.07** | 0.12 | 0.04 | 0.01 | **0.06** | 0.14 | 0.06 | 0.01 | **0.01** | 0.05 | 0.01 | 0.00 | **0.06** | 0.16 | 0.07 | 0.416 | 0.010 | 0.002 | 0.881 |
| Count × 10^4^ |  |  |  |  |  |  |  |  |  |  |  |  |  |  |  |  |  |  |  |  |
| NKT | 14 | **23** | 40 | 10 | 16 | **84** | 259 | 57 | 5 | **17** | 53 | 10 | 13 | **49** | 262 | 53 | 0.028 | 0.017 | 0.517 | 0.296 |
| NK | 38 | **75** | 98 | 34 | 172 | **519** | 4500 | 482 | 20 | **54** | 204 | 28 | 65 | **376** | 878 | 271 | 0.004 | <0.001 | 0.643 | 0.101 |
| CD4^+^ T | 1161 | **2772** | 3101 | 488 | 2468 | **9900** | 13608 | 4967 | 946 | **2310** | 6058 | 898 | 2688 | **7732** | 16016 | 5828 | 0.019 | <0.001 | 0.853 | 0.456 |
| CD4^+^CD25^+^ T | 47 | **125** | 146 | 32 | 264 | **810** | 1447 | 410 | 54 | **104** | 482 | 61 | 149 | **741** | 3555 | 531 | 0.004 | <0.001 | 0.405 | 1.000 |
| CD8^+^ T | 870 | **2037** | 2051 | 21 | 1652 | **7524** | 11660 | 3790 | 612 | **1385** | 3627 | 427 | 1834 | **7006** | 15048 | 5643 | 0.019 | <0.001 | 0.853 | 0.941 |
| DNT | 31 | **65** | 108 | 52 | 274 | **569** | 1178 | 399 | 26 | **59** | 179 | 23 | 69 | **579** | 1637 | 325 | 0.004 | <0.001 | 0.781 | 0.823 |
| B2 | 273 | **858** | 1568 | 867 | 16524 | **21518** | 51452 | 7404 | 310 | **958** | 2970 | 378 | 2121 | **21800** | 52712 | 13820 | 0.004 | <0.001 | 0.643 | 0.502 |
| CD11b^+^ B | 1 | **3** | 6 | 3 | 6 | **40** | 137 | 25 | 1 | **4** | 18 | 2 | 6 | **42** | 114 | 37 | 0.007 | <0.001 | 0.643 | 0.941 |
| ILC | 11 | **15** | 33 | 6 | 155 | **233** | 1084 | 116 | 7 | **43** | 157 | 18 | 44 | **208** | 513 | 163 | 0.004 | <0.001 | 0.016 | 0.179 |
| PMN | 5 | **9** | 22 | 6 | 31 | **3540** | 9720 | 5198 | 3 | **11** | 44 | 9 | 4 | **70** | 1592 | 93 | 0.004 | 0.005 | 0.711 | 0.021 |
| MO | 6 | **17** | 22 | 7 | 35 | **212** | 1018 | 263 | 3 | **11** | 30 | 10 | 8 | **43** | 123 | 30 | 0.004 | <0.001 | 0.711 | 0.025 |
| MΦ | 5 | **15** | 22 | 9 | 33 | **124** | 662 | 135 | 4 | **10** | 59 | 8 | 24 | **70** | 217 | 43 | 0.004 | 0.001 | 0.578 | 0.156 |
| MyAPC | 2 | **5** | 6 | 2 | 6 | **14** | 108 | 10 | 1 | **3** | 8 | 1 | 3 | **9** | 32 | 8 | 0.007 | <0.001 | 0.459 | 0.332 |
| DC | 0 | **1** | 1 | 0 | 2 | **5** | 31 | 5 | 0 | **1** | 2 | 1 | 1 | **6** | 26 | 5 | 0.004 | <0.001 | 0.926 | 1.000 |
| ncMyC | 1 | **4** | 6 | 1 | 4 | **21** | 101 | 21 | 0 | **1** | 5 | 0 | 0 | **24** | 106 | 31 | 0.019 | 0.001 | 0.007 | 0.941 |

Supplementary Table S3. Summary and statistics on infection time-dependent cytokine expressions among mature polymorphonuclear neutrophils (PMN) compared to immature neutrophils (IMN) based on standardized (zi) median fluorescence intensities (MFI).

| *Cytokine* | 24 h |  |  |  |  |  |  |  | 48 h |  |  |  |  |  |  |  | p_Dunn_ |  |  |  |
| --- | --- | --- | --- | --- | --- | --- | --- | --- | --- | --- | --- | --- | --- | --- | --- | --- | --- | --- | --- | --- |
| **Blood** | PMN |  |  |  | IMN |  |  |  | PMN |  |  |  | IMN |  |  |  | Group |  | Time |  |
| ­z_i_(MFI) | Min | **Median** | Max | MAD | Min | **Median** | Max | MAD | Min | **Median** | Max | MAD | Min | **Median** | Max | MAD | 24 h | 48 h | PMN | IMN |
| TNFα | -1.075 | **-0.011** | 2.179 | 0.599 | -1.186 | **-0.687** | 1.406 | 0.671 | -0.952 | **-0.157** | 2.532 | 0.776 | -1.429 | **-0.512** | 2.453 | 0.560 | 0.069 | 0.049 | 0.779 | 0.425 |
| IFNγ | -0.727 | **-0.067** | 1.640 | 0.967 | -1.639 | **-0.915** | 1.602 | 0.991 | -1.096 | **0.157** | 2.530 | 1.054 | -1.655 | **-0.509** | 1.480 | 1.042 | 0.043 | 0.042 | 0.779 | 0.316 |
| IL-6 | -0.879 | **0.878** | 2.184 | 1.557 | -1.139 | **-0.615** | 0.093 | 0.217 | -1.466 | **0.346** | 2.628 | 0.807 | -1.668 | **-0.609** | 0.300 | 0.507 | 0.002 | <0.001 | 0.555 | 0.813 |
| IL-17A | -0.790 | **0.893** | 2.560 | 1.361 | -0.649 | **1.282** | 3.118 | 2.460 | -1.029 | **-0.447** | 0.532 | 0.556 | -1.152 | **-0.503** | 1.177 | 0.430 | 0.260 | 0.762 | 0.022 | 0.016 |
| IL-10 | -0.143 | **0.200** | 3.305 | 0.352 | -1.637 | **-0.313** | 2.193 | 1.102 | -0.932 | **-0.010** | 3.253 | 0.769 | -1.321 | **-0.535** | 1.904 | 0.568 | 0.028 | 0.007 | 0.114 | 0.768 |
| CCL2 | -1.307 | **-0.044** | 3.962 | 1.147 | -0.644 | **0.032** | 2.489 | 0.582 | -2.497 | **-0.430** | 1.887 | 1.210 | -1.017 | **0.200** | 1.026 | 0.669 | 0.583 | 0.045 | 0.250 | 0.883 |
| CCL3 | -1.161 | **0.165** | 1.289 | 0.879 | -1.167 | **-0.045** | 1.161 | 0.397 | -2.411 | **0.186** | 2.183 | 1.126 | -2.291 | **-0.166** | 1.484 | 1.237 | 0.386 | 0.385 | 0.848 | 0.953 |
| **Spleen** | 24 h |  |  |  |  |  |  |  | 48 h |  |  |  |  |  |  |  | p_Dunn_ |  |  |  |
|  | PMN |  |  |  | IMN |  |  |  | PMN |  |  |  | IMN |  |  |  | Group |  | Time |  |
| ­z_i_(MFI) | Min | **Median** | Max | MAD | Min | **Median** | Max | MAD | Min | **Median** | Max | MAD | Min | **Median** | Max | MAD | 24 h | 48 h | PMN | IMN |
| TNFα | -0.712 | **-0.196** | 2.176 | 0.282 | -0.910 | **-0.582** | 1.312 | 0.425 | -0.940 | **-0.092** | 2.482 | 0.688 | -1.139 | **-0.550** | 2.918 | 0.509 | 0.106 | 0.044 | 1 | 0.848 |
| IFNγ | -1.530 | **-0.232** | 1.001 | 0.415 | -0.979 | **-0.124** | 2.004 | 1.011 | -1.658 | **-0.219** | 2.482 | 0.818 | -1.649 | **-0.138** | 3.064 | 1.031 | 0.453 | 0.941 | 0.825 | 0.497 |
| IL-6 | -1.307 | **1.380** | 2.557 | 1.670 | -0.942 | **0.103** | 0.727 | 0.462 | -1.307 | **-0.195** | 2.441 | 0.556 | -1.275 | **-0.706** | 0.264 | 0.397 | 0.083 | 0.001 | 0.101 | 0.001 |
| IL-17A | -1.110 | **1.034** | 2.728 | 1.671 | -1.435 | **-0.228** | 2.047 | 0.993 | -1.669 | **-0.174** | 1.884 | 0.866 | -2.048 | **-0.332** | 2.800 | 0.421 | 0.225 | 0.346 | 0.136 | 0.768 |
| IL-10 | -0.531 | **0.365** | 1.985 | 0.844 | -1.163 | **-0.749** | 0.997 | 0.484 | -1.417 | **0.134** | 3.330 | 1.031 | -1.585 | **-0.471** | 1.619 | 0.690 | 0.008 | 0.040 | 0.345 | 0.575 |
| CCL2 | -1.376 | **-0.449** | 1.224 | 0.720 | -1.548 | **-0.059** | 1.147 | 0.230 | -2.924 | **-0.391** | 1.872 | 1.058 | -1.376 | **0.415** | 1.953 | 0.935 | 0.386 | 0.024 | 0.859 | 0.128 |
| CCL3 | -0.944 | **0.338** | 1.847 | 0.818 | -1.004 | **0.568** | 1.933 | 0.976 | -2.591 | **-0.022** | 1.755 | 0.693 | -2.387 | **0.156** | 1.173 | 0.764 | 0.862 | 0.617 | 0.140 | 0.140 |
| **Liver** | 24 h |  |  |  |  |  |  |  | 48 h |  |  |  |  |  |  |  | p_Dunn_ |  |  |  |
|  | PMN |  |  |  | IMN |  |  |  | PMN |  |  |  | IMN |  |  |  | Group |  | Time |  |
| ­z_i_(MFI) | Min | **Median** | Max | MAD | Min | **Median** | Max | MAD | Min | **Median** | Max | MAD | Min | **Median** | Max | MAD | 24 h | 48 h | PMN | IMN |
| TNFα | -1.352 | **-0.363** | 1.829 | 0.819 | -2.006 | **-1.002** | 1.205 | 0.885 | -1.002 | **0.573** | 2.455 | 0.885 | -1.963 | **-0.251** | 1.009 | 0.728 | 0.053 | <0.001 | 0.067 | 0.059 |
| IFNγ | -1.148 | **-0.026** | 2.283 | 0.956 | -1.435 | **-1.059** | 2.056 | 0.265 | -0.832 | **0.341** | 2.848 | 0.577 | -1.321 | **-0.808** | 2.723 | 0.559 | 0.006 | 0.001 | 0.175 | 0.027 |
| IL-6 | -1.158 | **1.198** | 2.680 | 1.675 | -0.820 | **-0.487** | 0.318 | 0.271 | -0.826 | **0.016** | 2.857 | 0.703 | -1.027 | **-0.636** | -0.003 | 0.286 | 0.003 | <0.001 | 0.128 | 0.084 |
| IL-17A | -1.134 | **0.216** | 0.679 | 0.470 | 0.065 | **1.667** | 2.637 | 1.178 | -2.279 | **-0.629** | 0.605 | 0.817 | -0.996 | **0.153** | 1.489 | 0.637 | 0.008 | <0.001 | 0.002 | 0.001 |
| IL-10 | -0.725 | **0.784** | 2.519 | 0.738 | -1.521 | **-0.817** | 2.212 | 0.266 | -1.393 | **0.420** | 2.251 | 1.269 | -1.489 | **-0.677** | 1.602 | 0.604 | 0.001 | 0.001 | 0.144 | 0.535 |
| CCL2 | -2.179 | **-0.377** | 0.334 | 0.619 | -1.130 | **0.311** | 1.647 | 0.508 | -3.282 | **-0.461** | 1.708 | 1.022 | -0.612 | **0.378** | 2.545 | 0.518 | 0.002 | <0.001 | 0.679 | 0.616 |
| CCL3 | -1.162 | **-0.481** | 1.393 | 0.502 | -1.207 | **-0.659** | 0.138 | 0.370 | -1.977 | **0.626** | 2.221 | 1.247 | -1.893 | **-0.009** | 1.959 | 0.956 | 0.166 | 0.219 | 0.063 | 0.027 |
| **iLN** | 24 h |  |  |  |  |  |  |  | 48 h |  |  |  |  |  |  |  | p_Dunn_ |  |  |  |
|  | PMN |  |  |  | IMN |  |  |  | PMN |  |  |  | IMN |  |  |  | Group |  | Time |  |
| ­z_i_(MFI) | Min | **Median** | Max | MAD | Min | **Median** | Max | MAD | Min | **Median** | Max | MAD | Min | **Median** | Max | MAD | 24 h | 48 h | PMN | IMN |
| TNFα | -0.524 | **-0.038** | 2.876 | 0.407 | -1.936 | **-0.334** | 1.944 | 1.326 | -0.749 | **0.129** | 1.598 | 0.732 | -2.179 | **-0.359** | 1.106 | 0.529 | 0.225 | 0.008 | 0.701 | 0.672 |
| IFNγ | -0.899 | **0.774** | 1.458 | 0.529 | -1.321 | **-0.558** | 0.566 | 0.190 | -0.952 | **-0.282** | 2.436 | 0.474 | -1.459 | **-0.343** | 3.170 | 0.995 | 0.018 | 0.411 | 0.059 | 0.551 |
| IL-6 | -0.731 | **0.807** | 2.146 | 0.858 | -1.185 | **-0.290** | 0.586 | 0.588 | -1.266 | **0.189** | 2.660 | 1.389 | -1.985 | **-0.629** | 0.458 | 0.484 | 0.008 | 0.001 | 0.204 | 0.048 |
| IL-17A | -1.089 | **0.386** | 1.439 | 0.506 | -1.306 | **0.386** | 2.857 | 0.688 | -1.135 | **-0.229** | 0.772 | 0.667 | -2.831 | **0.168** | 3.137 | 0.514 | 0.954 | 0.171 | 0.031 | 0.233 |
| IL-10 | -0.593 | **0.440** | 2.658 | 0.299 | -1.248 | **-0.542** | 0.643 | 0.629 | -0.750 | **0.063** | 2.389 | 0.551 | -1.413 | **-0.588** | 0.885 | 0.363 | 0.013 | <0.001 | 0.035 | 0.777 |
| CCL2 | -1.301 | **0.091** | 1.061 | 0.547 | -0.963 | **-0.029** | 2.151 | 1.151 | -2.305 | **-0.255** | 1.900 | 1.036 | -2.925 | **0.027** | 1.884 | 1.131 | 0.954 | 0.931 | 0.400 | 0.510 |
| CCL3 | -0.150 | **0.270** | 1.378 | 0.571 | -0.732 | **-0.157** | 1.274 | 0.458 | -2.272 | **0.035** | 0.781 | 0.460 | -2.860 | -0.090 | 0.910 | 0.832 | 0.057 | 0.545 | 0.042 | 0.451 |

Supplementary Table S4. Summary and statistics on frequency data from manually gated and FlowSOM-generated bone marrow stem and progenitor cell populations.

| *Population* | 24 h |  |  |  |  |  |  |  | 48 h |  |  |  |  |  |  |  | p_Dunn_ |  |  |  |
| --- | --- | --- | --- | --- | --- | --- | --- | --- | --- | --- | --- | --- | --- | --- | --- | --- | --- | --- | --- | --- |
| % | GAS |  |  |  | CIA+GAS |  |  |  | GAS |  |  |  | CIA+GAS |  |  |  | Group |  | Time |  |
| of Lin^lo/‒^ | Min | **Median** | Max | MAD | Min | **Median** | Max | MAD | Min | **Median** | Max | MAD | Min | **Median** | Max | MAD | 24 h | 48 h | GAS | CIA+GAS |
| MPP | 1.17 | **2.70** | 3.50 | 0.36 | 1.03 | **3.10** | 6.73 | 0.89 | 0.58 | **1.37** | 11.30 | 0.76 | 0.67 | **4.14** | 6.62 | 1.87 | 0.685 | 0.467 | 0.459 | 0.322 |
| MPP_MkE_ | 0.08 | **0.18** | 0.24 | 0.06 | 0.04 | **0.14** | 0.20 | 0.07 | 0.03 | **0.18** | 0.47 | 0.14 | 0.03 | **0.53** | 1.17 | 0.37 | 0.415 | 0.008 | 1.000 | 0.029 |
| MPP_GM_ | 1.10 | **1.33** | 1.58 | 0.34 | 0.78 | **1.41** | 1.50 | 0.13 | 0.61 | **1.27** | 5.29 | 0.95 | 0.60 | **4.05** | 8.13 | 1.28 | 0.368 | 0.026 | 0.610 | 0.035 |
| MPP_Ly_ | 0.90 | **1.35** | 1.83 | 0.30 | 0.59 | **1.06** | 2.14 | 0.70 | 0.89 | **1.53** | 2.21 | 0.38 | 0.80 | **1.41** | 3.12 | 0.49 | 0.685 | 0.752 | 0.331 | 0.322 |
| ST-HSC | 0.84 | **1.15** | 1.83 | 0.46 | 0.86 | **1.42** | 1.73 | 0.34 | 0.40 | **1.11** | 2.65 | 0.70 | 0.57 | **3.01** | 4.08 | 1.20 | 0.685 | 0.012 | 0.781 | 0.052 |
| LT-HSC | 0.36 | **0.37** | 0.73 | 0.01 | 0.29 | **0.39** | 0.76 | 0.15 | 0.17 | **0.51** | 1.94 | 0.36 | 0.25 | **0.91** | 1.90 | 0.39 | 0.807 | 0.069 | 0.817 | 0.043 |
| CMP | 0.08 | **0.16** | 0.37 | 0.12 | 0.00 | **0.10** | 0.70 | 0.12 | 0.00 | **0.20** | 1.15 | 0.30 | 0.00 | **0.00** | 1.00 | 0.00 | 0.329 | 0.035 | 0.643 | 0.089 |
| GMP | 1.54 | **3.55** | 5.52 | 2.02 | 0.05 | **2.62** | 7.53 | 3.19 | 0.09 | **4.65** | 9.06 | 3.81 | 0.00 | **0.04** | 7.96 | 0.04 | 0.685 | 0.003 | 0.711 | 0.036 |
| CLP | 0.15 | **0.21** | 0.30 | 0.09 | 0.08 | **0.18** | 0.24 | 0.06 | 0.07 | **0.17** | 0.32 | 0.09 | 0.09 | **0.16** | 0.34 | 0.01 | 0.370 | 0.643 | 0.286 | 0.551 |
| of KIT^lo/+^ |  |  |  |  |  |  |  |  |  |  |  |  |  |  |  |  |  |  |  |  |
| ^φ^MPP | 1.04 | **3.24** | 3.88 | 0.30 | 0.57 | **3.20** | 9.34 | 2.58 | 0.02 | **0.58** | 17.50 | 0.82 | 0.00 | **2.79** | 10.20 | 2.97 | 0.685 | 0.497 | 0.228 | 0.501 |
| ^φ^HPC_i_ | 5.17 | **5.98** | 7.54 | 0.80 | 2.74 | **5.48** | 16.60 | 3.68 | 1.75 | **4.51** | 20.90 | 2.60 | 2.52 | **32.10** | 45.00 | 6.67 | 0.685 | 0.004 | 0.308 | 0.016 |
| ^φ^HPC_ii_ | 17.20 | **20.00** | 21.50 | 2.22 | 11.60 | **23.90** | 27.30 | 3.71 | 11.90 | **23.05** | 27.80 | 2.74 | 14.60 | **19.60** | 25.90 | 4.30 | 0.167 | 0.099 | 0.037 | 0.302 |
| ^φ^HPC_iii_ | 0.37 | **0.52** | 1.02 | 0.22 | 0.46 | **0.84** | 2.83 | 0.37 | 0.32 | **0.85** | 4.18 | 0.44 | 0.22 | **7.01** | 13.20 | 3.57 | 0.167 | 0.005 | 0.165 | 0.022 |
| ^φ^HSC | 6.30 | **6.86** | 7.46 | 0.25 | 3.85 | **6.99** | 8.68 | 1.02 | 3.92 | **7.49** | 11.80 | 2.22 | 1.78 | **5.03** | 8.98 | 2.85 | 0.685 | 0.012 | 0.459 | 0.062 |
| ^φ^GMP_i_ | 0.22 | **0.90** | 1.79 | 0.92 | 0.04 | **1.08** | 10.60 | 1.16 | 0.00 | **0.30** | 13.20 | 0.40 | 0.00 | **5.28** | 15.90 | 7.70 | 0.808 | 0.058 | 0.308 | 0.122 |
| ^φ^GMP_ii_ | 20.60 | **34.50** | 36.30 | 2.67 | 4.42 | **23.40** | 36.30 | 10.23 | 4.50 | **31.70** | 42.00 | 8.60 | 0.70 | **1.20** | 35.10 | 0.49 | 0.220 | <0.001 | 0.853 | 0.013 |
| ^φ^CLP_i_ | 0.43 | **0.50** | 0.63 | 0.10 | 0.15 | **0.46** | 2.05 | 0.28 | 0.24 | **0.75** | 1.57 | 0.21 | 0.81 | **3.46** | 6.12 | 1.13 | 0.935 | <0.001 | 0.026 | 0.001 |
| ^φ^CLP_ii_ | 5.14 | **9.14** | 18.30 | 3.94 | 3.92 | **11.90** | 22.10 | 6.38 | 2.62 | **7.34** | 23.20 | 5.86 | 2.49 | **3.86** | 9.18 | 1.66 | 0.808 | 0.037 | 0.459 | 0.004 |
| ^φ^CLP_iii_ | 10.60 | **14.20** | 16.80 | 0.44 | 7.70 | **14.10** | 21.50 | 4.00 | 0.68 | **9.81** | 20.20 | 6.37 | 0.63 | **1.16** | 18.90 | 0.65 | 0.684 | 0.024 | 0.064 | 0.010 |
| ^φ^CLP_iv_ | 4.94 | **6.30** | 6.63 | 0.49 | 4.50 | **5.81** | 9.34 | 1.13 | 3.68 | **8.19** | 11.00 | 2.88 | 3.16 | **8.73** | 13.50 | 4.70 | 0.685 | 1.000 | 0.052 | 0.104 |

Supplementary Table S5. Summary and statistics on frequency and cell count data from non-Neutrophil cytokine-producing immune cell populations in peripheral blood, spleen, liver and inguinal lymph nodes (iLN).

| *Population* | 24 h |  |  |  |  |  |  |  | 48 h |  |  |  |  |  |  |  | p_Dunn_ |  |  |  |
| --- | --- | --- | --- | --- | --- | --- | --- | --- | --- | --- | --- | --- | --- | --- | --- | --- | --- | --- | --- | --- |
| **Blood** | GAS |  |  |  | CIA+GAS |  |  |  | GAS |  |  |  | CIA+GAS |  |  |  | Group |  | Time |  |
| % | Min | **Median** | Max | MAD | Min | **Median** | Max | MAD | Min | **Median** | Max | MAD | Min | **Median** | Max | MAD | 24 h | 48 h | GAS | CIA+GAS |
| TNFα | 0.84 | **1.29** | 1.90 | 0.53 | 0.14 | **0.79** | 3.66 | 0.64 | 0.98 | **1.59** | 2.38 | 0.38 | 1.56 | **2.23** | 6.93 | 0.62 | 0.223 | 0.004 | 0.052 | 0.007 |
| IFNγ | 3.01 | **4.70** | 10.40 | 1.60 | 1.34 | **1.98** | 2.70 | 0.50 | 1.34 | **3.62** | 7.43 | 1.99 | 1.14 | **2.96** | 8.18 | 1.23 | 0.004 | 0.646 | 0.267 | 0.011 |
| IL-6 | 2.25 | **2.72** | 7.04 | 0.70 | 0.89 | **1.47** | 2.14 | 0.65 | 0.82 | **2.41** | 4.52 | 1.79 | 1.90 | **9.33** | 18.10 | 6.06 | 0.004 | <0.001 | 0.195 | <0.001 |
| IL-17A | 0.51 | **0.63** | 0.79 | 0.18 | 0.21 | **0.48** | 0.73 | 0.22 | 0.32 | **0.64** | 1.63 | 0.22 | 0.61 | **1.44** | 2.63 | 0.70 | 0.051 | 0.001 | 0.643 | <0.001 |
| IL-10 | 0.81 | **1.50** | 8.99 | 0.79 | 0.63 | **1.20** | 2.22 | 0.62 | 0.64 | **2.16** | 7.81 | 1.50 | 1.17 | **8.46** | 14.20 | 6.19 | 0.570 | 0.005 | 0.711 | 0.003 |
| CCL2 | 2.99 | **3.40** | 6.55 | 0.61 | 1.87 | **2.83** | 4.21 | 1.05 | 1.18 | **3.15** | 10.30 | 1.59 | 1.90 | **10.90** | 24.70 | 9.13 | 0.062 | 0.004 | 0.405 | 0.009 |
| CCL3 | 0.81 | **3.85** | 4.50 | 0.96 | 0.38 | **0.63** | 2.49 | 0.31 | 0.69 | **2.45** | 6.65 | 1.60 | 0.76 | **2.60** | 6.33 | 1.21 | 0.012 | 0.818 | 0.405 | 0.002 |
| Count × 10^4^ |  |  |  |  |  |  |  |  |  |  |  |  |  |  |  |  |  |  |  |  |
| TNFα | 2.51 | **3.53** | 5.16 | 1.51 | 0.08 | **1.43** | 10.98 | 1.63 | 2.38 | **6.67** | 13.11 | 4.02 | 0.41 | **2.52** | 17.53 | 2.48 | 0.123 | 0.006 | 0.033 | 0.136 |
| IFNγ | 7.53 | **15.20** | 26.00 | 6.10 | 0.80 | **3.38** | 6.48 | 3.59 | 5.73 | **18.79** | 26.75 | 8.48 | 0.25 | **4.87** | 18.42 | 3.58 | 0.004 | <0.001 | 1.000 | 0.654 |
| IL-6 | 6.08 | **11.38** | 28.16 | 7.86 | 0.62 | **3.21** | 5.46 | 2.46 | 2.74 | **7.65** | 22.14 | 5.49 | 1.20 | **8.03** | 27.19 | 4.40 | 0.004 | 0.890 | 0.643 | 0.003 |
| IL-17A | 1.28 | **1.90** | 3.16 | 0.79 | 0.13 | **1.00** | 1.89 | 1.11 | 1.06 | **2.70** | 9.45 | 1.59 | 0.12 | **1.59** | 5.87 | 0.76 | 0.028 | 0.060 | 0.229 | 0.044 |
| IL-10 | 3.24 | **4.07** | 22.48 | 1.24 | 1.08 | **1.79** | 3.36 | 1.06 | 2.11 | **10.87** | 24.46 | 7.95 | 1.23 | **5.97** | 21.08 | 4.18 | 0.007 | 0.520 | 0.405 | 0.003 |
| CCL2 | 8.50 | **12.56** | 26.20 | 5.77 | 1.12 | **6.65** | 8.93 | 2.73 | 3.75 | **14.31** | 32.17 | 8.26 | 1.29 | **10.65** | 32.29 | 5.85 | 0.012 | 0.312 | 0.926 | 0.044 |
| CCL3 | 2.19 | **9.63** | 18.90 | 11.03 | 0.34 | **1.05** | 7.47 | 0.79 | 3.38 | **9.46** | 31.92 | 8.75 | 0.32 | **3.29** | 14.42 | 3.39 | 0.012 | 0.001 | 0.781 | 0.101 |
| **Spleen** | 24 h |  |  |  |  |  |  |  | 48 h |  |  |  |  |  |  |  | p_Dunn_ |  |  |  |
|  | GAS |  |  |  | CIA+GAS |  |  |  | GAS |  |  |  | CIA+GAS |  |  |  | Group |  | Time |  |
| % | Min | **Median** | Max | MAD | Min | **Median** | Max | MAD | Min | **Median** | Max | MAD | Min | **Median** | Max | MAD | 24 h | 48 h | GAS | CIA+GAS |
| TNFα | 2.57 | **3.41** | 3.97 | 0.73 | 2.23 | **3.73** | 4.95 | 1.01 | 2.27 | **3.25** | 4.61 | 1.16 | 3.02 | **4.83** | 8.83 | 1.59 | 0.465 | 0.002 | 0.746 | 0.073 |
| IFNγ | 3.64 | **4.36** | 5.03 | 0.28 | 2.32 | **3.47** | 5.07 | 1.51 | 2.82 | **4.03** | 5.52 | 0.67 | 2.51 | **4.11** | 8.08 | 0.93 | 0.223 | 0.818 | 0.229 | 0.314 |
| IL-6 | 1.33 | **1.40** | 1.73 | 0.10 | 1.21 | **3.36** | 5.05 | 1.22 | 0.80 | **2.69** | 4.38 | 1.25 | 1.92 | **3.07** | 7.91 | 1.29 | 0.042 | 0.118 | 0.042 | 0.823 |
| IL-17A | 1.98 | **2.79** | 4.93 | 0.80 | 3.43 | **4.01** | 5.98 | 0.62 | 1.73 | **4.78** | 9.05 | 1.25 | 2.43 | **4.24** | 9.70 | 2.58 | 0.062 | 0.945 | 0.052 | 0.654 |
| IL-10 | 2.56 | **2.73** | 3.16 | 0.15 | 3.25 | **4.33** | 6.74 | 0.95 | 1.62 | **2.74** | 8.29 | 0.79 | 2.35 | **3.90** | 7.71 | 1.96 | 0.004 | 0.041 | 0.853 | 0.478 |
| CCL2 | 3.00 | **3.43** | 3.57 | 0.18 | 4.43 | **5.10** | 7.78 | 0.99 | 3.15 | **5.13** | 9.34 | 1.86 | 4.36 | **6.37** | 11.90 | 2.25 | 0.004 | 0.108 | 0.007 | 0.314 |
| CCL3 | 5.86 | **7.29** | 9.37 | 2.12 | 3.00 | **4.05** | 7.85 | 0.90 | 4.46 | **6.89** | 27.50 | 3.36 | 1.73 | **6.44** | 9.23 | 2.79 | 0.042 | 0.141 | 0.781 | 0.371 |
| Count × 10^4^ |  |  |  |  |  |  |  |  |  |  |  |  |  |  |  |  |  |  |  |  |
| TNFα | 68.88 | **115.44** | 148.68 | 12.62 | 62.44 | **109.37** | 183.52 | 52.08 | 64.47 | **149.20** | 225.84 | 52.01 | 86.98 | **162.12** | 346.75 | 65.54 | 0.935 | 0.613 | 0.096 | 0.044 |
| IFNγ | 97.55 | **143.01** | 191.40 | 31.50 | 61.75 | **80.50** | 198.28 | 27.80 | 74.37 | **190.99** | 297.48 | 73.23 | 91.15 | **144.24** | 281.18 | 28.68 | 0.123 | 0.066 | 0.116 | 0.062 |
| IL-6 | 35.91 | **45.92** | 57.99 | 7.84 | 30.01 | **104.52** | 194.83 | 54.68 | 39.19 | **115.88** | 213.86 | 84.47 | 61.34 | **108.70** | 228.60 | 44.25 | 0.167 | 0.818 | 0.016 | 0.412 |
| IL-17A | 58.61 | **74.77** | 161.70 | 23.96 | 84.72 | **112.84** | 217.96 | 41.18 | 43.60 | **226.30** | 474.22 | 93.34 | 88.04 | **148.24** | 364.72 | 73.63 | 0.223 | 0.141 | 0.021 | 0.086 |
| IL-10 | 73.16 | **92.82** | 111.62 | 19.43 | 96.76 | **139.16** | 173.60 | 30.75 | 46.01 | **137.39** | 434.40 | 51.87 | 85.10 | **136.42** | 324.61 | 24.83 | 0.012 | 0.854 | 0.026 | 0.709 |
| CCL2 | 80.40 | **103.60** | 155.65 | 13.20 | 114.08 | **142.80** | 235.67 | 42.58 | 102.82 | **258.13** | 440.85 | 100.41 | 136.22 | **224.73** | 447.55 | 64.08 | 0.028 | 0.581 | 0.004 | 0.037 |
| CCL3 | 171.11 | **221.40** | 396.76 | 44.06 | 84.00 | **100.44** | 386.22 | 21.20 | 115.42 | **403.71** | 934.12 | 227.25 | 58.13 | **191.96** | 468.88 | 103.11 | 0.088 | 0.007 | 0.116 | 0.263 |

Supplementary Table S5 (continued).

| **Liver** | 24 h |  |  |  |  |  |  |  | 48 h |  |  |  |  |  |  |  | p_Dunn_ |  |  |  |
| --- | --- | --- | --- | --- | --- | --- | --- | --- | --- | --- | --- | --- | --- | --- | --- | --- | --- | --- | --- | --- |
|  | GAS |  |  |  | CIA+GAS |  |  |  | GAS |  |  |  | CIA+GAS |  |  |  | Group |  | Time |  |
| % | Min | **Median** | Max | MAD | Min | **Median** | Max | MAD | Min | **Median** | Max | MAD | Min | **Median** | Max | MAD | 24 h | 48 h | GAS | CIA+GAS |
| TNFα | 1.35 | **2.78** | 3.34 | 0.83 | 2.06 | **5.43** | 10.20 | 2.80 | 1.73 | **3.29** | 8.45 | 1.99 | 1.78 | **7.96** | 20.30 | 1.82 | 0.028 | 0.002 | 0.267 | 0.101 |
| IFNγ | 11.50 | **15.20** | 18.20 | 1.04 | 6.33 | **14.80** | 20.30 | 6.52 | 6.36 | **8.89** | 12.70 | 1.87 | 5.95 | **8.70** | 11.90 | 1.97 | 0.808 | 0.613 | 0.004 | 0.023 |
| IL-6 | 5.14 | **9.50** | 11.30 | 2.67 | 3.17 | **13.00** | 14.80 | 2.67 | 6.90 | **10.55** | 15.60 | 2.08 | 5.52 | **13.30** | 26.40 | 3.93 | 0.291 | 0.063 | 0.266 | 0.371 |
| IL-17A | 1.88 | **3.23** | 4.29 | 1.57 | 1.83 | **2.57** | 7.95 | 1.10 | 2.58 | **3.91** | 6.12 | 1.01 | 2.06 | **3.97** | 6.42 | 1.18 | 0.935 | 0.854 | 0.229 | 0.263 |
| IL-10 | 11.20 | **13.50** | 16.10 | 1.19 | 4.79 | **9.67** | 13.70 | 4.34 | 0.96 | **3.86** | 10.20 | 2.54 | 2.69 | **6.59** | 9.04 | 2.43 | 0.042 | 0.129 | 0.001 | 0.006 |
| CCL2 | 5.76 | **9.26** | 10.10 | 1.25 | 4.01 | **8.42** | 15.40 | 2.33 | 5.89 | **8.79** | 13.50 | 2.54 | 6.23 | **8.83** | 15.70 | 2.29 | 0.808 | 0.370 | 0.459 | 0.332 |
| CCL3 | 5.21 | **6.79** | 8.09 | 1.93 | 2.89 | **4.86** | 18.40 | 2.92 | 4.68 | **7.82** | 12.30 | 2.59 | 3.29 | **7.92** | 32.70 | 2.37 | 0.291 | 0.963 | 0.267 | 0.179 |
| Count × 10^4^ |  |  |  |  |  |  |  |  |  |  |  |  |  |  |  |  |  |  |  |  |
| TNFα | 5.40 | **10.50** | 19.62 | 7.56 | 6.56 | **18.54** | 56.10 | 7.21 | 11.11 | **16.67** | 63.38 | 7.58 | 6.91 | **37.82** | 95.00 | 30.98 | 0.167 | 0.043 | 0.079 | 0.156 |
| IFNγ | 46.00 | **76.00** | 100.10 | 30.17 | 6.33 | **74.00** | 131.95 | 44.40 | 28.59 | **53.37** | 93.75 | 15.25 | 10.10 | **40.00** | 113.05 | 16.61 | 0.935 | 0.129 | 0.116 | 0.117 |
| IL-6 | 20.56 | **38.50** | 67.80 | 20.39 | 13.00 | **44.40** | 94.25 | 23.53 | 26.88 | **53.49** | 90.75 | 26.17 | 14.10 | **49.95** | 250.80 | 33.43 | 0.808 | 0.927 | 0.195 | 0.502 |
| IL-17A | 7.52 | **11.31** | 24.90 | 5.61 | 2.57 | **17.37** | 32.18 | 9.81 | 12.30 | **22.19** | 31.50 | 9.93 | 4.51 | **19.46** | 37.91 | 9.71 | 0.808 | 0.476 | 0.116 | 0.602 |
| IL-10 | 54.00 | **56.35** | 79.80 | 3.48 | 8.74 | **50.40** | 82.55 | 36.99 | 5.76 | **19.50** | 56.10 | 13.13 | 6.73 | **31.48** | 61.94 | 11.42 | 0.291 | 0.383 | 0.002 | 0.179 |
| CCL2 | 25.04 | **32.41** | 60.60 | 10.93 | 7.59 | **36.09** | 100.10 | 19.17 | 21.24 | **48.76** | 76.50 | 22.24 | 10.10 | **45.08** | 149.15 | 28.07 | 0.808 | 0.696 | 0.229 | 0.502 |
| CCL3 | 20.84 | **27.10** | 48.54 | 9.28 | 2.89 | **31.59** | 92.00 | 27.80 | 22.23 | **38.38** | 92.25 | 18.62 | 6.92 | **40.28** | 114.45 | 26.56 | 0.808 | 0.927 | 0.195 | 0.502 |
| **iLN** | 24 h |  |  |  |  |  |  |  | 48 h |  |  |  |  |  |  |  | p_Dunn_ |  |  |  |
|  | GAS |  |  |  | CIA+GAS |  |  |  | GAS |  |  |  | CIA+GAS |  |  |  | Group |  | Time |  |
| % | Min | **Median** | Max | MAD | Min | **Median** | Max | MAD | Min | **Median** | Max | MAD | Min | **Median** | Max | MAD | 24 h | 48 h | GAS | CIA+GAS |
| TNFα | 1.38 | **1.53** | 2.44 | 0.12 | 1.24 | **1.98** | 3.29 | 1.10 | 1.39 | **2.08** | 3.60 | 0.77 | 1.33 | **2.46** | 3.46 | 0.61 | 0.123 | 0.679 | 0.086 | 0.881 |
| IFNγ | 1.44 | **1.84** | 2.12 | 0.25 | 2.13 | **2.98** | 3.61 | 0.33 | 1.11 | **2.28** | 3.68 | 1.13 | 1.34 | **2.92** | 6.05 | 1.11 | 0.004 | 0.027 | 0.287 | 0.681 |
| IL-6 | 1.13 | **1.96** | 3.20 | 0.65 | 1.71 | **3.19** | 4.19 | 1.42 | 1.05 | **2.67** | 4.89 | 1.60 | 1.88 | **3.83** | 6.33 | 1.94 | 0.123 | 0.118 | 0.139 | 0.167 |
| IL-17A | 7.14 | **10.50** | 16.60 | 1.65 | 2.24 | **3.62** | 7.17 | 1.84 | 3.93 | **7.50** | 12.70 | 2.06 | 3.13 | **4.98** | 6.63 | 1.21 | 0.007 | 0.001 | 0.064 | 0.086 |
| IL-10 | 1.68 | **2.28** | 2.69 | 0.37 | 1.15 | **3.58** | 5.15 | 0.74 | 1.25 | **2.79** | 3.98 | 0.87 | 1.85 | **2.49** | 3.64 | 0.62 | 0.042 | 0.550 | 0.355 | 0.025 |
| CCL2 | 1.82 | **2.22** | 2.60 | 0.55 | 1.60 | **3.28** | 4.14 | 1.28 | 1.70 | **2.56** | 4.61 | 0.96 | 1.10 | **3.32** | 4.25 | 1.13 | 0.223 | 0.730 | 0.287 | 0.654 |
| CCL3 | 6.76 | **10.10** | 10.90 | 1.19 | 4.16 | **6.34** | 10.40 | 1.65 | 2.30 | **6.00** | 9.27 | 2.49 | 2.61 | **5.00** | 14.10 | 2.64 | 0.088 | 0.783 | 0.021 | 0.737 |
| Count × 10^4^ |  |  |  |  |  |  |  |  |  |  |  |  |  |  |  |  |  |  |  |  |
| TNFα | 0.46 | **0.87** | 1.10 | 0.20 | 5.33 | **7.56** | 22.42 | 3.30 | 0.39 | **0.99** | 3.75 | 0.54 | 1.94 | **8.18** | 17.95 | 7.24 | 0.004 | <0.001 | 0.355 | 0.332 |
| IFNγ | 0.43 | **1.10** | 1.48 | 0.56 | 7.67 | **14.44** | 24.77 | 6.68 | 0.42 | **1.06** | 3.56 | 0.74 | 0.94 | **13.08** | 24.38 | 6.24 | 0.004 | <0.001 | 0.459 | 0.654 |
| IL-6 | 0.67 | **0.91** | 1.37 | 0.18 | 8.03 | **11.95** | 29.26 | 5.82 | 0.43 | **1.50** | 4.46 | 1.00 | 1.32 | **13.36** | 48.49 | 7.22 | 0.004 | <0.001 | 0.165 | 0.941 |
| IL-17A | 2.82 | **5.00** | 9.96 | 3.23 | 8.96 | **18.09** | 38.00 | 12.30 | 1.57 | **3.66** | 13.26 | 1.50 | 3.35 | **17.89** | 48.31 | 15.45 | 0.012 | <0.001 | 0.355 | 0.881 |
| IL-10 | 0.50 | **1.36** | 1.77 | 0.62 | 4.95 | **16.32** | 39.14 | 6.05 | 0.55 | **1.17** | 4.38 | 0.56 | 1.79 | **8.65** | 20.77 | 6.85 | 0.004 | <0.001 | 0.853 | 0.052 |
| CCL2 | 0.59 | **1.27** | 1.56 | 0.42 | 6.88 | **13.20** | 29.81 | 9.37 | 0.48 | **1.28** | 4.90 | 0.72 | 2.35 | **10.81** | 37.40 | 9.43 | 0.004 | <0.001 | 0.355 | 0.296 |
| CCL3 | 2.03 | **4.11** | 7.56 | 3.09 | 17.52 | **30.85** | 74.88 | 7.78 | 0.76 | **2.79** | 11.70 | 1.02 | 3.37 | **18.20** | 72.80 | 18.02 | 0.004 | <0.001 | 0.308 | 0.205 |

Supplementary Table S6. Summary and statistics on frequency and cell count data from manually gated cell populations in paw joints.

| *Population* | 24 h |  |  |  |  |  |  |  | 48 h |  |  |  |  |  |  |  | p_Dunn_ |  |  |  |
| --- | --- | --- | --- | --- | --- | --- | --- | --- | --- | --- | --- | --- | --- | --- | --- | --- | --- | --- | --- | --- |
|  | GAS |  |  |  | CIA+GAS |  |  |  | GAS |  |  |  | CIA+GAS |  |  |  | Group |  | Time |  |
| % | Min | **Median** | Max | MAD | Min | **Median** | Max | MAD | Min | **Median** | Max | MAD | Min | **Median** | Max | MAD | 24 h | 48 h | GAS | CIA+GAS |
| EC | 3.14 | **3.42** | 3.96 | 0.16 | 2.39 | **4.45** | 6.59 | 1.23 | 1.66 | **4.30** | 7.29 | 1.39 | 2.87 | **4.99** | 7.79 | 1.28 | 0.088 | 0.748 | 0.079 | 0.370 |
| CD4^+^T | 2.90 | **3.86** | 4.61 | 0.06 | 3.63 | **6.02** | 9.92 | 1.32 | 0.87 | **4.68** | 7.26 | 0.87 | 2.17 | **4.46** | 10.70 | 1.26 | 0.028 | 0.982 | 0.138 | 0.179 |
| CD4^+^CD25^+^ T | 0.39 | **0.71** | 0.91 | 0.25 | 0.90 | **1.53** | 2.89 | 0.27 | 0.20 | **0.96** | 1.48 | 0.37 | 0.50 | **1.27** | 2.46 | 0.58 | 0.007 | 0.048 | 0.116 | 0.455 |
| B2 | 0.05 | **0.07** | 0.40 | 0.04 | 0.16 | **0.29** | 0.56 | 0.09 | 0.03 | **0.12** | 0.26 | 0.06 | 0.10 | **0.21** | 0.56 | 0.13 | 0.061 | 0.007 | 0.487 | 0.411 |
| CD11b^+^ B | 0.41 | **0.64** | 1.02 | 0.24 | 0.56 | **1.05** | 1.48 | 0.31 | 0.33 | **0.78** | 1.59 | 0.25 | 0.33 | **0.82** | 1.35 | 0.38 | 0.051 | 0.945 | 0.405 | 0.192 |
| ILC | 0.21 | **0.32** | 0.34 | 0.03 | 0.30 | **0.48** | 0.92 | 0.24 | 0.11 | **0.28** | 0.53 | 0.10 | 0.11 | **0.31** | 0.73 | 0.16 | 0.018 | 0.334 | 0.926 | 0.044 |
| PMN | 0.48 | **0.62** | 1.29 | 0.21 | 1.21 | **9.72** | 16.10 | 8.87 | 0.36 | **0.66** | 2.25 | 0.27 | 0.22 | **2.79** | 20.00 | 3.63 | 0.007 | 0.077 | 0.926 | 0.086 |
| MO | 1.50 | **1.90** | 2.63 | 0.36 | 1.50 | **2.78** | 3.68 | 0.89 | 0.51 | **1.64** | 2.80 | 0.61 | 0.64 | **1.65** | 5.56 | 0.75 | 0.087 | 0.800 | 0.267 | 0.037 |
| MΦ | 0.91 | **1.03** | 2.09 | 0.18 | 1.09 | **1.72** | 2.06 | 0.28 | 0.69 | **1.32** | 2.57 | 0.43 | 0.52 | **1.32** | 2.26 | 0.36 | 0.123 | 0.927 | 0.677 | 0.062 |
| MyAPC | 0.08 | **0.11** | 0.13 | 0.03 | 0.07 | **0.22** | 0.44 | 0.18 | 0.03 | **0.10** | 0.23 | 0.06 | 0.03 | **0.10** | 0.45 | 0.04 | 0.122 | 0.713 | 0.709 | 0.057 |
| DC | 0.19 | **0.23** | 0.36 | 0.01 | 0.10 | **0.31** | 0.54 | 0.12 | 0.05 | **0.17** | 0.27 | 0.06 | 0.06 | **0.20** | 0.65 | 0.11 | 0.220 | 0.505 | 0.029 | 0.040 |
| ncMyC | 0.13 | **0.20** | 0.26 | 0.01 | 0.09 | **0.28** | 0.45 | 0.18 | 0.05 | **0.12** | 0.29 | 0.05 | 0.05 | **0.26** | 0.64 | 0.10 | 0.464 | 0.011 | 0.020 | 0.709 |
| Count × 10^4^ |  |  |  |  |  |  |  |  |  |  |  |  |  |  |  |  |  |  |  |  |
| EC | 1.63 | **3.49** | 3.56 | 0.11 | 3.03 | **4.20** | 8.57 | 1.75 | 0.71 | **1.77** | 4.07 | 0.65 | 1.37 | **3.68** | 8.95 | 1.84 | 0.123 | 0.009 | 0.165 | 0.502 |
| CD4^+^T | 2.01 | **3.90** | 4.15 | 0.37 | 3.34 | **6.77** | 10.52 | 3.63 | 0.26 | **1.98** | 7.55 | 1.47 | 1.04 | **3.21** | 16.69 | 2.50 | 0.019 | 0.066 | 0.165 | 0.101 |
| CD4^+^CD25^+^ T | 0.37 | **0.57** | 0.93 | 0.30 | 0.83 | **1.96** | 3.06 | 0.31 | 0.06 | **0.42** | 1.22 | 0.30 | 0.24 | **0.99** | 4.53 | 0.77 | 0.007 | 0.017 | 0.308 | 0.205 |
| B2 | 0.04 | **0.07** | 0.38 | 0.05 | 0.15 | **0.35** | 0.74 | 0.08 | 0.01 | **0.05** | 0.25 | 0.05 | 0.05 | **0.18** | 0.58 | 0.19 | 0.062 | 0.003 | 0.308 | 0.296 |
| CD11b^+^ B | 0.33 | **0.59** | 0.92 | 0.33 | 0.57 | **1.11** | 2.46 | 0.64 | 0.10 | **0.30** | 1.65 | 0.22 | 0.10 | **0.72** | 2.48 | 0.60 | 0.088 | 0.103 | 0.116 | 0.179 |
| ILC | 0.11 | **0.29** | 0.33 | 0.06 | 0.28 | **0.70** | 1.21 | 0.36 | 0.03 | **0.12** | 0.42 | 0.06 | 0.03 | **0.28** | 0.85 | 0.24 | 0.027 | 0.011 | 0.064 | 0.037 |
| PMN | 0.32 | **0.56** | 1.16 | 0.35 | 1.11 | **11.86** | 26.73 | 7.28 | 0.12 | **0.36** | 1.74 | 0.26 | 0.11 | **2.49** | 18.00 | 3.53 | 0.007 | 0.022 | 0.165 | 0.044 |
| MO | 0.99 | **1.76** | 2.37 | 0.34 | 1.02 | **3.50** | 6.11 | 1.25 | 0.15 | **0.60** | 2.51 | 0.48 | 0.28 | **1.60** | 5.00 | 1.61 | 0.042 | 0.073 | 0.064 | 0.062 |
| MΦ | 0.53 | **0.96** | 2.01 | 0.63 | 1.15 | **2.10** | 3.42 | 1.22 | 0.21 | **0.48** | 2.67 | 0.36 | 0.18 | **0.99** | 4.09 | 0.65 | 0.062 | 0.081 | 0.116 | 0.062 |
| MyAPC | 0.06 | **0.09** | 0.14 | 0.05 | 0.07 | **0.27** | 0.71 | 0.26 | 0.01 | **0.05** | 0.24 | 0.05 | 0.02 | **0.09** | 0.41 | 0.10 | 0.062 | 0.073 | 0.052 | 0.044 |
| DC | 0.12 | **0.21** | 0.37 | 0.04 | 0.09 | **0.36** | 0.90 | 0.21 | 0.01 | **0.06** | 0.22 | 0.04 | 0.02 | **0.14** | 0.66 | 0.16 | 0.167 | 0.118 | 0.012 | 0.086 |
| ncMyC | 0.10 | **0.20** | 0.23 | 0.04 | 0.08 | **0.21** | 0.66 | 0.19 | 0.02 | **0.05** | 0.21 | 0.03 | 0.02 | **0.22** | 0.90 | 0.25 | 0.465 | 0.008 | 0.005 | 0.502 |
